# What’s hot and what's not in lay psychology: Wikipedia’s most-viewed articles

**DOI:** 10.1007/s12144-022-03826-0

**Published:** 2022-10-12

**Authors:** Kaśmir Ciechanowski, Natalia Banasik-Jemielniak, Dariusz Jemielniak

**Affiliations:** 1 MINDS (Management in Networked and Digital Societies) Department, Kozminski University, Warsaw, Poland; 2 Department of Psychology, Maria Grzegorzewska University, Warsaw, Poland

**Keywords:** Wikipedia, Lay psychology, Cross-cultural online behavior

## Abstract

We studied views of articles about psychology on 10 language editions of Wikipedia from July 1, 2015, to January 6, 2021. We were most interested in what psychology topics Wikipedia users wanted to read, and how the frequency of views changed during the COVID-19 pandemic and lockdowns. Our results show that the topics of interest to people seeking psychological knowledge changed during the pandemic. In addition, the interests differ noticeably among the languages. We made two important observations. The first was that during the pandemic, people in most countries looked for new ways to manage their stress without resorting to external help. This is understandable, given the increased stress of lockdown and the limited amount of professional help available. We also found that academic topics, typically covered in university classes, experienced a substantial drop in traffic, which could be indicative of issues with remote teaching.

**Supplementary Information:**

The online version contains supplementary material available at 10.1007/s12144-022-03826-0.

## Introduction

Possibly because of ease of access, the internet has become the default source of knowledge (Kibirige & DePalo, 2017). People go online to find information on highly specialized topics such as mental health, too (Colditz et al., 2018; Fergie et al., 2016).

Wikipedia is arguably the most popular text-based online resource, and the fifth most popular website in the world (Armstrong, 2019; Netimperative, 2019). Although still treated with suspicion by academia (Aibar et al., 2015; Jemielniak, 2019; Jemielniak & Aibar, 2016), it has already become a prominent source of academic knowledge. Even though Wikipedia is crowdsourced and relies on a participatory governance model (Jemielniak, 2016; Konieczny, 2017), its reliability is comparable to peer-reviewed sources. In 2005, it went “head to head” with the *Encyclopedia Britannica*, according to a study published in *Nature* (Giles, 2005). Even though the standards of presentation of knowledge differ among the 300 language editions (Jemielniak & Wilamowski, 2017), Wikipedia’s quality is considered high (James, 2016; Mesgari et al., 2015; Michelucci & Dickinson, 2016), even for specialized topics such as psychology or psychiatry (Reavley et al., 2012).

Psychology articles on Wikipedia are well organized thanks to Wikiproject Psychology, a volunteer-driven initiative focused on curating articles. Research shows that Wikipedia’s psychology articles rely on top-tier academic references, and are generally well sourced (Banasik-Jemielniak et al., 2021). We study variations in the popularity of these articles over time.

The aim of the study was to investigate changes in the popularity of Wikipedia’s articles on psychology during the COVID-19 pandemic. We also wanted to identify any significant differences among the major languages used in Wikipedia when it comes to the popularity of entries, and any trends in the number of articles viewed over time. Studies have shown that interest in articles about medicine during the COVID-19 pandemic fluctuated significantly (Chrzanowski et al., 2021).

There is other research that shows that Google Trends is a good predictor of disease outbreaks and general behavioral patterns (Heerfordt & Heerfordt, 2020; Nuti et al., 2014), or of mental health and psychological well-being in the long term after the pandemic (Hoerger et al., 2020; Zattoni et al., 2020). Analyzing search patterns helped researchers to find that depression, anxiety, and stress increased in Bangladeshi students during the pandemic (Al Mamun et al., 2021). There are other studies which analyze web traffic of stress-related queries (Silverio-Murillo et al., 2021), mental health queries used for real-time surveillance mental health queries used for real-time surveillance (Hoerger et al., 2020; Knipe et al., 2020), or “insomnia” (Zitting et al., 2021). There is even one study that tries to determine the impact of the pandemic on people’s mental health with the use of Google Trends and social media (Gianfredi et al., 2021).

Even before the pandemic, scholars valued statistics of online behaviors concerning interests and mental health. A Canadian study of mental health queries between 2012 and 2017 showed that they fluctuate in a seasonal manner, with significant differences in summer and winter (Soreni et al., 2019).

Some scholars do not treat Google Trends as a good predictor of people’s mental wellbeing (Knipe et al., 2021). We believe that Wikipedia is better for such analyses, as people use this encyclopedia specifically to look for verified knowledge, rather than for general search purposes.

Wikipedia’s analysis has not been used for psychological queries, as we do in our paper. Its use in other fields is, however, well established: it was studied e.g. in the context of health information queries (Smith, 2020), analyzing if media coverage of some topics influences what people search for on Wikipedia (Gozzi et al., 2020), or comparing pandemic and pre-pandemic types of diet queries in Italy (Nucci et al., 2021). Long before the pandemic there were other studies of Wikipedia articles about changing popularity of the most trending topics in 2018 in three languages (Miz et al., 2020), neurological disorders (Brigo et al., 2015), seasonal disease trends in France (Vilain et al., 2017), health (Gabarron et al., 2015; Generous et al., 2014; Laurent & Vickers, 2009; Nuti et al., 2014), or measles infections in Italy tracked by Wikipedia traffic (Provenzano et al., 2019).

Given the number of articles about psychology in Wikipedia, the study is exploratory, and we refrain from formulating any hypotheses. We hope that our results will show the direction for further studies, perhaps using qualitative methods.

## Method

We used the *Index of psychology articles*, developed by WikiProject Psychology, a group of 290 volunteers with expertise in psychology.^1^ Between 2013 and 2019, they chose the topics for inclusion in a list of important Wikipedia articles about psychology. Although far from exhaustive, the list serves as a sample of general subjects within the field of psychology that non-specialists have consulted. We do not have any data on the volunteers’ credentials or background. We do know from other studies that the results of collaborative decision-making on Wikipedia are generally comparable to those of other professional sources, even in health-related topics (Smith, 2020), and that coverage of topics in psychology has been comprehensive for over a decade (Schweitzer, 2008). It is important to remember, however, that the criteria for inclusion for any of the articles overseen by the WikiProject Psychology result from discussion and decision of the volunteers.

## Procedure

The index we used includes 1763 articles from English Wikipedia. The complete list in alphabetical order by title is attached in Appendix 1. We listed these articles in PetScan, an internet tool enabling users to perform advanced analytics within Wikipedia. Our next step was to collect the total number of the articles’ daily views from July 1, 2015 and January 6, 2021 with the use of Massviews, a pageview analysis tool.

We compared the popularity of topics from this list on 10 large Wikipedias: English, German, French, Spanish, Polish, Portuguese, Russian, Swedish, Dutch, and Italian, which are among the most popular Wikipedias in the world.^2^ These Wikipedias also have the largest numbers of edits,^3^ which is a proxy for their community’s activity.

We used Python version 3.7.6 for statistical analyses and graphical representation of our data. To determine the magnitude of the increase or decrease in internet traffic to a specific Wikipedia article, we used the Wilcoxon’s test and set the α to 0.05. The Wilcoxon test is non-parametric, and there was no normal distribution in our data. To perform the test, we used Wilcoxon rank-sum statistic for two samples, a function in the SciPy library in Python, and calculated the effect size for it, according to the conventional equation for it: $r = Z / sqrt(n)$, using the rstatix R library (Tomczak & Tomczak, 2014).

For the sake of clarity, we present two types of graphs: the weekly or monthly views, and then standardize them for each Wikipedia entry. The rest of the graphs are in the *Supplementary files* section.

From the analyzed 1763 articles on English Wikipedia and the corresponding articles in nine other languages, we chose 50 with the largest increases in viewership and 50 with the largest decreases. We considered the pandemic period to start in March 2020 and continue to the end of our database (January 6, 2021) and we compared it with the pre-pandemic period, which started in July 1, 2015. We compared these results to the English analogues in the other nine languages. English Wikipedia is the largest and sometimes it happened that there were only a few or no analogues in other languages. That is why we start from analyzing English Wikipedia and then we look for the analogues—if we chose only the articles that are present in all ten languages, the whole picture would be obscured. We selected a few articles which we believe show the general trend of what happened on Wikipedia during the pandemic and use them to represent the most common topics from the psychological point of view of the internet users — stress, violence, education, employment, lockdowns, and relationships.

We decided that 100 articles from the total 1763 is a sufficient portion to draw conclusions and be able to create topic groups. We clustered the articles into topics iteratively, as we believed it made sense to see the bigger picture of changes during the pandemic. The categorization is not absolute, but we tried to achieve the level of objectivity in making it so that any other team working on this data would come up with a similar list of topics. All authors categorized the articles separately for reliability-check purposes. We treat this categorization as a part of subjective interpretation rather than a finding.

## Results

We present an analysis of the Wikipedia articles in psychology, chosen from the top 50 articles with the most increased viewership and 50 articles with the most decreased viewership (shown in Tables 1 and 2 in the supplementary files section), selected based on variations in the pattern in English and compared with nine languages. The tables also contain absolute sums of views before and during the pandemic, total sums of views, daily mean and standard deviation before and during the pandemic, W stat (the value of the Wilcoxon’s test), p value, effect size and effect magnitude for all languages.

The viewership for entries connected with psychology changed during the pandemic. For clarity reasons only, we treated some articles in some categories as representative, and did not discuss the other ones in the main body of the article. Still, such articles are discussed in detail in the supplement. Some articles became more popular, and we categorized them as follows:

- strictly pandemic related topics (“Social distance” as a representative article, “Comorbidity” and “Neurocognition” as supporting articles presented in the supplementary files section),
- methods to cope with stress (“Psychological pain,” “Defence mechanism,” “Substance intoxication”),
- non-physical violence and mistreatment (“Symbolic violence,” “Reactance (psychology)”).

Some other articles became less popular, and we categorized them as follows:

- purely scientific or academic psychological topics (“Sensation (psychology)” as a representative article, “Levels-of-processing effect” and “List of important publications in psychology” as supporting articles presented in the supplementary files section),
- topics connected with confinement (“Claustrophobia” as a representative article, “Stir crazy (condition)” as a supporting article described in the supplementary files section),
- family problems (“Relationship counseling”),
- stress and anxiety (“Stress (medicine)” as a representative article, “Introjection,” “Breathwork,” “Logotherapy” as supporting articles presented in the supplementary files section),
- jobs (“Burnout (psychology)”).

## Articles with increased viewing

**Fig. 1:**
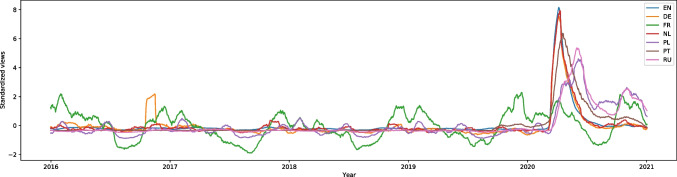
2016–2020 Standardized Monthly Sums of Views of “Social distance” Article in Seven Wikipedias

“Social distance” (from the category of strictly pandemic-related topics) on English Wikipedia had one of the highest increases of searches during the pandemic and had the W score equal to -25.15, effect size 0.56. In March 2020 there was a peak of around 25,000 searches a week, whereas the rest of the graph remained almost flat (the lowest number of weekly searches was around 200). This is shown in Fig. 12. In German, French, Dutch, Polish, Portuguese, and Russian the number of searches also rose during the pandemic (W scores equal to -13.70, -5.21, -15.36, -25.64, -26.96, and -27.38, effect size 0.31, 0.12, 0.35, 0.57, 0.81, and 0.61 respectively). Only French appears to have followed a specific trend each year; the article became more popular in the pandemic, but the number of searchers remained stable. All these languages are shown in Fig. 1. In other languages, we did not have any data.

**Fig. 2:**
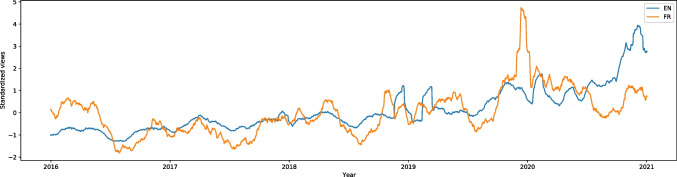
2016–2020 Standardized Monthly Sums of Views of “Psychological pain” article in two Wikipedias

“Psychological pain” (from the methods to cope with stress category) had a very significant increase of searches on English Wikipedia in our database (W = -24.75, effect size 0.55). Besides the significant rise compared to the previous years, there is a break in the trend during summer holidays of 2020. In each previous year the interest with this article continued to drop until the middle of August, but in 2020 it dropped between May and June, just to rise in June; it stayed at almost the same level until October, when it rose even more. A graph with only English Wikipedia is presented in Supplementary files in Fig. 19 to see each year more clearly. “Psychological pain” has a French counterpart among the languages we analyzed, and it also rose significantly (W = -11.70, effect size 0.26); the searches do not present any significant break in the trend from previous years, as can be seen in Figs. 2 and 20. There is an interesting peak between December 2019 and January 2020; there is less interest over the summer holidays than at other times.

**Fig. 3:**
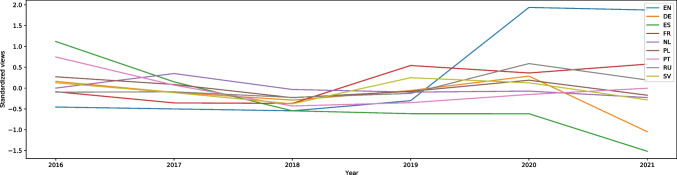
Standardized Annual Sums of Views of “Defence Mechanism” Article on Nine Wikipedias

“Defence mechanism” (also from the methods to cope with stress category) is the entry with one of the highest rises of interest among all the articles on English Wikipedia in our study (W = -26.11, effect size 0.58). It is difficult to analyze because the spectacular rise started in November 2019. There was around a 30-fold increase in the searches in comparison with previous years. This can be seen in Fig. 21. The article was present in all languages in our database. In Russian (W = -6.82, effect size 0.15) the searches peaked between April and May 2020 that corresponds to the more uniform graphs of the entries in English (at the highest point in 2020). In Portuguese and French the graphs look similar to the Russian one, but the W scores are even lower (Portuguese = -3.51, French = -2.53), meaning that it was searched more often during the pandemic than before; however, the effect size is very small – 0.08 in Portuguese and 0.06 in French despite being statistically significant. In German, Dutch, Polish and Swedish the results were not statistically significant (p = 0.37, p = 0.42, p = 0.24 and p = 0.59 respectively), indicating no significant difference between the number of searches during and before the pandemic. Only in Spanish the W score indicates a decrease in interest during the pandemic, and is equal to 6.83, effect size 0.15. Moreover, interest in the article in Spanish has decreased every year. All languages in our database are presented in Fig. 3. In Italian, there was no article.

**Fig. 4:**
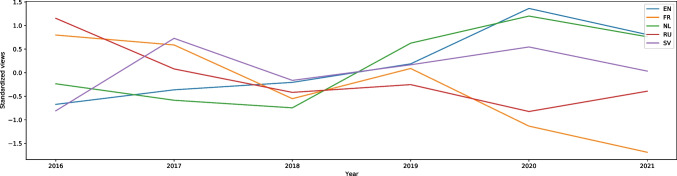
Standardized Annual Sums of Views of “Substance intoxication” Article in five Wikipedias

“Substance intoxication” (the third article from the methods to cope with stress category) also has the rise of searches on English Wikipedia during the pandemic, with the W score equal to -23.37, effect size 0.52. However, besides being significantly more popular in the pandemic, the graph, presented in Fig. 29, does not present a different trend from previous years; the rises and falls occur at around the same periods in all years. There is just an interesting peak in this article’s views at the turn of 2019 and 2020, with the highest number of weekly searches at 11,000, whereas the rest of 2020 has between 2,200 and 4,700 searches a week. In other languages in which we have the data, some increase during the pandemic: Dutch (W = -11.17, effect size 0.25) and Swedish (W = -3.37, effect size 0.08); and some decrease: German (W = 17.23, effect size 0.38) and Russian (W = 14.05, effect size 0.31). However, in these languages (even in the ones with an overall decrease) there are slight increases in mid-March 2020, even though in previous years the trends were different and nothing like that had previously happened in March (Fig. 4).

“Symbolic violence” (from the non-physical violence and mistreatment category) became more popular on English Wikipedia during the pandemic very significantly (the W score is equal to -23.64, effect size 0.53). As shown in Fig. 30, between 2016–2019 the graph is almost flat with between 55 and 190 searches a week. There are two drops each year — one between December and January, and the other from May to mid-July, and then rising until its highest point in November. In 2020 the behavior is similar, except that there are three to four times more searches than between 2016 and 2019.

**Fig. 5:**
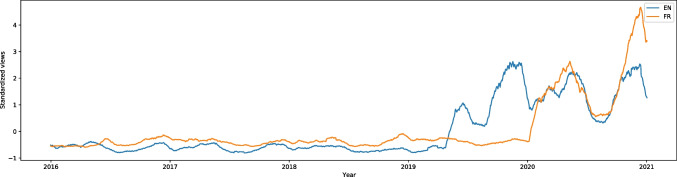
2016–2021 Standardized Monthly Sums of Views of “Symbolic Violence” Article in two Wikipedias

The second problem is that in 2019 we have a slight break from the trend by a twofold increase in interest in May (again gently decreasing over the summer holidays), and an almost fourfold increase in the fall. In 2020 the weekly interest stayed at around 600 searches with minor fluctuations, with an interesting peak in June (770 searches a week), just before the usual drop in the summer holidays to the lowest point of 260 weekly searches in August; later, the interest behaves like in previous years, growing to the highest point in November to 890 searches a week. In French, it has the W score equal to -27.43, effect size 0.61, which means it also increased, and the graph looks in most parts the same as the English one (additionally, the number of visits are almost the same in both languages). The difference appears in 2019 — in French the interest did not increase, however at the beginning of 2020 it is almost the same; only the second peak is higher in French (to around 850 searches a week). The trends can be seen in Figs. 5 and 31.

**Fig. 6:**
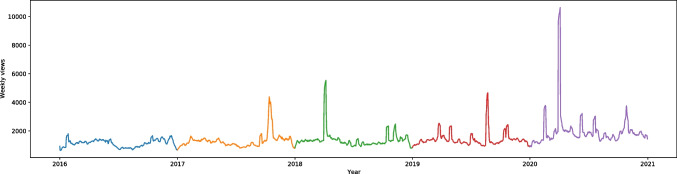
2016–2021 Weekly Views of “Reactance (psychology)” Article in English Wikipedia

“Reactance (psychology)” (also from the non-physical violence and mistreatment category) had an increase of interest on English Wikipedia, with the W = -20.28, effect size 0.45. As shown in Fig. 6, the highest peak was at the end of March 2020, with more than 10,000 searches a week. The rest of the graph in 2020 remained almost flat, with a few peaks (to around 4000 searches weekly) in the middle of February, beginning of June, mid-July, and at the end of October. The rest of the pandemic period had the lowest values of around 1300 weekly searches. This entry is present in German, Spanish, French, Polish, Portuguese, and Russian; in all of them viewership rose significantly (their W scores are -14.73, -4.66, -22.01, -9.39, -9.41, and -12.60, effect size 0.33, 0.10, 0.49, 0.21, 0.21, and 0.28 respectively). What is even more interesting for our context, the peak from the end of March 2020 does not seem to follow any trend from previous years, but it is present at around the same spot in all languages in our database (and it does not follow any trend from previous years). This change in the trend can be seen in Fig. 32. The peaks in other languages are not as spectacular as in English, but still, they break the trend from previous years.

## Articles with decreased viewing

**Fig. 7:**
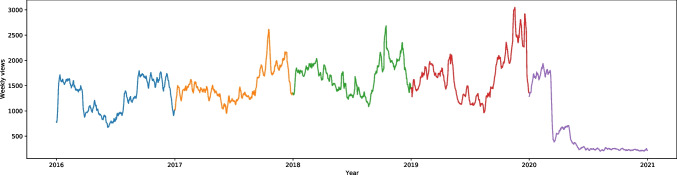
2016–2021 Weekly Views of “Sensation (psychology)” Article in English Wikipedia

There is an interesting group of purely scientific or academic psychological topics, with a considerable drop at the beginning of the pandemic. They follow trends between 2016–2020, but there is an unprecedented plunge in interest in March 2020. We will present this situation using the topic “Sensation (psychology)*”* (W = 27.17, effect size 0.60) with Fig. 7. It presents a consistent trend of high interest at the beginning of 2020 and even higher after the summer holidays. The drop occurs between June and October where there are half as many views than at the beginning of the year and one-third the number of views than after October. The year 2020 begins just like previous years, but in March the number of views falls and there are one-eighth the number of views than previously, and this figure stays the same until the end of the year. Other graphs are presented in the Supplementary Files section because the situation looks almost the same as with “Sensation (psychology)”; these are”Levels-of-processing effect” (W = 28.03, effect size 0.62) in Fig. 34 in the supplementary files and “List of important publications in psychology” (W = 26.03, effect size 0.58) in Fig. 35. These articles appeared only in English Wikipedia.

**Fig. 8:**
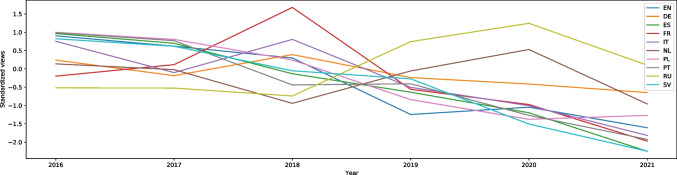
Standardized Annual Sums of Views of “Claustrophobia” Article in 10 Wikipedias

In the topics connected with confinement category, we focus on “Claustrophobia” rather than “Stir crazy (condition).” We were led to search for “Claustrophobia” article, which is not in the top 50 searches, after reading the description of “Stir crazy (condition)” and deciding it is closely connected and referring to the same phenomenon, and with “Claustrophobia” being a more common term to describe the condition. The article “Claustrophobia” in English Wikipedia has two significant peaks at the beginning of June and at the end of August (Fig. 39). Overall, the entry had a very high W score in our test (19.19), effect size 0.43, indicating a decline in searches. “Stir crazy (condition)” has a similar change in viewership, but we present its description and graphs in the supplementary files section in Figs. 36, 37 and 38. “Claustrophobia” is present in all languages that we discuss in this paper. In German, Spanish, French, Italian, Polish, Portuguese, and Swedish the searches decreased as significantly as in English, with the W scores equal to 18.40, 19.61, 17.77, 13.38, 20.90, 23.95, and 22.17, and effect size 0.41, 0.44, 0.40, 0.30, 0.47, 0.53, and 0.49 respectively. There are similar bumps in the graphs in March 2020 as well as at the beginning of June and around the end of the summer vacation. Only in Dutch (W = -3.54, effect size 0.08) and Russian (W = -18.35, effect size 0.41) the interest in “Claustrophobia” increased. This is shown in Fig. 8.

**Fig. 9:**
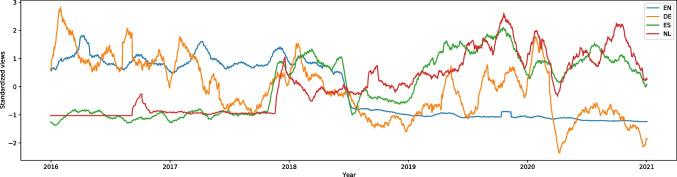
2016–2021 Standardized Monthly Sums of Views of “Relationship counseling” Article in four Wikipedias

“Relationship counseling” (from the family problems category) decreased in the English (W = 26.52, effect size 0.59) and German Wikipedias (W = 16.56, effect size 0.37). In English Wikipedia, interest in this topic began to wane in mid- 2018, from around 2000 to 500 searches a week. It decreased to 50 searches a week by the end of 2020 (Fig. 41). The pandemic did not have any impact. In German Wikipedia, interest in the topic not only decreased overall from previous years, but also had a significant dip in mid-March 2020. After the dip, it did not return to the level at the beginning of the year (400–600 searches a week), but stayed at around 300 searches a week. In Spanish Wikipedia (W = -14.15, effect size 0.32) and in Dutch Wikipedia (W = -16.60, effect size 0.38) the number of searches for this topic increased. The interest grew in 2019 and fluctuated between 400 and 900, just like in 2020. In 2020, however, interest was only bigger in January, the pre-pandemic period (50 weekly searches more) and briefly in July (100 weekly searches more). In other months it is lower and has slighter increases and decreases than in 2019. It is also worth noting that in 2016 and 2017 the interest was at a level of around 200 weekly searches, which influenced the W score of our test, but the increase in later years does not seem to have been caused by the pandemic (there is no visible or rapid break with the trend from previous years after March 2020). In Dutch Wikipedia, the article was not searched at all until September 2016. In 2017, it had just a few visits per week. The most interest is from the beginning of 2020 until March, and at the end of September in 2019 and 2020 (there are the same peaks in both years). In the remaining months of 2019 and 2020, the number of visits is similar; the pandemic seems to have had no effect. Figures 9, 40, and 41 show the results from four Wikipedias.

**Fig. 10:**
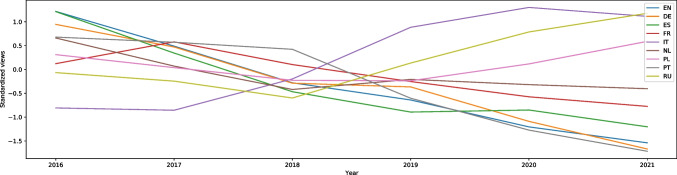
Standardized Annual Sums of Views of “Stress (medicine)” Article in Nine Wikipedias

“Stress (medicine)” (from the stress and anxiety category) decreased significantly after March 2020, with a W score equal to 25.98, effect size 0.58. However, there has been a decrease since 2017, as shown in Fig. 42. Similar decreases in interest are found in German (W = 16.93, effect size 0.38), Spanish (W = 15.15, effect size 0.34), French (W = 20.67, effect size 0.46), Dutch (W = 6.24, effect size 0.14), and Portuguese (W = 22.92, effect size 0.51). All of the graphs show slightly fewer views during the summer holidays, and a very small increase after it until the next summer. In Polish, the difference in the number of searches between the pandemic and previous years is not statistically significant (p = 0.85), which means it is almost the same as before. There are only two languages in which the views of “Stress (medicine)” increased: Italian and Russian. In Italian there was a significant increase in the interest with a W score equal to -22.04, effect size 0.49. In March and April 2019 there was a decrease of searches (around 700 searches a week), whereas in the same months in 2020 there was an increase of searches (around 1400 searches a week). Besides this difference, the rest of the graph from 2020 follows the trend of the one from 2019 and 2018. It is also important to note that interest in the article in Italian was nonexistent until September 2018; previously it had around 100 searches a week. The situation was similar with Russian. Overall interest increased (the W score is equal to -10.18, effect size 0.23), but there are more searches only at the beginning of 2020 in comparison to previous years (9000 at the highest point in the middle of May 2020, and 6000 searches a week in the same period in 2019, 2017, and 2016; 2018 was an outlier). After that, each year repeats the trend, decreasing for the summer holidays and increasing after it. Figure 10 shows the trend in nine languages.

**Fig. 11:**
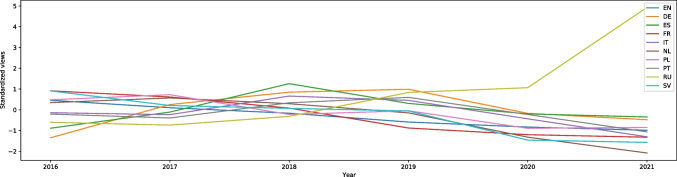
Standardized Annual Sums of Views of “Burnout (psychology)” Article in 10 Wikipedias

“Burnout (psychology)” (from the jobs category) is another topic that showed a decrease in interest. In English, the W score is equal to 27.11, effect size 0.60. Figure 43 shows the trend since the beginning of the period in our data. It is present in all other languages, and the interest also decreased: in German (W = 6.34, effect size 0.14), French (W = 26.32, effect size 0.59), Italian (W = 13.08, effect size 0.29), Dutch (W = 24.36, effect size 0.54), Polish (W = 16.78, effect size 0.37), Portuguese (W = 5.35, effect size 0.12), and Swedish (W = 22.97, effect size 0.51). In Spanish there is an increase in viewership (W = -2.17, effect size 0.05), but the W score is influenced by very low traffic from 2016 to August 2017, when the number of searches fluctuated between 10–40 searches a week, followed by a sudden leap to 6000. In 2020 there were between 600 and 1800 searches a week. However, all the interest in all these languages behave similarly on the graphs — there was a small decline in the middle of March 2020, followed by a gradual decrease until September. After that, there was a marginal increase in interest. In Russian (W = -19.07, effect size 0.42) there was an increased number of searches during the pandemic. However, the interest in 2020 was not much different from 2019. We can see a rise starting in April, but to the same level as in 2019 (400 searches a week). The peak from September to December is interesting (to 8000 searches a week) because in previous years the interest was decreasing. Figure 11 shows the trends in all languages.

## Discussion

The pandemic changed the way people interact, work, and live. People turned to Wikipedia for information on topics that were connected with the pandemic. When we take a closer look at these topics, “Social distance” is among the 10 topics that was most frequently researched during the pandemic, because almost everywhere in the world a “social distance” of at least two meters was recommended to reduce the chance of infection (Koh, 2020). Another article that became more popular was “Comorbidity,” described in the supplementary files section in Figs. 14 and 15. It was an important factor that was connected with mortality caused by COVID-19. Another interesting behavior is the significant drop during the summer holidays, which were the months of decreased mortality caused by COVID-19 (WHO Coronavirus (COVID-19) Dashboard, n.d.-a). It seems that peaks correspond to the waves of COVID-19. With the increasing understanding of COVID-19 with time, it became obvious that the virus caused neurologic and neuropsychiatric complications (Ellul et al., 2020). On Wikipedia more people searched the article “Neurocognition*,*” described in the supplementary files section in Figs. 16, 17, and 18.

We also studied articles on stress management. The pandemic was a very stressful time in terms of health and well-being, social relations, lockdown, and job insecurity (CDC, 2021). Two explanations for the increased number of searches for the article “Psychological pain,” could be that people were looking for the scientific description of the problems they experienced during the pandemic, or that they wanted to help others. One way to cope with stress and psychological pain could be the use of defence mechanisms (Gori et al., 2020). The article “Defence mechanism” had more views than before the pandemic. It cannot be explained by a momentary interest with this topic by the media because throughout 2020 it stayed much higher than in previous years. The reasons for the increased interest with defence mechanisms can be backed by another article, which became more popular during the pandemic: “Introjection*,*” described in the supplementary files in Figs. 22, 23, and 24. It is categorized on Wikipedia as a defence mechanism and defines behavior of “a person who picks up traits from his or her friends” (Wikipedia contributors, 2020). We believe that these two articles being viewed more during the pandemic can mean that people wanted to understand their reactions (or those of people around them) or to understand what happens around them better.

Two other articles also became more popular in the pandemic. One was “Breathwork” (described in the supplementary files section in Figs. 25 and 26), a concept in alternative medicine, which is supposed to manage stress by conscious control of breathing; and the other was “Logotherapy” (described in the supplementary files section in Figs. 27 and 28), a form of therapy that helps people find the meaning of life. Indeed, people look for the meaning in life when they encounter death (Routledge & Juhl, 2010).

The last article which we included in this group is “Substance intoxication.” We put it in this group because although it is not a productive way to handle stress, people abuse substances, and perhaps even more so during the pandemic (Clay & Parker, 2020; dos Santos et al., 2021). However, it is difficult to say why people visited this article since it is nothing new, and most people know not to use alcohol or drugs to excess. It may be possible that because of more people using intoxicating substances, more people are searching for ways to prevent it (Eysenbach & Diepgen, 1999).

During the pandemic, many jobs became remote. Schools and universities turned to online education, which created new possibilities for bullying (Mahmoudi & Keashly, 2020). This could be connected to our third topic — non-physical violence and mistreatment. The first Wikipedia entry we listed here is “Symbolic violence.” It is defined as.

a type of non-physical violence manifested in the power differential between social groups. It is often unconsciously agreed upon by both parties and is manifested in the imposition of the norms of the group possessing greater social power on those of the subordinate group (Wikipedia contributors, 2021a).

Although the viewership of “Symbolic violence” rose quite significantly, it is difficult to analyze this entry in our context because of two issues. The first problem is that between 2016–2019 the graph is almost flat with searches between 55 and 190 a week. The second is that the interest is indeed much higher than in previous years, but, for some reason, it started to build in May 2019.

It is interesting to look at this result at the same time with another article: “Reactance (psychology).” It is defined as a reaction to someone or something that limits people’s freedom of choice. In this context, we could interpret the article “Symbolic violence” to mean that governments possess more social power than people. During the pandemic at a point when vaccines became available, anti-vaccination views proliferated (Jemielniak & Krempovych, 2021; Johnson et al., 2020). These views were often linked to conspiracy theories about secret organizations imposing an evil plan on the population (Ball & Maxmen, 2020). Another explanation of more views of “Reactance (psychology)” could be that readers believed that their freedom was being limited by the lockdowns and the mask mandates. There was also a similar article to prove that Reactance was not viewed more often by accident — “Righteous indignation” (presented and described in the supplementary files section in Fig. 33), described on Wikipedia as a “reactive emotion of anger over mistreatment, insult, or malice of another.”

After analyzing these three groups of topics that became more popular in the pandemic, it is interesting to see the ones which became less popular. Some could be connected to the deterioration of education due to the lockdown and online studying (Dorn et al., 2020). Among the least-searched topics are “Sensation (psychology),” “Levels-of-processing effect,” and “List of important publications in psychology.” These are rather scientific, technical subjects. One of the possible explanations could be that students did not study as hard as before the pandemic, as evidenced by the increased incidence of cheating among students in spring 2020 (Bilen & Matros, 2021). Students who cheat on exams do not need extensive knowledge of the topic, just the answers to the questions, which could be why these topics were less viewed.

It is difficult to understand why some topics became less popular during the pandemic. These are “Claustrophobia,” “Stir crazy (condition),” “Relationship counseling,” or “Stress (medicine).” “Stir crazy (condition)” is described as “distressing claustrophobic irritability or restlessness experienced when a person, or a group, is stuck at an isolated location or in confined quarters for an extended time” (Wikipedia contributors, 2021b). These articles should have been more popular.

“Relationship counseling” is another interesting topic to analyze. It is commonly believed that the pandemic and lockdowns increased the number of divorces and break-ups (Savage, 2020). If there are problems which can lead to a divorce, it would seem logical that there will be an increased interest in “Relationship counseling.” The number of searches decreased in English and in German, and increased in Spanish and Dutch (but the article in these two languages was very rarely visited before 2018). Perhaps before the pandemic, couples who had problems were already using professional help or consulting online sources that were not Wikipedia, but during the lockdowns the problems were too much to overcome. However, the number of views of the article on Wikipedia does not have to mean that people do not go to therapy, but people do search the internet for medical information, which makes our result even more perplexing.

Another article which was almost forgotten during the pandemic is “Stress (medicine).” The pandemic seems to be a perfect reason for a peak of interest, but nothing like that happened. Articles about stress management techniques were more popular during the pandemic (“Introjection,” “Breathwork,” “Logotherapy,” presented and described in Figs. 22–27 in the supplementary files), but the article about stress itself was not (only in Italian and Russian it was viewed more). It is possible that people were already familiar with stress and did not need to read about it in Wikipedia, but the reason for it being searched before the pandemic but not as much after March 2020 is puzzling. The situation of Italy is interesting, as at the beginning of the pandemic it was the second country after China with the most severe impact and death toll due to COVID-19 (Cesari & Proietti, 2020), which may be the reason why in Italian there is an increase of searches. In Russian the increase may be explained by the severity of the COVID-19 pandemic. Russia was the fourth country with the highest death toll (WHO Coronavirus (COVID-19) Dashboard, n.d.-b), but some researchers claim that the statistics were manipulated by the government (Dyer, 2020, 2021).

The last article that we listed was connected with jobs — it was “Burnout (psychology).” There was a decline in interest in this article in all languages except for Spanish and Russian. Several studies have linked job insecurity to burnout (Blom et al., 2018). During the pandemic there was obvious job insecurity in many employment sectors, mostly in the service industry, and yet, the Wikipedia article concerning burnout dropped significantly; it was the seventh-greatest decline in our database. It would be wrong to assume that people who were working during the pandemic did not experience burnout, or that the pandemic *cured* the burnout problem. Of course, it is not possible to conclude that people do not experience burnout because they do not read Wikipedia, but people indeed seek medical information online, so it would be very interesting to see if the trend of visits to that article returns to the previous level when the COVID-19 pandemic comes to an end.

## Strengths and weaknesses

One of the biggest strengths of our paper is that it is the first study that analyzes psychological themes in a multicultural context of 10 languages at the same time, based on a very reliable and well-developed source: Wikipedia. The problem with Wikipedia is that we do not know anything about the users, we do not know where they go next or if they read about the problem in the article in some other sources, and ultimately we do not know the purpose of the visit on the Wikipedia page; as stated in the method section, that the inclusion of a psychological article is made by volunteers. Still, it gives us an interesting picture to draw conclusions about online behavior. It would be interesting to see even a wider picture with the analysis of Google Trends or Reddit searches. Another possible weakness would be the use of mostly European languages, almost all based on the Latin alphabet—it would be interesting to see how users behave in non-European languages.

## Conclusions

Our results show that the new social context resulted in several new search patterns, some of which are somewhat surprising. Clearly, new ways of coping with stress have been sought after. For instance, the “traditional” article on “Stress (medicine)” experienced a drop in searches in most languages. Even more interestingly, so did the articles on couples therapy ("Relationship counseling”) or confinement ("Claustrophobia”, “Stir crazy”), which one could expect to rise in popularity. At the same time, the popularity of “Breathwork,” a stress-coping technique of unproven efficacy, increased. An overall theme could be an increase in searches for self-help topics, and a decrease in searches for professional help, often requiring some form of in-person contact.

The increased popularity of “Substance intoxication” is somewhat understandable, and may signal a trend of using alcohol as a stress reducer.

One important result that we observe is a collapse of education: many academic topics experienced significant drops in traffic, which we can only explain through decreased student activity. Students use Wikipedia to learn, as well as to cheat on exams and essays. In theory, given the modality of remote examining, we could expect a growth in Wikipedia traffic due to cheating. Nevertheless, even if such growth happened, it was nullified by an overall drop in reading for educational purposes.

## Supplementary Information

Below is the link to the electronic supplementary material.

Supplementary file1 (DOCX 1481 KB)

## Footnotes

1 The list of volunteers can be checked here: https://en.wikipedia.org/wiki/Wikipedia:WikiProject_Psychology#Participants

2 Per http://wikistics.org/pages/ counting main and mobile landing page.

3 https://meta.wikimedia.org/wiki/List_of_Wikipedias#1_000_000+_articles We use the top 10 Wikipedias, but we excluded Cebuano, as even though Cebuano Wikipedia is large, it relies on automated translations and bot edits. Instead, we added Portuguese, which is the next in line language based on the Latin alphabet, and a language of which we have some understanding.
