# Supplementary material for: What’s hot and what's not in lay psychology: Wikipedia’s most-viewed articles
Source: Curr Psychol. 2022 Oct 12:1–13. Online ahead of print. doi: 10.1007/s12144-022-03826-0 (PMC9553632; doi:10.1007/s12144-022-03826-0)
Supplement: Supplementary file 1 — Supplementary file1 (DOCX 1481 KB) [file 12144_2022_3826_MOESM1_ESM.docx]

**Supplementary files**

**Table 1**

*Top 50 Articles in English and their Equivalents with the Largest Increase in Viewership*

|  | **Page name** | **Language** | **Absolute sum before pandemic** | **Absolute sum pandemic** | **Absolute total sums** | **Daily mean before pandemic** | **Daily std before pandemic** | **Daily mean during pandemic** | **Daily std during pandemic** | **W_stat** | **p_value** | **effect_size** | **effect_magnitude** |
| --- | --- | --- | --- | --- | --- | --- | --- | --- | --- | --- | --- | --- | --- |
| **1** | Neurocognition | EN | 1,677 | 8,520 | 10,197 | 0.98 | 2.12 | 27.31 | 11.94 | -27.87 | 0.00 | 0.66 | large |
|  | Neurocognition | DE | 15,561 | 2,064 | 17,625 | 9.13 | 6.91 | 6.62 | 3.28 | 8.82 | 0.00 | 0.20 | small |
| **2** | Masking (personality) | EN | 141,864 | 70,459 | 212,323 | 83.20 | 33.65 | 225.83 | 82.00 | -27.73 | 0.00 | 0.62 | large |
|  | Masking (personality) | PT | 0 | 0 | 0 | 0.00 | 0.00 | 0.00 | 0.00 | 0.00 | 1.00 |  |  |
| **3** | Defence mechanism | EN | 236,230 | 298,851 | 535,081 | 138.55 | 237.36 | 957.86 | 201.74 | -26.11 | 0.00 | 0.58 | large |
|  | Defence mechanism | DE | 455,731 | 86,559 | 542,290 | 267.29 | 59.20 | 277.43 | 114.77 | -0.89 | 0.37 | 0.02 | small |
|  | Defence mechanism | ES | 1,062,745 | 155,791 | 1,218,536 | 623.31 | 273.27 | 499.33 | 158.44 | 6.83 | 0.00 | 0.15 | small |
|  | Defence mechanism | FR | 229,702 | 44,189 | 273,891 | 134.72 | 47.60 | 141.63 | 43.85 | -2.53 | 0.01 | 0.06 | small |
|  | Defence mechanism | IT | 0 | 0 | 0 | 0.00 | 0.00 | 0.00 | 0.00 | 0.00 | 1.00 |  |  |
|  | Defence mechanism | NL | 97,217 | 17,174 | 114,391 | 57.02 | 99.57 | 55.04 | 19.92 | -0.81 | 0.42 | 0.02 | small |
|  | Defence mechanism | PL | 280,660 | 51,838 | 332,498 | 164.61 | 59.50 | 166.15 | 55.73 | -1.18 | 0.24 | 0.03 | small |
|  | Defence mechanism | PT | 291,794 | 56,576 | 348,370 | 171.14 | 85.39 | 181.33 | 72.41 | -3.51 | 0.00 | 0.08 | small |
|  | Defence mechanism | RU | 470,659 | 151,765 | 622,424 | 276.05 | 79.65 | 486.43 | 3075.80 | -6.82 | 0.00 | 0.15 | small |
|  | Defence mechanism | SV | 214,092 | 40,407 | 254,499 | 125.57 | 55.52 | 129.51 | 61.25 | -0.54 | 0.59 | 0.01 | small |
| **4** | Womb envy | EN | 19,807 | 18,714 | 38,521 | 11.62 | 15.26 | 59.98 | 23.51 | -26.14 | 0.00 | 0.58 | large |
|  | Womb envy | PT | 14,099 | 1,720 | 15,819 | 8.27 | 6.33 | 5.51 | 3.44 | 6.90 | 0.00 | 0.15 | small |
| **5** | Host (psychology) | EN | 16,997 | 12,712 | 29,709 | 9.97 | 10.19 | 40.74 | 14.52 | -26.48 | 0.00 | 0.59 | large |
| **6** | Symbolic violence | EN | 42,494 | 23,570 | 66,064 | 24.92 | 25.55 | 75.54 | 30.98 | -23.64 | 0.00 | 0.53 | large |
|  | Symbolic violence | FR | 11,982 | 16,991 | 28,973 | 7.03 | 7.48 | 54.46 | 28.89 | -27.43 | 0.00 | 0.61 | large |
|  | Symbolic violence | PT | 4 | 0 | 4 | 0.00 | 0.10 | 0.00 | 0.00 | 0.02 | 0.99 | 0.01 | small |
| **7** | Neurosyphilis | EN | 983,458 | 459,767 | 1,443,225 | 576.81 | 188.75 | 1473.61 | 1075.54 | -25.42 | 0.00 | 0.57 | large |
|  | Neurosyphilis | ES | 126,183 | 26,689 | 152,872 | 74.01 | 70.36 | 85.54 | 37.27 | -7.40 | 0.00 | 0.16 | small |
|  | Neurosyphilis | FR | 94,352 | 38,250 | 132,602 | 55.34 | 20.42 | 122.60 | 82.03 | -25.13 | 0.00 | 0.56 | large |
|  | Neurosyphilis | PL | 5,956 | 1,850 | 7,806 | 3.49 | 2.66 | 5.93 | 6.14 | -8.07 | 0.00 | 0.18 | small |
|  | Neurosyphilis | RU | 138,421 | 85,316 | 223,737 | 81.19 | 123.85 | 273.45 | 250.29 | -23.45 | 0.00 | 0.52 | large |
| **8** | Substance intoxication | EN | 372,173 | 120,747 | 492,920 | 218.28 | 174.62 | 387.01 | 92.79 | -23.37 | 0.00 | 0.52 | large |
|  | Substance intoxication | DE | 129,124 | 18,220 | 147,344 | 75.73 | 17.95 | 58.40 | 11.40 | 17.23 | 0.00 | 0.38 | moderate |
|  | Substance intoxication | NL | 19,199 | 4,529 | 23,728 | 11.26 | 4.62 | 14.52 | 4.81 | -11.17 | 0.00 | 0.25 | small |
|  | Substance intoxication | RU | 67,522 | 9,539 | 77,061 | 39.60 | 14.14 | 30.57 | 8.43 | 14.05 | 0.00 | 0.31 | moderate |
|  | Substance intoxication | SV | 13,561 | 2,689 | 16,250 | 7.95 | 4.37 | 8.62 | 4.35 | -3.37 | 0.00 | 0.08 | small |
| **9** | Social distance | EN | 122,164 | 145,415 | 267,579 | 71.65 | 24.58 | 466.07 | 747.69 | -25.15 | 0.00 | 0.56 | large |
|  | Social distance | DE | 43,804 | 16,245 | 60,049 | 25.69 | 19.42 | 52.07 | 56.37 | -13.70 | 0.00 | 0.31 | moderate |
|  | Social distance | FR | 41,740 | 8,709 | 50,449 | 24.48 | 10.65 | 27.91 | 11.36 | -5.21 | 0.00 | 0.12 | small |
|  | Social distance | IT | 0 | 0 | 0 | 0.00 | 0.00 | 0.00 | 0.00 | 0.00 | 1.00 |  |  |
|  | Social distance | NL | 2,202 | 2,055 | 4,257 | 1.29 | 1.72 | 6.59 | 11.79 | -15.36 | 0.00 | 0.35 | moderate |
|  | Social distance | PL | 12,451 | 9,376 | 21,827 | 7.30 | 5.03 | 30.05 | 19.49 | -25.64 | 0.00 | 0.57 | large |
|  | Social distance | PT | 650 | 6,695 | 7,345 | 0.38 | 1.44 | 21.46 | 23.44 | -26.96 | 0.00 | 0.81 | large |
|  | Social distance | RU | 13,179 | 43,768 | 56,947 | 7.73 | 5.32 | 140.28 | 88.79 | -27.38 | 0.00 | 0.61 | large |
| **10** | Psychological pain | EN | 376,496 | 116,285 | 492,781 | 220.82 | 80.16 | 372.71 | 111.91 | -24.75 | 0.00 | 0.55 | large |
|  | Psychological pain | FR | 32,998 | 7,684 | 40,682 | 19.35 | 9.44 | 24.63 | 7.64 | -11.70 | 0.00 | 0.26 | small |
| **11** | Comorbidity | EN | 1,412,582 | 558,753 | 1,971,335 | 828.49 | 221.85 | 1790.88 | 820.66 | -24.95 | 0.00 | 0.56 | large |
|  | Comorbidity | DE | 599,692 | 73,370 | 673,062 | 351.73 | 80.88 | 235.16 | 48.70 | 22.56 | 0.00 | 0.50 | large |
|  | Comorbidity | ES | 522,357 | 324,734 | 847,091 | 306.37 | 86.24 | 1040.81 | 536.45 | -27.31 | 0.00 | 0.61 | large |
|  | Comorbidity | FR | 205,304 | 159,019 | 364,323 | 120.41 | 52.23 | 509.68 | 762.08 | -23.21 | 0.00 | 0.52 | large |
|  | Comorbidity | IT | 187,733 | 50,050 | 237,783 | 110.11 | 33.14 | 160.42 | 123.43 | -10.23 | 0.00 | 0.23 | small |
|  | Comorbidity | NL | 129,463 | 19,335 | 148,798 | 75.93 | 35.23 | 61.97 | 25.00 | 7.12 | 0.00 | 0.16 | small |
|  | Comorbidity | PT | 124,798 | 101,469 | 226,267 | 73.20 | 43.77 | 325.22 | 255.14 | -25.58 | 0.00 | 0.57 | large |
|  | Comorbidity | RU | 203,598 | 43,001 | 246,599 | 119.41 | 34.69 | 137.82 | 43.87 | -6.14 | 0.00 | 0.14 | small |
|  | Comorbidity | SV | 44,496 | 7,483 | 51,979 | 26.10 | 12.15 | 23.98 | 74.83 | 9.46 | 0.00 | 0.21 | small |
| **12** | Sylvia Plath effect | EN | 135,295 | 72,074 | 207,369 | 79.35 | 177.50 | 231.01 | 567.66 | -24.61 | 0.00 | 0.55 | large |
|  | Sylvia Plath effect | PL | 6,253 | 1,066 | 7,319 | 3.67 | 2.44 | 3.42 | 2.35 | 1.73 | 0.08 | 0.04 | small |
|  | Sylvia Plath effect | PT | 4,150 | 526 | 4,676 | 2.43 | 3.54 | 1.69 | 1.49 | 5.32 | 0.00 | 0.12 | small |
|  | Sylvia Plath effect | RU | 20,568 | 2,159 | 22,727 | 12.06 | 9.38 | 6.92 | 4.75 | 13.86 | 0.00 | 0.31 | moderate |
| **13** | Introjection | EN | 217,732 | 58,372 | 276,104 | 127.70 | 34.07 | 187.09 | 25.27 | -23.17 | 0.00 | 0.52 | large |
|  | Introjection | DE | 116,773 | 25,558 | 142,331 | 68.49 | 16.49 | 81.92 | 16.87 | -12.64 | 0.00 | 0.28 | small |
|  | Introjection | ES | 185,334 | 25,731 | 211,065 | 108.70 | 38.40 | 82.47 | 26.01 | 11.77 | 0.00 | 0.26 | small |
|  | Introjection | FR | 47,565 | 9,526 | 57,091 | 27.90 | 11.20 | 30.53 | 10.60 | -3.85 | 0.00 | 0.09 | small |
|  | Introjection | IT | 23,489 | 4,680 | 28,169 | 13.78 | 5.34 | 15.00 | 11.08 | -1.27 | 0.20 | 0.03 | small |
|  | Introjection | NL | 7,864 | 1,288 | 9,152 | 4.61 | 4.09 | 4.13 | 3.06 | 2.77 | 0.01 | 0.06 | small |
|  | Introjection | PL | 43,036 | 7,439 | 50,475 | 25.24 | 11.28 | 23.84 | 8.47 | 1.68 | 0.09 | 0.04 | small |
|  | Introjection | PT | 22,106 | 4,228 | 26,334 | 12.97 | 6.64 | 13.55 | 7.33 | -1.15 | 0.25 | 0.03 | small |
|  | Introjection | RU | 119,727 | 31,585 | 151,312 | 70.22 | 22.66 | 101.23 | 19.10 | -20.99 | 0.00 | 0.47 | moderate |
| **14** | Breathwork | EN | 214,154 | 72,594 | 286,748 | 125.60 | 65.29 | 232.67 | 68.43 | -23.48 | 0.00 | 0.52 | large |
|  | Breathwork | DE | 3,719 | 1,767 | 5,486 | 2.18 | 2.35 | 5.66 | 4.18 | -18.80 | 0.00 | 0.42 | moderate |
| **15** | Logorrhea (psychology) | EN | 423,100 | 136,294 | 559,394 | 248.15 | 347.93 | 436.84 | 166.38 | -24.12 | 0.00 | 0.54 | large |
|  | Logorrhea (psychology) | DE | 382,739 | 60,930 | 443,669 | 224.48 | 197.74 | 195.29 | 78.75 | 5.90 | 0.00 | 0.13 | small |
|  | Logorrhea (psychology) | ES | 119,987 | 20,263 | 140,250 | 70.37 | 48.15 | 64.95 | 20.83 | 4.11 | 0.00 | 0.09 | small |
|  | Logorrhea (psychology) | FR | 241,752 | 49,421 | 291,173 | 141.79 | 114.65 | 158.40 | 42.77 | -9.01 | 0.00 | 0.20 | small |
|  | Logorrhea (psychology) | IT | 79,726 | 20,092 | 99,818 | 46.76 | 15.40 | 64.40 | 36.37 | -15.43 | 0.00 | 0.34 | moderate |
|  | Logorrhea (psychology) | NL | 15,960 | 2,579 | 18,539 | 9.36 | 15.92 | 8.27 | 6.91 | 2.21 | 0.03 | 0.05 | small |
|  | Logorrhea (psychology) | PL | 61,370 | 11,595 | 72,965 | 35.99 | 14.81 | 37.16 | 13.88 | -1.79 | 0.07 | 0.04 | small |
|  | Logorrhea (psychology) | PT | 36,383 | 9,714 | 46,097 | 21.34 | 9.24 | 31.13 | 11.35 | -15.86 | 0.00 | 0.35 | moderate |
|  | Logorrhea (psychology) | RU | 80,132 | 14,981 | 95,113 | 47.00 | 18.31 | 48.02 | 27.60 | -0.13 | 0.90 | 0.00 | small |
|  | Logorrhea (psychology) | SV | 14 | 513 | 527 | 0.01 | 0.21 | 1.64 | 1.72 | -19.93 | 0.00 | 0.81 | large |
| **16** | Individual psychology | EN | 179,185 | 49,431 | 228,616 | 105.09 | 31.72 | 158.43 | 29.05 | -22.22 | 0.00 | 0.49 | moderate |
|  | Individual psychology | DE | 137,677 | 37,625 | 175,302 | 80.75 | 45.13 | 120.59 | 22.27 | -21.75 | 0.00 | 0.48 | moderate |
|  | Individual psychology | ES | 168,234 | 38,124 | 206,358 | 98.67 | 45.83 | 122.19 | 46.95 | -8.31 | 0.00 | 0.19 | small |
|  | Individual psychology | FR | 29,251 | 6,013 | 35,264 | 17.16 | 34.02 | 19.27 | 8.44 | -7.21 | 0.00 | 0.16 | small |
|  | Individual psychology | IT | 26,298 | 6,452 | 32,750 | 15.42 | 6.74 | 20.68 | 7.73 | -11.04 | 0.00 | 0.25 | small |
|  | Individual psychology | NL | 5,531 | 1,040 | 6,571 | 3.24 | 3.19 | 3.33 | 2.35 | -0.97 | 0.33 | 0.02 | small |
|  | Individual psychology | PL | 15,127 | 4,692 | 19,819 | 8.87 | 6.64 | 15.04 | 7.16 | -15.22 | 0.00 | 0.34 | moderate |
|  | Individual psychology | PT | 17,970 | 4,146 | 22,116 | 10.54 | 6.31 | 13.29 | 6.99 | -7.14 | 0.00 | 0.16 | small |
|  | Individual psychology | RU | 60,850 | 13,093 | 73,943 | 35.69 | 15.67 | 41.96 | 21.27 | -4.99 | 0.00 | 0.11 | small |
| **17** | Inner child | EN | 178,539 | 45,519 | 224,058 | 104.71 | 133.72 | 145.89 | 26.73 | -23.75 | 0.00 | 0.53 | large |
|  | Inner child | DE | 304,277 | 65,292 | 369,569 | 178.46 | 79.80 | 209.27 | 32.93 | -14.81 | 0.00 | 0.33 | moderate |
|  | Inner child | FR | 60,404 | 12,185 | 72,589 | 35.43 | 11.54 | 39.05 | 9.05 | -7.27 | 0.00 | 0.16 | small |
|  | Inner child | RU | 6,504 | 1,598 | 8,102 | 3.81 | 2.35 | 5.12 | 2.75 | -8.29 | 0.00 | 0.19 | small |
| **18** | Echopraxia | EN | 185,332 | 47,902 | 233,234 | 108.70 | 21.47 | 153.53 | 27.37 | -22.91 | 0.00 | 0.51 | large |
|  | Echopraxia | DE | 34,836 | 3,641 | 38,477 | 20.43 | 8.17 | 11.67 | 5.93 | 19.49 | 0.00 | 0.43 | moderate |
|  | Echopraxia | ES | 54,278 | 4,972 | 59,250 | 31.83 | 17.34 | 15.94 | 5.94 | 18.26 | 0.00 | 0.41 | moderate |
|  | Echopraxia | FR | 46,601 | 5,629 | 52,230 | 27.33 | 12.81 | 18.04 | 5.27 | 18.06 | 0.00 | 0.40 | moderate |
|  | Echopraxia | IT | 27,875 | 5,495 | 33,370 | 16.35 | 6.73 | 17.61 | 6.72 | -3.28 | 0.00 | 0.07 | small |
|  | Echopraxia | NL | 6,131 | 739 | 6,870 | 3.60 | 2.70 | 2.37 | 3.62 | 10.25 | 0.00 | 0.23 | small |
|  | Echopraxia | PL | 24,080 | 3,396 | 27,476 | 14.12 | 7.52 | 10.88 | 5.13 | 9.50 | 0.00 | 0.21 | small |
|  | Echopraxia | PT | 11,277 | 1,286 | 12,563 | 6.61 | 3.98 | 4.12 | 2.67 | 11.38 | 0.00 | 0.25 | small |
|  | Echopraxia | RU | 81,233 | 14,142 | 95,375 | 47.64 | 14.92 | 45.33 | 11.53 | 2.95 | 0.00 | 0.07 | small |
| **19** | Coprophilia | EN | 2,088,722 | 544,018 | 2,632,740 | 1225.06 | 500.15 | 1743.65 | 533.87 | -23.78 | 0.00 | 0.53 | large |
|  | Coprophilia | DE | 868,558 | 156,890 | 1,025,448 | 509.42 | 642.04 | 502.85 | 663.65 | 5.80 | 0.00 | 0.13 | small |
|  | Coprophilia | ES | 961,121 | 171,007 | 1,132,128 | 563.71 | 297.36 | 548.10 | 191.20 | 0.33 | 0.74 | 0.01 | small |
|  | Coprophilia | FR | 563,360 | 73,748 | 637,108 | 330.42 | 377.40 | 236.37 | 86.51 | 13.12 | 0.00 | 0.29 | small |
|  | Coprophilia | IT | 153,145 | 24,973 | 178,118 | 89.82 | 63.51 | 80.04 | 25.11 | 3.80 | 0.00 | 0.08 | small |
|  | Coprophilia | NL | 44,166 | 7,809 | 51,975 | 25.90 | 23.90 | 25.03 | 7.01 | -1.28 | 0.20 | 0.03 | small |
|  | Coprophilia | PL | 432,289 | 84,916 | 517,205 | 253.54 | 244.50 | 272.17 | 159.11 | -6.97 | 0.00 | 0.16 | small |
|  | Coprophilia | PT | 149,573 | 26,887 | 176,460 | 87.73 | 70.15 | 86.18 | 51.74 | 0.60 | 0.55 | 0.01 | small |
|  | Coprophilia | RU | 1,370,081 | 279,376 | 1,649,457 | 803.57 | 770.69 | 895.44 | 227.86 | -14.11 | 0.00 | 0.31 | moderate |
|  | Coprophilia | SV | 82,618 | 13,696 | 96,314 | 48.46 | 30.92 | 43.90 | 14.96 | 4.51 | 0.00 | 0.10 | small |
| **20** | Reciprocal liking | EN | 95,608 | 27,567 | 123,175 | 56.08 | 28.25 | 88.36 | 15.82 | -22.84 | 0.00 | 0.51 | large |
| **21** | Maladaptive daydreaming | EN | 1,083,984 | 436,336 | 1,520,320 | 635.77 | 643.37 | 1398.51 | 1242.41 | -21.81 | 0.00 | 0.49 | moderate |
|  | Maladaptive daydreaming | ES | 233,438 | 104,664 | 338,102 | 136.91 | 46.35 | 335.46 | 192.26 | -26.36 | 0.00 | 0.59 | large |
|  | Maladaptive daydreaming | FR | 27,428 | 23,486 | 50,914 | 16.09 | 27.19 | 75.28 | 48.78 | -26.44 | 0.00 | 0.60 | large |
|  | Maladaptive daydreaming | IT | 0 | 0 | 0 | 0.00 | 0.00 | 0.00 | 0.00 | 0.00 | 1.00 |  |  |
|  | Maladaptive daydreaming | PT | 42,063 | 30,192 | 72,255 | 24.67 | 22.19 | 96.77 | 52.74 | -26.63 | 0.00 | 0.59 | large |
|  | Maladaptive daydreaming | RU | 29,148 | 40,185 | 69,333 | 17.10 | 24.00 | 128.80 | 81.22 | -27.45 | 0.00 | 0.62 | large |
| **22** | Superman complex | EN | 57,532 | 16,958 | 74,490 | 33.74 | 12.02 | 54.35 | 12.98 | -21.96 | 0.00 | 0.49 | moderate |
| **23** | International Classification of Diseases | EN | 86,754 | 73,178 | 159,932 | 50.88 | 14.20 | 234.54 | 96.13 | -21.65 | 0.00 | 0.48 | moderate |
|  | International Classification of Diseases | DE | 1,559,491 | 228,041 | 1,787,532 | 914.66 | 261.91 | 730.90 | 158.94 | 12.29 | 0.00 | 0.27 | small |
|  | International Classification of Diseases | ES | 109,217 | 30,398 | 139,615 | 64.06 | 27.59 | 97.43 | 37.23 | -14.95 | 0.00 | 0.33 | moderate |
|  | International Classification of Diseases | FR | 327,930 | 59,889 | 387,819 | 192.33 | 70.35 | 191.95 | 86.86 | 3.65 | 0.00 | 0.08 | small |
|  | International Classification of Diseases | IT | 0 | 0 | 0 | 0.00 | 0.00 | 0.00 | 0.00 | 0.00 | 1.00 |  |  |
|  | International Classification of Diseases | NL | 14,722 | 2,330 | 17,052 | 8.63 | 13.86 | 7.47 | 11.22 | 5.20 | 0.00 | 0.12 | small |
|  | International Classification of Diseases | PL | 82,361 | 15,399 | 97,760 | 48.31 | 18.89 | 49.36 | 16.19 | -1.00 | 0.32 | 0.02 | small |
|  | International Classification of Diseases | PT | 283,456 | 56,067 | 339,523 | 166.25 | 198.87 | 179.70 | 76.04 | -6.84 | 0.00 | 0.15 | small |
|  | International Classification of Diseases | RU | 560,610 | 193,224 | 753,834 | 328.80 | 166.53 | 619.31 | 158.82 | -22.89 | 0.00 | 0.51 | large |
|  | International Classification of Diseases | SV | 26,074 | 4,898 | 30,972 | 15.29 | 7.04 | 15.70 | 6.74 | -1.41 | 0.16 | 0.03 | small |
| **24** | Logotherapy | EN | 865,548 | 215,888 | 1,081,436 | 507.65 | 109.85 | 691.95 | 108.07 | -21.84 | 0.00 | 0.49 | moderate |
|  | Logotherapy | DE | 231,153 | 45,585 | 276,738 | 135.57 | 54.75 | 146.11 | 81.58 | -4.48 | 0.00 | 0.10 | small |
|  | Logotherapy | ES | 638,018 | 115,247 | 753,265 | 374.20 | 105.37 | 369.38 | 90.04 | 0.37 | 0.71 | 0.01 | small |
|  | Logotherapy | FR | 62,561 | 13,283 | 75,844 | 36.69 | 13.78 | 42.57 | 10.00 | -9.19 | 0.00 | 0.20 | small |
|  | Logotherapy | IT | 33,582 | 5,511 | 39,093 | 19.70 | 7.48 | 17.66 | 6.69 | 4.62 | 0.00 | 0.10 | small |
|  | Logotherapy | PL | 54,072 | 13,486 | 67,558 | 31.71 | 13.99 | 43.22 | 12.96 | -14.42 | 0.00 | 0.32 | moderate |
|  | Logotherapy | PT | 160,145 | 39,681 | 199,826 | 93.93 | 33.72 | 127.18 | 29.05 | -15.70 | 0.00 | 0.35 | moderate |
|  | Logotherapy | RU | 156,404 | 31,815 | 188,219 | 91.73 | 23.64 | 101.97 | 28.27 | -5.11 | 0.00 | 0.11 | small |
| **25** | Shadow (psychology) | EN | 1,246,751 | 292,946 | 1,539,697 | 731.23 | 168.08 | 938.93 | 103.59 | -20.92 | 0.00 | 0.47 | moderate |
|  | Shadow (psychology) | DE | 102,898 | 26,360 | 129,258 | 60.35 | 22.10 | 84.49 | 16.35 | -18.20 | 0.00 | 0.41 | moderate |
|  | Shadow (psychology) | ES | 153,401 | 32,772 | 186,173 | 89.97 | 27.14 | 105.04 | 23.35 | -10.87 | 0.00 | 0.24 | small |
|  | Shadow (psychology) | FR | 82,837 | 16,351 | 99,188 | 48.58 | 27.57 | 52.41 | 14.20 | -5.46 | 0.00 | 0.12 | small |
|  | Shadow (psychology) | IT | 9,716 | 5,511 | 15,227 | 5.70 | 7.86 | 17.66 | 6.10 | -20.70 | 0.00 | 0.49 | moderate |
|  | Shadow (psychology) | NL | 16,528 | 4,457 | 20,985 | 9.69 | 4.97 | 14.29 | 4.92 | -14.64 | 0.00 | 0.33 | moderate |
|  | Shadow (psychology) | PL | 38,608 | 9,344 | 47,952 | 22.64 | 9.36 | 29.95 | 9.36 | -13.01 | 0.00 | 0.29 | small |
|  | Shadow (psychology) | PT | 50,097 | 12,975 | 63,072 | 29.38 | 10.69 | 41.59 | 11.34 | -16.74 | 0.00 | 0.37 | moderate |
|  | Shadow (psychology) | RU | 100,758 | 22,255 | 123,013 | 59.10 | 14.72 | 71.33 | 15.10 | -13.42 | 0.00 | 0.30 | small |
|  | Shadow (psychology) | SV | 6,942 | 1,512 | 8,454 | 4.07 | 3.26 | 4.85 | 2.97 | -4.94 | 0.00 | 0.11 | small |
| **26** | Dominance and submission | EN | 2,674,102 | 607,999 | 3,282,101 | 1568.39 | 292.17 | 1948.71 | 187.59 | -21.13 | 0.00 | 0.47 | moderate |
|  | Dominance and submission | ES | 608,145 | 135,208 | 743,353 | 356.68 | 80.86 | 433.36 | 113.65 | -13.07 | 0.00 | 0.29 | small |
|  | Dominance and submission | IT | 232,556 | 44,374 | 276,930 | 136.40 | 72.19 | 142.22 | 27.97 | -5.70 | 0.00 | 0.13 | small |
|  | Dominance and submission | PT | 260,794 | 76,790 | 337,584 | 152.96 | 78.43 | 246.12 | 107.96 | -14.23 | 0.00 | 0.32 | moderate |
| **27** | Man and His Symbols | EN | 128,409 | 32,448 | 160,857 | 75.31 | 19.41 | 104.00 | 15.35 | -21.30 | 0.00 | 0.47 | moderate |
|  | Man and His Symbols | ES | 39,653 | 5,899 | 45,552 | 23.26 | 15.70 | 18.91 | 7.67 | 6.80 | 0.00 | 0.15 | small |
|  | Man and His Symbols | PT | 14,647 | 2,517 | 17,164 | 8.59 | 4.20 | 8.07 | 3.97 | 2.07 | 0.04 | 0.05 | small |
| **28** | Body dysmorphic disorder | EN | 3,597,842 | 837,436 | 4,435,278 | 2110.17 | 1388.78 | 2684.09 | 454.37 | -21.69 | 0.00 | 0.48 | moderate |
|  | Body dysmorphic disorder | DE | 474,739 | 90,496 | 565,235 | 278.44 | 111.00 | 290.05 | 81.98 | -3.46 | 0.00 | 0.08 | small |
|  | Body dysmorphic disorder | ES | 720,021 | 85,015 | 805,036 | 422.30 | 318.69 | 272.48 | 54.56 | 17.17 | 0.00 | 0.38 | moderate |
|  | Body dysmorphic disorder | IT | 295,601 | 84,553 | 380,154 | 173.37 | 147.38 | 271.00 | 83.46 | -23.14 | 0.00 | 0.52 | large |
|  | Body dysmorphic disorder | NL | 100,087 | 18,829 | 118,916 | 58.70 | 25.37 | 60.35 | 25.91 | -2.25 | 0.02 | 0.05 | small |
|  | Body dysmorphic disorder | PL | 12,293 | 49,729 | 62,022 | 7.21 | 27.59 | 159.39 | 44.84 | -27.66 | 0.00 | 0.85 | large |
|  | Body dysmorphic disorder | PT | 130,579 | 23,548 | 154,127 | 76.59 | 109.56 | 75.47 | 22.99 | -2.43 | 0.02 | 0.05 | small |
|  | Body dysmorphic disorder | RU | 744,244 | 208,138 | 952,382 | 436.51 | 275.81 | 667.11 | 202.93 | -22.10 | 0.00 | 0.49 | moderate |
|  | Body dysmorphic disorder | SV | 196,559 | 17,728 | 214,287 | 115.28 | 65.26 | 56.82 | 41.11 | 24.95 | 0.00 | 0.56 | large |
| **29** | Psychic driving | ES | 128,519 | 37,674 | 166,193 | 75.38 | 28.06 | 120.75 | 48.43 | -21.39 | 0.00 | 0.48 | moderate |
|  | Psychic driving | PL | 3,881 | 726 | 4,607 | 2.28 | 2.00 | 2.33 | 1.88 | -0.71 | 0.48 | 0.02 | small |
| **30** | Fooled by Randomness | EN | 246,335 | 64,568 | 310,903 | 144.48 | 100.67 | 206.95 | 49.48 | -22.39 | 0.00 | 0.50 | moderate |
|  | Fooled by Randomness | FR | 3,288 | 3,250 | 6,538 | 1.93 | 3.17 | 10.42 | 6.13 | -24.49 | 0.00 | 0.59 | large |
|  | Fooled by Randomness | RU | 36,830 | 10,155 | 46,985 | 21.60 | 104.89 | 32.55 | 12.72 | -17.27 | 0.00 | 0.39 | moderate |
| **31** | Subliminal perception | SV | 7,721 | 2,933 | 10,654 | 4.53 | 3.58 | 9.40 | 3.54 | -19.76 | 0.00 | 0.44 | moderate |
| **32** | Stimming | DE | 969,180 | 290,548 | 1,259,728 | 568.43 | 830.05 | 931.24 | 316.74 | -22.14 | 0.00 | 0.49 | moderate |
|  | Stimming | ES | 17,149 | 10,526 | 27,675 | 10.06 | 9.65 | 33.74 | 11.47 | -25.99 | 0.00 | 0.58 | large |
|  | Stimming | NL | 33,325 | 7,707 | 41,032 | 19.55 | 8.17 | 24.70 | 7.22 | -12.06 | 0.00 | 0.27 | small |
|  | Stimming | RU | 37,040 | 17,431 | 54,471 | 21.72 | 14.43 | 55.87 | 13.31 | -25.86 | 0.00 | 0.58 | large |
| **33** | Rashomon effect | EN | 699,584 | 180,924 | 880,508 | 410.31 | 200.35 | 579.88 | 177.81 | -20.73 | 0.00 | 0.46 | moderate |
|  | Rashomon effect | ES | 39,803 | 13,688 | 53,491 | 23.34 | 9.22 | 43.87 | 12.27 | -23.66 | 0.00 | 0.53 | large |
|  | Rashomon effect | IT | 3,049 | 3,111 | 6,160 | 1.79 | 4.68 | 9.97 | 9.18 | -23.27 | 0.00 | 0.62 | large |
|  | Rashomon effect | NL | 3,969 | 685 | 4,654 | 2.33 | 2.13 | 2.20 | 1.87 | 0.74 | 0.46 | 0.02 | small |
|  | Rashomon effect | PL | 2,659 | 960 | 3,619 | 1.56 | 2.57 | 3.08 | 2.73 | -12.67 | 0.00 | 0.31 | moderate |
|  | Rashomon effect | RU | 10,246 | 1,922 | 12,168 | 6.01 | 4.74 | 6.16 | 3.58 | -1.82 | 0.07 | 0.04 | small |
| **34** | Cinderella complex | EN | 212,504 | 50,400 | 262,904 | 124.64 | 78.33 | 161.54 | 29.47 | -20.87 | 0.00 | 0.46 | moderate |
|  | Cinderella complex | DE | 59,380 | 10,367 | 69,747 | 34.83 | 18.20 | 33.23 | 13.26 | 2.28 | 0.02 | 0.05 | small |
|  | Cinderella complex | ES | 59,639 | 8,471 | 68,110 | 34.98 | 18.15 | 27.15 | 9.37 | 6.63 | 0.00 | 0.15 | small |
|  | Cinderella complex | FR | 30,271 | 5,647 | 35,918 | 17.75 | 12.40 | 18.10 | 13.91 | 0.58 | 0.56 | 0.01 | small |
| **35** | Acrophobia | EN | 1,773,301 | 419,964 | 2,193,265 | 1040.06 | 267.11 | 1346.04 | 244.58 | -20.53 | 0.00 | 0.46 | moderate |
|  | Acrophobia | DE | 201,009 | 35,912 | 236,921 | 117.89 | 36.76 | 115.10 | 99.50 | 4.92 | 0.00 | 0.11 | small |
|  | Acrophobia | ES | 306,697 | 41,302 | 347,999 | 179.88 | 65.71 | 132.38 | 46.45 | 14.61 | 0.00 | 0.33 | moderate |
|  | Acrophobia | FR | 160,408 | 30,317 | 190,725 | 94.08 | 64.45 | 97.17 | 30.75 | -4.02 | 0.00 | 0.09 | small |
|  | Acrophobia | IT | 72,430 | 13,485 | 85,915 | 42.48 | 18.04 | 43.22 | 22.12 | -0.78 | 0.43 | 0.02 | small |
|  | Acrophobia | NL | 46,311 | 8,220 | 54,531 | 27.16 | 64.53 | 26.35 | 8.53 | -2.78 | 0.01 | 0.06 | small |
|  | Acrophobia | PT | 76,571 | 12,141 | 88,712 | 44.91 | 27.85 | 38.91 | 13.69 | 3.50 | 0.00 | 0.08 | small |
|  | Acrophobia | RU | 172,913 | 37,699 | 210,612 | 101.42 | 40.69 | 120.83 | 34.26 | -11.04 | 0.00 | 0.25 | small |
| **36** | Reactance (psychology) | EN | 315,261 | 92,738 | 407,999 | 184.90 | 117.78 | 297.24 | 449.34 | -20.28 | 0.00 | 0.45 | moderate |
|  | Reactance (psychology) | DE | 215,018 | 62,780 | 277,798 | 126.11 | 35.21 | 201.22 | 196.68 | -14.73 | 0.00 | 0.33 | moderate |
|  | Reactance (psychology) | ES | 177,457 | 42,362 | 219,819 | 104.08 | 116.31 | 135.78 | 201.24 | -4.66 | 0.00 | 0.10 | small |
|  | Reactance (psychology) | FR | 57,092 | 22,080 | 79,172 | 33.49 | 27.31 | 70.77 | 32.93 | -22.01 | 0.00 | 0.49 | moderate |
|  | Reactance (psychology) | PL | 43,535 | 10,159 | 53,694 | 25.53 | 55.09 | 32.56 | 18.24 | -9.39 | 0.00 | 0.21 | small |
|  | Reactance (psychology) | PT | 8,232 | 2,264 | 10,496 | 4.83 | 4.62 | 7.26 | 5.14 | -9.41 | 0.00 | 0.21 | small |
|  | Reactance (psychology) | RU | 20,780 | 5,894 | 26,674 | 12.19 | 8.32 | 18.89 | 7.83 | -12.60 | 0.00 | 0.28 | small |
| **37** | Narcissistic parent | EN | 988,501 | 276,049 | 1,264,550 | 579.77 | 369.55 | 884.77 | 185.82 | -20.29 | 0.00 | 0.45 | moderate |
|  | Narcissistic parent | DE | 61,498 | 45,245 | 106,743 | 36.07 | 52.27 | 145.02 | 68.93 | -23.95 | 0.00 | 0.56 | large |
|  | Narcissistic parent | ES | 15,038 | 17,023 | 32,061 | 8.82 | 16.57 | 54.56 | 29.61 | -24.50 | 0.00 | 0.64 | large |
|  | Narcissistic parent | IT | 41,257 | 12,058 | 53,315 | 24.20 | 23.08 | 38.65 | 11.28 | -13.43 | 0.00 | 0.30 | small |
|  | Narcissistic parent | PL | 23,197 | 4,090 | 27,287 | 13.61 | 7.97 | 13.11 | 5.41 | 0.37 | 0.71 | 0.01 | small |
| **38** | Righteous indignation | EN | 261,560 | 73,959 | 335,519 | 153.41 | 50.13 | 237.05 | 244.76 | -20.17 | 0.00 | 0.45 | moderate |
| **39** | Silva Method | EN | 237,987 | 89,505 | 327,492 | 139.58 | 44.34 | 286.88 | 114.34 | -20.79 | 0.00 | 0.46 | moderate |
|  | Silva Method | ES | 175,710 | 55,419 | 231,129 | 103.06 | 24.22 | 177.62 | 99.45 | -14.60 | 0.00 | 0.33 | moderate |
|  | Silva Method | FR | 13,324 | 5,485 | 18,809 | 7.81 | 4.87 | 17.58 | 12.90 | -14.77 | 0.00 | 0.33 | moderate |
|  | Silva Method | NL | 2,330 | 629 | 2,959 | 1.37 | 1.52 | 2.02 | 1.79 | -6.16 | 0.00 | 0.14 | small |
|  | Silva Method | PL | 65,169 | 10,013 | 75,182 | 38.22 | 11.39 | 32.09 | 10.27 | 9.39 | 0.00 | 0.21 | small |
| **40** | Chronophilia | EN | 391,291 | 96,969 | 488,260 | 229.50 | 58.81 | 310.80 | 80.06 | -19.98 | 0.00 | 0.44 | moderate |
|  | Chronophilia | DE | 55,220 | 13,183 | 68,403 | 32.39 | 11.56 | 42.25 | 15.75 | -13.98 | 0.00 | 0.31 | moderate |
|  | Chronophilia | ES | 296,509 | 63,978 | 360,487 | 173.91 | 46.17 | 205.06 | 62.70 | -7.38 | 0.00 | 0.16 | small |
|  | Chronophilia | FR | 52,622 | 10,363 | 62,985 | 30.86 | 13.83 | 33.21 | 7.91 | -6.88 | 0.00 | 0.15 | small |
|  | Chronophilia | PL | 38,963 | 13,747 | 52,710 | 22.85 | 32.71 | 44.06 | 13.16 | -23.59 | 0.00 | 0.53 | large |
|  | Chronophilia | PT | 38,886 | 5,274 | 44,160 | 22.81 | 13.14 | 16.90 | 8.65 | 6.56 | 0.00 | 0.15 | small |
|  | Chronophilia | SV | 4,679 | 917 | 5,596 | 2.74 | 2.16 | 2.94 | 2.03 | -2.19 | 0.03 | 0.05 | small |
| **41** | Cassandra (metaphor) | EN | 528,706 | 128,354 | 657,060 | 310.09 | 516.67 | 411.39 | 174.55 | -20.45 | 0.00 | 0.46 | moderate |
|  | Cassandra (metaphor) | FR | 99,938 | 29,270 | 129,208 | 58.61 | 44.04 | 93.81 | 53.32 | -13.60 | 0.00 | 0.30 | moderate |
|  | Cassandra (metaphor) | IT | 63,231 | 14,424 | 77,655 | 37.09 | 23.23 | 46.23 | 29.27 | -8.54 | 0.00 | 0.19 | small |
|  | Cassandra (metaphor) | PL | 43,491 | 10,052 | 53,543 | 25.51 | 11.56 | 32.22 | 13.23 | -9.32 | 0.00 | 0.21 | small |
|  | Cassandra (metaphor) | PT | 41,109 | 15,728 | 56,837 | 24.11 | 16.40 | 50.41 | 68.51 | -19.24 | 0.00 | 0.43 | moderate |
|  | Cassandra (metaphor) | RU | 172,380 | 12,172 | 184,552 | 101.10 | 116.68 | 39.01 | 15.73 | 20.84 | 0.00 | 0.46 | moderate |
|  | Cassandra (metaphor) | SV | 4,213 | 1,388 | 5,601 | 2.47 | 3.24 | 4.45 | 4.61 | -10.07 | 0.00 | 0.23 | small |
| **42** | Collective identity | EN | 148,443 | 48,967 | 197,410 | 87.06 | 34.46 | 156.95 | 63.84 | -20.31 | 0.00 | 0.45 | moderate |
|  | Collective identity | DE | 19,131 | 7,427 | 26,558 | 11.22 | 13.54 | 23.80 | 9.21 | -15.77 | 0.00 | 0.36 | moderate |
|  | Collective identity | RU | 173,090 | 58,110 | 231,200 | 101.52 | 76.20 | 186.25 | 150.16 | -9.65 | 0.00 | 0.21 | small |
| **43** | Transvestic fetishism | EN | 314,613 | 69,667 | 384,280 | 184.52 | 31.50 | 223.29 | 30.89 | -18.61 | 0.00 | 0.41 | moderate |
|  | Transvestic fetishism | DE | 67,306 | 11,464 | 78,770 | 39.48 | 11.79 | 36.74 | 8.07 | 4.59 | 0.00 | 0.10 | small |
|  | Transvestic fetishism | ES | 111,242 | 34,929 | 146,171 | 65.24 | 19.41 | 111.95 | 20.83 | -25.03 | 0.00 | 0.56 | large |
|  | Transvestic fetishism | FR | 23,471 | 4,788 | 28,259 | 13.77 | 7.54 | 15.35 | 5.45 | -5.30 | 0.00 | 0.12 | small |
|  | Transvestic fetishism | IT | 29,370 | 4,409 | 33,779 | 17.23 | 8.04 | 14.13 | 5.86 | 7.14 | 0.00 | 0.16 | small |
|  | Transvestic fetishism | PL | 18,501 | 7,079 | 25,580 | 10.85 | 21.28 | 22.69 | 9.03 | -20.75 | 0.00 | 0.46 | moderate |
|  | Transvestic fetishism | PT | 9,221 | 2,433 | 11,654 | 5.41 | 4.96 | 7.80 | 3.64 | -9.32 | 0.00 | 0.21 | small |
|  | Transvestic fetishism | RU | 202,085 | 40,561 | 242,646 | 118.52 | 24.25 | 130.00 | 18.73 | -9.86 | 0.00 | 0.22 | small |
| **44** | Thought insertion | EN | 133,814 | 34,113 | 167,927 | 78.48 | 22.61 | 109.34 | 25.87 | -19.18 | 0.00 | 0.43 | moderate |
|  | Thought insertion | DE | 46,612 | 5,169 | 51,781 | 27.34 | 10.19 | 16.57 | 5.62 | 18.82 | 0.00 | 0.42 | moderate |
| **45** | Infradian rhythm | EN | 36,518 | 11,877 | 48,395 | 21.42 | 11.98 | 38.07 | 17.78 | -18.93 | 0.00 | 0.42 | moderate |
|  | Infradian rhythm | DE | 11,268 | 2,090 | 13,358 | 6.61 | 3.69 | 6.70 | 4.43 | 0.25 | 0.80 | 0.01 | small |
|  | Infradian rhythm | ES | 34,132 | 5,466 | 39,598 | 20.02 | 12.55 | 17.52 | 10.21 | 3.30 | 0.00 | 0.07 | small |
|  | Infradian rhythm | FR | 16,671 | 5,446 | 22,117 | 9.78 | 7.19 | 17.46 | 10.80 | -12.35 | 0.00 | 0.28 | small |
|  | Infradian rhythm | IT | 6,041 | 1,062 | 7,103 | 3.54 | 3.01 | 3.40 | 2.44 | -0.33 | 0.74 | 0.01 | small |
| **46** | Homophobia | EN | 2,012,289 | 440,669 | 2,452,958 | 1180.23 | 937.55 | 1412.40 | 241.21 | -19.36 | 0.00 | 0.43 | moderate |
|  | Homophobia | DE | 1,084,762 | 231,187 | 1,315,949 | 636.22 | 939.24 | 740.98 | 525.92 | -13.20 | 0.00 | 0.29 | small |
|  | Homophobia | ES | 2,199,270 | 376,556 | 2,575,826 | 1289.89 | 731.51 | 1206.91 | 572.34 | 2.75 | 0.01 | 0.06 | small |
|  | Homophobia | FR | 558,488 | 88,219 | 646,707 | 327.56 | 223.49 | 282.75 | 98.19 | 3.93 | 0.00 | 0.09 | small |
|  | Homophobia | IT | 558,951 | 107,622 | 666,573 | 327.83 | 286.36 | 344.94 | 349.57 | -4.66 | 0.00 | 0.10 | small |
|  | Homophobia | NL | 76,741 | 16,346 | 93,087 | 45.01 | 43.42 | 52.39 | 42.86 | -7.30 | 0.00 | 0.16 | small |
|  | Homophobia | PL | 366,791 | 116,943 | 483,734 | 215.13 | 144.64 | 374.82 | 332.27 | -14.15 | 0.00 | 0.32 | moderate |
|  | Homophobia | PT | 823,330 | 115,846 | 939,176 | 482.89 | 245.81 | 371.30 | 268.66 | 10.64 | 0.00 | 0.24 | small |
|  | Homophobia | RU | 1,182,614 | 236,196 | 1,418,810 | 693.62 | 233.40 | 757.04 | 237.89 | -6.31 | 0.00 | 0.14 | small |
|  | Homophobia | SV | 52,120 | 6,722 | 58,842 | 30.57 | 21.84 | 21.54 | 9.75 | 10.62 | 0.00 | 0.24 | small |
| **47** | Philomath | EN | 221,852 | 55,996 | 277,848 | 130.12 | 41.58 | 179.47 | 87.90 | -19.64 | 0.00 | 0.44 | moderate |
|  | Philomath | ES | 740 | 784 | 1,524 | 0.43 | 1.14 | 2.51 | 2.02 | -19.23 | 0.00 | 0.52 | large |
|  | Philomath | FR | 218 | 198 | 416 | 0.13 | 0.51 | 0.63 | 0.87 | -10.03 | 0.00 | 0.37 | moderate |
| **48** | Complex (psychology) | EN | 521,242 | 125,993 | 647,235 | 305.71 | 50.84 | 403.82 | 88.04 | -18.59 | 0.00 | 0.41 | moderate |
|  | Complex (psychology) | DE | 210,210 | 25,855 | 236,065 | 123.29 | 25.91 | 82.87 | 15.77 | 23.60 | 0.00 | 0.53 | large |
|  | Complex (psychology) | ES | 309,440 | 40,307 | 349,747 | 181.49 | 46.82 | 129.19 | 32.33 | 19.04 | 0.00 | 0.42 | moderate |
|  | Complex (psychology) | FR | 51,763 | 11,430 | 63,193 | 30.36 | 9.94 | 36.63 | 9.99 | -10.08 | 0.00 | 0.22 | small |
|  | Complex (psychology) | NL | 64,729 | 11,224 | 75,953 | 37.96 | 10.95 | 35.97 | 9.68 | 3.19 | 0.00 | 0.07 | small |
|  | Complex (psychology) | PL | 140,291 | 23,535 | 163,826 | 82.28 | 20.05 | 75.43 | 24.19 | 5.87 | 0.00 | 0.13 | small |
|  | Complex (psychology) | PT | 37,004 | 9,151 | 46,155 | 21.70 | 10.50 | 29.33 | 9.71 | -14.03 | 0.00 | 0.31 | moderate |
|  | Complex (psychology) | RU | 208,095 | 42,947 | 251,042 | 122.05 | 23.17 | 137.65 | 22.90 | -10.50 | 0.00 | 0.23 | small |
|  | Complex (psychology) | SV | 22,003 | 4,184 | 26,187 | 12.90 | 6.22 | 13.41 | 5.58 | -1.48 | 0.14 | 0.03 | small |
| **49** | Thought-terminating cliché | EN | 178,163 | 62,005 | 240,168 | 104.49 | 194.10 | 198.73 | 288.50 | -19.48 | 0.00 | 0.43 | moderate |
|  | Thought-terminating cliché | DE | 151,943 | 31,885 | 183,828 | 89.12 | 35.09 | 102.20 | 25.04 | -9.29 | 0.00 | 0.21 | small |
|  | Thought-terminating cliché | NL | 18,484 | 2,774 | 21,258 | 10.84 | 5.36 | 8.89 | 4.02 | 6.27 | 0.00 | 0.14 | small |
|  | Thought-terminating cliché | SV | 2,717 | 570 | 3,287 | 1.59 | 2.02 | 1.83 | 1.97 | -2.08 | 0.04 | 0.05 | small |
| **50** | Apoplexy | EN | 1,180,264 | 285,909 | 1,466,173 | 692.24 | 195.24 | 916.38 | 277.12 | -18.92 | 0.00 | 0.42 | moderate |
|  | Apoplexy | ES | 275,919 | 21,055 | 296,974 | 161.83 | 68.93 | 67.48 | 15.78 | 24.08 | 0.00 | 0.54 | large |
|  | Apoplexy | RU | 537,787 | 95,173 | 632,960 | 315.42 | 156.26 | 305.04 | 105.28 | 1.63 | 0.10 | 0.04 | small |

**Table 2**

*Top 50 Articles in English and their Equivalents with the Largest Decrease in Viewership*

|  | **Page name** | **Language** | **Absolute sum before pandemic** | **Absolute sum pandemic** | **Absolute total sums** | **Daily mean before pandemic** | **Daily std before pandemic** | **Daily mean during pandemic** | **Daily std during pandemic** | **W_stat** | **p_value** | **effect_size** | **effect_magnitude** |
| --- | --- | --- | --- | --- | --- | --- | --- | --- | --- | --- | --- | --- | --- |
| **1** | Sensation (psychology) | EN | 385,578 | 15,498 | 401,076 | 226.15 | 72.73 | 49.67 | 38.71 | 27.17 | 0.00 | 0.60 | large |
| **2** | Levels-of-processing effect | EN | 192,888 | 5,577 | 198,465 | 113.13 | 50.45 | 17.88 | 7.88 | 28.03 | 0.00 | 0.62 | large |
|  | Levels-of-processing effect | RU | 17,745 | 3,094 | 20,839 | 10.41 | 5.92 | 9.92 | 5.44 | 0.63 | 0.53 | 0.01 | small |
| **3** | Coping (psychology) | EN | 1,012,413 | 35,685 | 1,048,098 | 593.79 | 224.64 | 114.38 | 24.90 | 27.34 | 0.00 | 0.61 | large |
| **4** | Impulse control disorder | EN | 900,689 | 54,951 | 955,640 | 528.26 | 125.70 | 176.12 | 112.39 | 27.40 | 0.00 | 0.61 | large |
|  | Impulse control disorder | FR | 387,356 | 72,473 | 459,829 | 227.19 | 61.27 | 232.29 | 45.98 | -2.51 | 0.01 | 0.06 | small |
|  | Impulse control disorder | IT | 34,150 | 8,125 | 42,275 | 20.03 | 8.49 | 26.04 | 7.81 | -11.48 | 0.00 | 0.26 | small |
|  | Impulse control disorder | PT | 49,092 | 9,103 | 58,195 | 28.79 | 10.39 | 29.18 | 8.45 | -1.35 | 0.18 | 0.03 | small |
|  | Impulse control disorder | SV | 24,207 | 3,241 | 27,448 | 14.20 | 5.75 | 10.39 | 3.95 | 11.75 | 0.00 | 0.26 | small |
| **5** | ESFJ | EN | 604,327 | 7,859 | 612,186 | 354.44 | 107.06 | 25.19 | 25.00 | 28.04 | 0.00 | 0.62 | large |
| **6** | Sibling | EN | 1,403,194 | 158,752 | 1,561,946 | 822.99 | 204.26 | 508.82 | 108.88 | 26.56 | 0.00 | 0.59 | large |
|  | Sibling | ES | 507,169 | 83,746 | 590,915 | 297.46 | 82.18 | 268.42 | 38.70 | 6.73 | 0.00 | 0.15 | small |
|  | Sibling | FR | 742,461 | 128,849 | 871,310 | 435.46 | 84.22 | 412.98 | 116.40 | 5.92 | 0.00 | 0.13 | small |
|  | Sibling | NL | 14,982 | 3,199 | 18,181 | 8.79 | 5.89 | 10.25 | 4.97 | -5.85 | 0.00 | 0.13 | small |
|  | Sibling | PL | 95,534 | 21,902 | 117,436 | 56.03 | 16.17 | 70.20 | 17.21 | -15.08 | 0.00 | 0.34 | moderate |
|  | Sibling | PT | 65,088 | 16,803 | 81,891 | 38.17 | 38.85 | 53.86 | 182.61 | -5.40 | 0.00 | 0.12 | small |
|  | Sibling | RU | 650,176 | 138,883 | 789,059 | 381.33 | 78.14 | 445.14 | 87.61 | -14.67 | 0.00 | 0.33 | moderate |
|  | Sibling | SV | 18,812 | 3,748 | 22,560 | 11.03 | 5.20 | 12.01 | 5.02 | -3.75 | 0.00 | 0.08 | small |
| **7** | INFP | SV | 2,419,061 | 33,144 | 2,452,205 | 1418.80 | 410.04 | 106.23 | 121.83 | 28.05 | 0.00 | 0.62 | large |
| **8** | Burnout (psychology) | EN | 443,345 | 24,316 | 467,661 | 260.03 | 199.01 | 77.94 | 19.68 | 27.11 | 0.00 | 0.60 | large |
|  | Burnout (psychology) | DE | 805,114 | 115,694 | 920,808 | 472.21 | 392.78 | 370.81 | 85.85 | 6.34 | 0.00 | 0.14 | small |
|  | Burnout (psychology) | ES | 387,070 | 54,928 | 441,998 | 227.02 | 290.67 | 176.05 | 46.37 | -2.17 | 0.03 | 0.05 | small |
|  | Burnout (psychology) | FR | 614,134 | 35,552 | 649,686 | 360.20 | 180.98 | 113.95 | 27.38 | 26.32 | 0.00 | 0.59 | large |
|  | Burnout (psychology) | IT | 1,180,144 | 157,537 | 1,337,681 | 692.17 | 644.38 | 504.93 | 510.14 | 13.08 | 0.00 | 0.29 | small |
|  | Burnout (psychology) | NL | 265,348 | 26,238 | 291,586 | 155.63 | 46.30 | 84.10 | 23.68 | 24.36 | 0.00 | 0.54 | large |
|  | Burnout (psychology) | PL | 122,765 | 13,370 | 136,135 | 72.00 | 33.81 | 42.85 | 15.99 | 16.78 | 0.00 | 0.37 | moderate |
|  | Burnout (psychology) | PT | 1,224,698 | 178,854 | 1,403,552 | 718.30 | 549.81 | 573.25 | 240.84 | 5.35 | 0.00 | 0.12 | small |
|  | Burnout (psychology) | RU | 576,046 | 175,752 | 751,798 | 337.86 | 233.01 | 563.31 | 246.98 | -19.07 | 0.00 | 0.42 | moderate |
|  | Burnout (psychology) | SV | 74,902 | 7,053 | 81,955 | 43.93 | 14.56 | 22.61 | 8.05 | 22.97 | 0.00 | 0.51 | large |
| **9** | Engram | EN | 102,193 | 6,027 | 108,220 | 59.94 | 36.12 | 19.32 | 7.41 | 27.11 | 0.00 | 0.60 | large |
|  | Engram | ES | 2,826 | 417 | 3,243 | 1.66 | 1.56 | 1.34 | 1.37 | 3.37 | 0.00 | 0.08 | small |
| **10** | Hyperprosexia | EN | 15,241 | 1 | 15,242 | 8.94 | 7.22 | 0.00 | 0.06 | 26.95 | 0.00 | 0.60 | large |
| **11** | Stir crazy (condition) | EN | 391,240 | 5,971 | 397,211 | 229.47 | 131.22 | 19.14 | 35.51 | 26.72 | 0.00 | 0.60 | large |
|  | Stir crazy (condition) | ES | 0 | 4,252 | 4,252 | 0.00 | 0.00 | 13.63 | 22.63 | -8.65 | 0.00 | 0.52 | large |
|  | Stir crazy (condition) | FR | 0 | 36,354 | 36,354 | 0.00 | 0.00 | 116.52 | 372.76 | -21.63 | 0.00 | 0.86 | large |
|  | Stir crazy (condition) | RU | 9,372 | 7,900 | 17,272 | 5.50 | 7.84 | 25.32 | 23.81 | -22.48 | 0.00 | 0.53 | large |
|  | Stir crazy (condition) | SV | 39,177 | 14,673 | 53,850 | 22.98 | 12.14 | 47.03 | 52.30 | -13.18 | 0.00 | 0.29 | small |
| **12** | Personality tests | EN | 63,591 | 3,207 | 66,798 | 37.30 | 13.30 | 10.28 | 4.20 | 26.54 | 0.00 | 0.59 | large |
| **13** | Value (personal and cultural) | EN | 402,586 | 15,703 | 418,289 | 236.12 | 292.71 | 50.33 | 31.15 | 26.56 | 0.00 | 0.59 | large |
| **14** | Mental function | EN | 50,136 | 2,225 | 52,361 | 29.41 | 12.69 | 7.13 | 6.74 | 26.63 | 0.00 | 0.59 | large |
|  | Mental function | DE | 1,729,136 | 157,065 | 1,886,201 | 1014.16 | 352.12 | 503.41 | 96.02 | 24.86 | 0.00 | 0.55 | large |
|  | Mental function | ES | 1,516,995 | 184,358 | 1,701,353 | 889.73 | 394.97 | 590.89 | 173.60 | 13.83 | 0.00 | 0.31 | moderate |
|  | Mental function | FR | 549,164 | 86,050 | 635,214 | 322.09 | 87.14 | 275.80 | 65.67 | 9.02 | 0.00 | 0.20 | small |
|  | Mental function | IT | 136,206 | 21,071 | 157,277 | 79.89 | 23.72 | 67.54 | 19.05 | 8.59 | 0.00 | 0.19 | small |
|  | Mental function | NL | 201,467 | 27,255 | 228,722 | 118.16 | 42.23 | 87.36 | 24.61 | 12.70 | 0.00 | 0.28 | small |
|  | Mental function | PL | 410,236 | 79,135 | 489,371 | 240.61 | 102.90 | 253.64 | 110.20 | -2.20 | 0.03 | 0.05 | small |
|  | Mental function | PT | 550,784 | 66,961 | 617,745 | 323.04 | 136.54 | 214.62 | 54.57 | 13.74 | 0.00 | 0.31 | moderate |
|  | Mental function | RU | 632,307 | 109,253 | 741,560 | 370.85 | 216.87 | 350.17 | 162.36 | 0.27 | 0.79 | 0.01 | small |
|  | Mental function | SV | 287,556 | 29,413 | 316,969 | 168.65 | 86.54 | 94.27 | 35.51 | 16.27 | 0.00 | 0.36 | moderate |
| **15** | Separation anxiety disorder | EN | 1,022,394 | 93,461 | 1,115,855 | 599.64 | 172.24 | 299.55 | 49.84 | 26.91 | 0.00 | 0.60 | large |
|  | Separation anxiety disorder | DE | 33,434 | 5,448 | 38,882 | 19.61 | 6.63 | 17.46 | 5.39 | 5.14 | 0.00 | 0.11 | small |
|  | Separation anxiety disorder | ES | 131,578 | 12,479 | 144,057 | 77.17 | 38.04 | 40.00 | 19.74 | 22.70 | 0.00 | 0.51 | large |
|  | Separation anxiety disorder | FR | 45,477 | 6,128 | 51,605 | 26.67 | 7.68 | 19.64 | 5.99 | 15.58 | 0.00 | 0.35 | moderate |
|  | Separation anxiety disorder | IT | 47,226 | 8,004 | 55,230 | 27.70 | 10.77 | 25.65 | 7.06 | 2.88 | 0.00 | 0.06 | small |
|  | Separation anxiety disorder | NL | 38,355 | 3,894 | 42,249 | 22.50 | 7.06 | 12.48 | 5.04 | 21.38 | 0.00 | 0.48 | moderate |
|  | Separation anxiety disorder | PL | 8,710 | 3,123 | 11,833 | 5.11 | 3.20 | 10.01 | 5.10 | -17.06 | 0.00 | 0.38 | moderate |
|  | Separation anxiety disorder | PT | 11,233 | 6,531 | 17,764 | 6.59 | 11.08 | 20.93 | 6.72 | -20.16 | 0.00 | 0.49 | moderate |
|  | Separation anxiety disorder | RU | 7,175 | 7,915 | 15,090 | 4.21 | 8.96 | 25.37 | 7.86 | -24.27 | 0.00 | 0.65 | large |
| **16** | Pathological lying | EN | 2,866,863 | 214,714 | 3,081,577 | 1681.44 | 3757.68 | 688.19 | 93.68 | 26.99 | 0.00 | 0.60 | large |
|  | Pathological lying | DE | 357,262 | 71,371 | 428,633 | 209.54 | 139.29 | 228.75 | 50.00 | -9.78 | 0.00 | 0.22 | small |
|  | Pathological lying | ES | 1,314,379 | 146,220 | 1,460,599 | 770.90 | 202.86 | 468.65 | 125.37 | 25.08 | 0.00 | 0.56 | large |
|  | Pathological lying | FR | 530,936 | 52,898 | 583,834 | 311.40 | 98.21 | 169.54 | 42.70 | 25.70 | 0.00 | 0.57 | large |
|  | Pathological lying | IT | 139,767 | 31,149 | 170,916 | 81.97 | 41.93 | 99.84 | 20.82 | -14.61 | 0.00 | 0.33 | moderate |
|  | Pathological lying | NL | 106,040 | 18,672 | 124,712 | 62.19 | 26.36 | 59.85 | 14.77 | 1.44 | 0.15 | 0.03 | small |
|  | Pathological lying | PL | 269,464 | 30,600 | 300,064 | 158.04 | 188.47 | 98.08 | 25.94 | 18.86 | 0.00 | 0.42 | moderate |
|  | Pathological lying | PT | 473,760 | 55,642 | 529,402 | 277.87 | 230.03 | 178.34 | 57.12 | 21.70 | 0.00 | 0.48 | moderate |
|  | Pathological lying | RU | 229,649 | 64,426 | 294,075 | 134.69 | 67.65 | 206.49 | 32.28 | -20.48 | 0.00 | 0.46 | moderate |
|  | Pathological lying | SV | 166,858 | 41,324 | 208,182 | 97.86 | 40.40 | 132.45 | 38.29 | -16.90 | 0.00 | 0.38 | moderate |
| **17** | Denial | EN | 539,118 | 53,494 | 592,612 | 316.20 | 102.18 | 171.46 | 34.08 | 26.58 | 0.00 | 0.59 | large |
| **18** | Narcissistic rage | EN | 135,431 | 8,401 | 143,832 | 79.43 | 19.75 | 26.93 | 17.86 | 26.20 | 0.00 | 0.58 | large |
|  | Narcissistic rage | DE | 2,147,330 | 388,042 | 2,535,372 | 1259.43 | 813.23 | 1243.72 | 211.67 | -0.20 | 0.84 | 0.00 | small |
|  | Narcissistic rage | ES | 472,020 | 52,815 | 524,835 | 276.84 | 101.93 | 169.28 | 25.43 | 20.69 | 0.00 | 0.46 | moderate |
|  | Narcissistic rage | FR | 943,396 | 257,081 | 1,200,477 | 553.31 | 184.78 | 823.98 | 155.12 | -21.56 | 0.00 | 0.48 | moderate |
|  | Narcissistic rage | IT | 560,154 | 85,879 | 646,033 | 328.54 | 63.05 | 275.25 | 57.93 | 15.63 | 0.00 | 0.35 | moderate |
|  | Narcissistic rage | NL | 266,236 | 40,658 | 306,894 | 156.15 | 74.08 | 130.31 | 23.79 | 9.51 | 0.00 | 0.21 | small |
|  | Narcissistic rage | PL | 224,265 | 54,048 | 278,313 | 131.53 | 45.60 | 173.23 | 37.98 | -16.92 | 0.00 | 0.38 | moderate |
|  | Narcissistic rage | PT | 289,410 | 79,594 | 369,004 | 169.74 | 69.07 | 255.11 | 49.11 | -20.43 | 0.00 | 0.45 | moderate |
|  | Narcissistic rage | RU | 349,512 | 149,772 | 499,284 | 204.99 | 75.36 | 480.04 | 110.63 | -27.16 | 0.00 | 0.60 | large |
|  | Narcissistic rage | SV | 1,193,403 | 113,355 | 1,306,758 | 699.94 | 695.29 | 363.32 | 220.89 | 23.50 | 0.00 | 0.52 | large |
| **19** | Persecutory delusions | EN | 119,673 | 9,802 | 129,475 | 70.19 | 123.66 | 31.42 | 7.10 | 26.72 | 0.00 | 0.59 | large |
|  | Persecutory delusions | ES | 128,140 | 45,248 | 173,388 | 75.16 | 49.34 | 145.03 | 105.22 | -19.40 | 0.00 | 0.43 | moderate |
|  | Persecutory delusions | FR | 182,682 | 36,313 | 218,995 | 107.14 | 27.39 | 116.39 | 19.71 | -7.49 | 0.00 | 0.17 | small |
|  | Persecutory delusions | PL | 4,749 | 1,848 | 6,597 | 2.79 | 2.29 | 5.92 | 3.48 | -16.66 | 0.00 | 0.37 | moderate |
|  | Persecutory delusions | PT | 14,315 | 12,115 | 26,430 | 8.40 | 14.04 | 38.83 | 13.07 | -23.84 | 0.00 | 0.59 | large |
|  | Persecutory delusions | RU | 226,924 | 63,137 | 290,061 | 133.09 | 36.40 | 202.36 | 31.45 | -23.69 | 0.00 | 0.53 | large |
| **20** | Classical Adlerian psychology | EN | 110,555 | 4,461 | 115,016 | 64.84 | 25.97 | 14.30 | 5.52 | 26.96 | 0.00 | 0.60 | large |
|  | Classical Adlerian psychology | DE | 137,677 | 37,625 | 175,302 | 80.75 | 45.13 | 120.59 | 22.27 | -21.75 | 0.00 | 0.48 | moderate |
|  | Classical Adlerian psychology | ES | 168,234 | 38,124 | 206,358 | 98.67 | 45.83 | 122.19 | 46.95 | -8.31 | 0.00 | 0.19 | small |
|  | Classical Adlerian psychology | FR | 29,251 | 6,013 | 35,264 | 17.16 | 34.02 | 19.27 | 8.44 | -7.21 | 0.00 | 0.16 | small |
|  | Classical Adlerian psychology | IT | 26,298 | 6,452 | 32,750 | 15.42 | 6.74 | 20.68 | 7.73 | -11.04 | 0.00 | 0.25 | small |
|  | Classical Adlerian psychology | NL | 5,531 | 1,040 | 6,571 | 3.24 | 3.19 | 3.33 | 2.35 | -0.97 | 0.33 | 0.02 | small |
|  | Classical Adlerian psychology | PL | 15,127 | 4,692 | 19,819 | 8.87 | 6.64 | 15.04 | 7.16 | -15.22 | 0.00 | 0.34 | moderate |
|  | Classical Adlerian psychology | PT | 17,970 | 4,146 | 22,116 | 10.54 | 6.31 | 13.29 | 6.99 | -7.14 | 0.00 | 0.16 | small |
|  | Classical Adlerian psychology | RU | 60,850 | 13,093 | 73,943 | 35.69 | 15.67 | 41.96 | 21.27 | -4.99 | 0.00 | 0.11 | small |
| **21** | Research methods | EN | 37,947 | 2,335 | 40,282 | 22.26 | 10.24 | 7.48 | 4.17 | 26.36 | 0.00 | 0.59 | large |
| **22** | Forgiveness | EN | 956,913 | 102,155 | 1,059,068 | 561.24 | 118.31 | 327.42 | 65.95 | 26.22 | 0.00 | 0.58 | large |
|  | Forgiveness | DE | 105,629 | 17,554 | 123,183 | 61.95 | 19.98 | 56.26 | 16.07 | 6.27 | 0.00 | 0.14 | small |
|  | Forgiveness | ES | 999,478 | 148,459 | 1,147,937 | 586.20 | 203.54 | 475.83 | 141.58 | 9.52 | 0.00 | 0.21 | small |
|  | Forgiveness | FR | 134,278 | 28,053 | 162,331 | 78.76 | 25.34 | 89.91 | 21.34 | -7.87 | 0.00 | 0.18 | small |
|  | Forgiveness | IT | 109,098 | 14,953 | 124,051 | 63.99 | 24.38 | 47.93 | 17.92 | 12.58 | 0.00 | 0.28 | small |
|  | Forgiveness | NL | 15,483 | 2,990 | 18,473 | 9.08 | 4.50 | 9.58 | 4.58 | -1.75 | 0.08 | 0.04 | small |
|  | Forgiveness | PT | 206,900 | 21,227 | 228,127 | 121.35 | 46.62 | 68.04 | 15.18 | 21.94 | 0.00 | 0.49 | moderate |
|  | Forgiveness | RU | 72,120 | 17,468 | 89,588 | 42.30 | 25.33 | 55.99 | 31.61 | -10.86 | 0.00 | 0.24 | small |
|  | Forgiveness | SV | 25,874 | 3,780 | 29,654 | 15.18 | 8.53 | 12.12 | 5.40 | 6.19 | 0.00 | 0.14 | small |
| **23** | List of important publications in psychology | EN | 79,790 | 5,760 | 85,550 | 46.80 | 18.94 | 18.46 | 9.75 | 26.03 | 0.00 | 0.58 | large |
| **24** | Relationship counseling | EN | 280,564 | 6,006 | 286,570 | 164.55 | 105.70 | 19.25 | 7.90 | 26.52 | 0.00 | 0.59 | large |
|  | Relationship counseling | DE | 101,809 | 13,139 | 114,948 | 59.71 | 19.91 | 42.11 | 11.42 | 16.56 | 0.00 | 0.37 | moderate |
|  | Relationship counseling | ES | 106,872 | 29,063 | 135,935 | 62.68 | 37.66 | 93.15 | 21.70 | -14.15 | 0.00 | 0.32 | moderate |
|  | Relationship counseling | NL | 4,306 | 1,888 | 6,194 | 2.53 | 3.53 | 6.05 | 3.54 | -16.60 | 0.00 | 0.38 | moderate |
| **25** | Adolescence | EN | 3,916,280 | 435,727 | 4,352,007 | 2296.94 | 493.93 | 1396.56 | 206.95 | 26.45 | 0.00 | 0.59 | large |
|  | Adolescence | DE | 660,306 | 106,673 | 766,979 | 387.28 | 106.67 | 341.90 | 60.40 | 8.50 | 0.00 | 0.19 | small |
|  | Adolescence | ES | 6,308,359 | 734,735 | 7,043,094 | 3699.92 | 2064.30 | 2354.92 | 1069.29 | 11.70 | 0.00 | 0.26 | small |
|  | Adolescence | FR | 715,594 | 132,611 | 848,205 | 419.70 | 118.88 | 425.04 | 87.18 | -0.21 | 0.84 | 0.00 | small |
|  | Adolescence | IT | 713,068 | 103,422 | 816,490 | 418.22 | 151.13 | 331.48 | 115.73 | 9.53 | 0.00 | 0.21 | small |
|  | Adolescence | NL | 254,764 | 53,213 | 307,977 | 149.42 | 50.20 | 170.55 | 45.73 | -7.55 | 0.00 | 0.17 | small |
|  | Adolescence | PL | 238,674 | 47,266 | 285,940 | 139.98 | 36.55 | 151.49 | 58.82 | -1.38 | 0.17 | 0.03 | small |
|  | Adolescence | PT | 1,231,575 | 179,486 | 1,411,061 | 722.33 | 244.01 | 575.28 | 143.71 | 10.33 | 0.00 | 0.23 | small |
|  | Adolescence | RU | 866,127 | 201,620 | 1,067,747 | 507.99 | 137.64 | 646.22 | 146.49 | -14.83 | 0.00 | 0.33 | moderate |
|  | Adolescence | SV | 18,982 | 3,357 | 22,339 | 11.13 | 5.72 | 10.76 | 5.16 | 0.91 | 0.36 | 0.02 | small |
| **26** | Anxiety disorder | EN | 4,006,826 | 464,211 | 4,471,037 | 2350.04 | 441.00 | 1487.86 | 211.53 | 26.15 | 0.00 | 0.58 | large |
|  | Anxiety disorder | DE | 1,032,255 | 156,279 | 1,188,534 | 605.43 | 124.38 | 500.89 | 68.57 | 15.25 | 0.00 | 0.34 | moderate |
|  | Anxiety disorder | ES | 1,340,099 | 105,342 | 1,445,441 | 785.98 | 358.38 | 337.63 | 83.22 | 21.44 | 0.00 | 0.48 | moderate |
|  | Anxiety disorder | FR | 218,964 | 35,215 | 254,179 | 128.42 | 27.08 | 112.87 | 21.44 | 9.68 | 0.00 | 0.22 | small |
|  | Anxiety disorder | IT | 76,834 | 27,223 | 104,057 | 45.06 | 43.33 | 87.25 | 15.58 | -15.06 | 0.00 | 0.34 | moderate |
|  | Anxiety disorder | NL | 214,195 | 31,324 | 245,519 | 125.63 | 39.97 | 100.40 | 21.09 | 10.93 | 0.00 | 0.24 | small |
|  | Anxiety disorder | PL | 311,379 | 59,870 | 371,249 | 182.63 | 41.97 | 191.89 | 92.31 | 0.37 | 0.71 | 0.01 | small |
|  | Anxiety disorder | RU | 160,145 | 21,426 | 181,571 | 93.93 | 61.69 | 68.67 | 16.99 | 11.31 | 0.00 | 0.25 | small |
|  | Anxiety disorder | SV | 83,201 | 8,684 | 91,885 | 48.80 | 14.60 | 27.83 | 7.18 | 23.07 | 0.00 | 0.51 | large |
| **27** | Habit (psychology) | EN | 92,720 | 7,638 | 100,358 | 54.38 | 19.93 | 24.48 | 11.21 | 25.70 | 0.00 | 0.57 | large |
| **28** | Memory and aging | EN | 158,381 | 9,453 | 167,834 | 92.89 | 42.21 | 30.30 | 14.08 | 26.18 | 0.00 | 0.58 | large |
|  | Memory and aging | NL | 27,579 | 5,614 | 33,193 | 16.18 | 8.94 | 17.99 | 8.06 | -4.73 | 0.00 | 0.11 | small |
| **29** | Endogeny | EN | 99,553 | 3,388 | 102,941 | 58.39 | 26.80 | 10.86 | 5.47 | 26.56 | 0.00 | 0.59 | large |
| **30** | Organizational communication | EN | 547,551 | 39,825 | 587,376 | 321.14 | 143.58 | 127.64 | 51.74 | 26.21 | 0.00 | 0.58 | large |
|  | Organizational communication | DE | 22,877 | 2,912 | 25,789 | 13.42 | 6.34 | 9.33 | 4.48 | 11.07 | 0.00 | 0.25 | small |
|  | Organizational communication | ES | 780,954 | 98,256 | 879,210 | 458.04 | 222.78 | 314.92 | 103.15 | 11.21 | 0.00 | 0.25 | small |
|  | Organizational communication | FR | 54,464 | 9,120 | 63,584 | 31.94 | 14.88 | 29.23 | 16.68 | 3.92 | 0.00 | 0.09 | small |
|  | Organizational communication | IT | 20,527 | 2,716 | 23,243 | 12.04 | 6.68 | 8.71 | 5.11 | 8.98 | 0.00 | 0.20 | small |
|  | Organizational communication | PT | 154,592 | 18,243 | 172,835 | 90.67 | 48.22 | 58.47 | 28.92 | 11.27 | 0.00 | 0.25 | small |
|  | Organizational communication | RU | 1,824 | 1,510 | 3,334 | 1.07 | 2.22 | 4.84 | 3.72 | -20.26 | 0.00 | 0.53 | large |
|  | Organizational communication | SV | 3,708 | 406 | 4,114 | 2.17 | 2.17 | 1.30 | 1.58 | 6.89 | 0.00 | 0.16 | small |
| **31** | Social psychology (psychology) | EN | 106,880 | 3,889 | 110,769 | 62.69 | 71.24 | 12.46 | 5.45 | 26.48 | 0.00 | 0.59 | large |
| **32** | Passive–aggressive behavior | EN | 354,280 | 9,755 | 364,035 | 207.79 | 115.93 | 31.27 | 8.66 | 26.72 | 0.00 | 0.59 | large |
| **33** | Mental calculation | EN | 1,449,023 | 46,516 | 1,495,539 | 849.87 | 1537.03 | 149.09 | 32.63 | 26.64 | 0.00 | 0.59 | large |
|  | Mental calculation | DE | 36,406 | 5,542 | 41,948 | 21.35 | 17.33 | 17.76 | 8.99 | 6.00 | 0.00 | 0.13 | small |
|  | Mental calculation | ES | 309,641 | 80,786 | 390,427 | 181.61 | 94.79 | 258.93 | 279.19 | -4.71 | 0.00 | 0.10 | small |
|  | Mental calculation | FR | 34,294 | 5,851 | 40,145 | 20.11 | 9.15 | 18.75 | 9.08 | 2.81 | 0.00 | 0.06 | small |
|  | Mental calculation | IT | 5,703 | 1,065 | 6,768 | 3.34 | 5.77 | 3.41 | 2.98 | -2.36 | 0.02 | 0.05 | small |
|  | Mental calculation | NL | 22,555 | 1,996 | 24,551 | 13.23 | 14.61 | 6.40 | 3.88 | 10.16 | 0.00 | 0.23 | small |
|  | Mental calculation | RU | 71,705 | 17,977 | 89,682 | 42.06 | 18.54 | 57.62 | 26.68 | -12.18 | 0.00 | 0.27 | small |
|  | Mental calculation | SV | 3,513 | 433 | 3,946 | 2.06 | 1.89 | 1.39 | 1.80 | 6.78 | 0.00 | 0.15 | small |
| **34** | Norm (sociology) | EN | 260,522 | 23,481 | 284,003 | 152.80 | 70.97 | 75.26 | 26.61 | 25.86 | 0.00 | 0.58 | large |
|  | Norm (sociology) | DE | 309,321 | 50,211 | 359,532 | 181.42 | 58.34 | 160.93 | 52.98 | 5.56 | 0.00 | 0.12 | small |
|  | Norm (sociology) | ES | 2,276,593 | 491,686 | 2,768,279 | 1335.25 | 952.00 | 1575.92 | 857.18 | -5.01 | 0.00 | 0.11 | small |
|  | Norm (sociology) | FR | 174,185 | 32,712 | 206,897 | 102.16 | 45.54 | 104.85 | 45.36 | -0.73 | 0.47 | 0.02 | small |
|  | Norm (sociology) | IT | 42,187 | 11,489 | 53,676 | 24.74 | 16.48 | 36.82 | 24.32 | -8.38 | 0.00 | 0.19 | small |
|  | Norm (sociology) | NL | 12,496 | 2,891 | 15,387 | 7.33 | 5.70 | 9.27 | 5.32 | -6.78 | 0.00 | 0.15 | small |
|  | Norm (sociology) | PL | 156,422 | 55,415 | 211,837 | 91.74 | 166.25 | 177.61 | 151.65 | -10.85 | 0.00 | 0.24 | small |
|  | Norm (sociology) | PT | 54,902 | 9,007 | 63,909 | 32.20 | 20.01 | 28.87 | 15.07 | 1.64 | 0.10 | 0.04 | small |
|  | Norm (sociology) | RU | 475,843 | 78,589 | 554,432 | 279.09 | 168.35 | 251.89 | 140.50 | 2.23 | 0.03 | 0.05 | small |
|  | Norm (sociology) | SV | 163,531 | 25,624 | 189,155 | 95.91 | 57.02 | 82.13 | 48.46 | 3.58 | 0.00 | 0.08 | small |
| **35** | Paranoid personality disorder | EN | 1,533,020 | 168,072 | 1,701,092 | 899.13 | 196.59 | 538.69 | 120.70 | 26.04 | 0.00 | 0.58 | large |
|  | Paranoid personality disorder | DE | 852,106 | 90,174 | 942,280 | 499.77 | 123.15 | 289.02 | 38.68 | 26.62 | 0.00 | 0.59 | large |
|  | Paranoid personality disorder | ES | 398,369 | 23,843 | 422,212 | 233.65 | 169.02 | 76.42 | 18.54 | 23.39 | 0.00 | 0.52 | large |
|  | Paranoid personality disorder | FR | 399,996 | 61,972 | 461,968 | 234.60 | 43.60 | 198.63 | 27.61 | 15.01 | 0.00 | 0.33 | moderate |
|  | Paranoid personality disorder | IT | 206,095 | 30,293 | 236,388 | 120.88 | 30.24 | 97.09 | 33.60 | 14.63 | 0.00 | 0.33 | moderate |
|  | Paranoid personality disorder | NL | 49,966 | 5,089 | 55,055 | 29.31 | 12.05 | 16.31 | 8.40 | 20.32 | 0.00 | 0.45 | moderate |
|  | Paranoid personality disorder | PL | 190,598 | 24,526 | 215,124 | 111.79 | 28.63 | 78.61 | 18.39 | 20.09 | 0.00 | 0.45 | moderate |
|  | Paranoid personality disorder | PT | 180,284 | 24,139 | 204,423 | 105.74 | 30.26 | 77.37 | 25.93 | 16.19 | 0.00 | 0.36 | moderate |
|  | Paranoid personality disorder | RU | 283,587 | 59,450 | 343,037 | 166.33 | 37.24 | 190.54 | 29.11 | -12.37 | 0.00 | 0.28 | small |
|  | Paranoid personality disorder | SV | 131,354 | 16,647 | 148,001 | 77.04 | 20.90 | 53.36 | 17.28 | 17.29 | 0.00 | 0.38 | moderate |
| **36** | Stress (medicine) | EN | 156,281 | 12,896 | 169,177 | 91.66 | 38.44 | 41.33 | 12.54 | 25.98 | 0.00 | 0.58 | large |
|  | Stress (medicine) | DE | 665,683 | 86,144 | 751,827 | 390.43 | 112.86 | 276.10 | 64.90 | 16.93 | 0.00 | 0.38 | moderate |
|  | Stress (medicine) | ES | 1,617,627 | 157,913 | 1,775,540 | 948.75 | 533.03 | 506.13 | 152.56 | 15.15 | 0.00 | 0.34 | moderate |
|  | Stress (medicine) | FR | 436,017 | 44,325 | 480,342 | 255.73 | 766.34 | 142.07 | 40.10 | 20.67 | 0.00 | 0.46 | moderate |
|  | Stress (medicine) | IT | 80,159 | 45,779 | 125,938 | 47.01 | 55.25 | 146.73 | 45.47 | -22.04 | 0.00 | 0.49 | moderate |
|  | Stress (medicine) | NL | 147,246 | 23,444 | 170,690 | 86.36 | 28.51 | 75.14 | 23.72 | 6.24 | 0.00 | 0.14 | small |
|  | Stress (medicine) | PL | 385,538 | 74,000 | 459,538 | 226.12 | 103.96 | 237.18 | 136.46 | -0.19 | 0.85 | 0.00 | small |
|  | Stress (medicine) | PT | 286,098 | 25,761 | 311,859 | 167.80 | 69.06 | 82.57 | 21.39 | 22.92 | 0.00 | 0.51 | large |
|  | Stress (medicine) | RU | 1,014,055 | 245,155 | 1,259,210 | 594.75 | 234.53 | 785.75 | 305.32 | -10.18 | 0.00 | 0.23 | small |
| **37** | Hierarchy of needs | EN | 62,616 | 4,452 | 67,068 | 36.72 | 14.60 | 14.27 | 5.91 | 26.21 | 0.00 | 0.58 | large |
|  | Hierarchy of needs | DE | 2,098,446 | 363,038 | 2,461,484 | 1230.76 | 428.18 | 1163.58 | 392.88 | 2.19 | 0.03 | 0.05 | small |
|  | Hierarchy of needs | ES | 5,227,237 | 987,448 | 6,214,685 | 3065.83 | 1536.19 | 3164.90 | 1251.68 | -2.26 | 0.02 | 0.05 | small |
|  | Hierarchy of needs | FR | 1,720,225 | 374,739 | 2,094,964 | 1008.93 | 694.56 | 1201.09 | 433.07 | -7.72 | 0.00 | 0.17 | small |
|  | Hierarchy of needs | IT | 403,523 | 83,982 | 487,505 | 236.67 | 84.39 | 269.17 | 101.09 | -5.29 | 0.00 | 0.12 | small |
|  | Hierarchy of needs | NL | 526,395 | 107,406 | 633,801 | 308.74 | 111.99 | 344.25 | 123.91 | -4.70 | 0.00 | 0.10 | small |
|  | Hierarchy of needs | PL | 563,600 | 158,072 | 721,672 | 330.56 | 137.56 | 506.64 | 258.70 | -12.86 | 0.00 | 0.29 | small |
|  | Hierarchy of needs | PT | 1,080,900 | 274,926 | 1,355,826 | 633.96 | 283.64 | 881.17 | 317.47 | -12.48 | 0.00 | 0.28 | small |
|  | Hierarchy of needs | RU | 2,214,040 | 402,437 | 2,616,477 | 1298.56 | 440.67 | 1289.86 | 386.80 | -0.47 | 0.64 | 0.01 | small |
|  | Hierarchy of needs | SV | 159,898 | 33,298 | 193,196 | 93.78 | 50.22 | 106.72 | 51.72 | -4.43 | 0.00 | 0.10 | small |
| **38** | Amnesia | EN | 2,558,293 | 273,733 | 2,832,026 | 1500.47 | 435.15 | 877.35 | 388.02 | 26.04 | 0.00 | 0.58 | large |
|  | Amnesia | DE | 587,833 | 65,571 | 653,404 | 344.77 | 115.38 | 210.16 | 32.31 | 23.99 | 0.00 | 0.53 | large |
|  | Amnesia | ES | 874,700 | 56,215 | 930,915 | 513.02 | 233.54 | 180.18 | 48.47 | 24.20 | 0.00 | 0.54 | large |
|  | Amnesia | FR | 314,897 | 35,206 | 350,103 | 184.69 | 51.49 | 112.84 | 24.83 | 22.62 | 0.00 | 0.50 | large |
|  | Amnesia | IT | 225,678 | 37,235 | 262,913 | 132.36 | 42.86 | 119.34 | 34.75 | 4.70 | 0.00 | 0.10 | small |
|  | Amnesia | NL | 126,212 | 21,235 | 147,447 | 74.02 | 21.19 | 68.06 | 17.61 | 4.91 | 0.00 | 0.11 | small |
|  | Amnesia | PL | 100,568 | 15,672 | 116,240 | 58.98 | 15.50 | 50.23 | 12.21 | 9.71 | 0.00 | 0.22 | small |
|  | Amnesia | PT | 304,492 | 37,530 | 342,022 | 178.59 | 53.84 | 120.29 | 36.30 | 17.58 | 0.00 | 0.39 | moderate |
|  | Amnesia | RU | 548,043 | 119,350 | 667,393 | 321.43 | 61.89 | 382.53 | 59.47 | -15.02 | 0.00 | 0.33 | moderate |
|  | Amnesia | SV | 109,243 | 18,627 | 127,870 | 64.07 | 20.11 | 59.70 | 22.71 | 4.73 | 0.00 | 0.11 | small |
| **39** | Münchausen syndrome | EN | 419,121 | 14,295 | 433,416 | 245.82 | 188.89 | 45.82 | 9.65 | 26.34 | 0.00 | 0.59 | large |
| **40** | Ego | EN | 772,573 | 62,802 | 835,375 | 453.12 | 166.10 | 201.29 | 37.23 | 26.16 | 0.00 | 0.58 | large |
|  | Ego | DE | 217,352 | 38,802 | 256,154 | 127.48 | 21.89 | 124.37 | 21.89 | 2.62 | 0.01 | 0.06 | small |
|  | Ego | ES | 2,523 | 2,978 | 5,501 | 1.48 | 4.16 | 9.54 | 5.04 | -23.62 | 0.00 | 0.67 | large |
|  | Ego | FR | 972 | 371 | 1,343 | 0.57 | 1.30 | 1.19 | 1.38 | -10.40 | 0.00 | 0.29 | small |
|  | Ego | IT | 37,578 | 3,248 | 40,826 | 22.04 | 15.06 | 10.41 | 5.20 | 15.76 | 0.00 | 0.35 | moderate |
|  | Ego | NL | 8,777 | 991 | 9,768 | 5.15 | 3.26 | 3.18 | 2.40 | 11.15 | 0.00 | 0.25 | small |
|  | Ego | PL | 4,412 | 814 | 5,226 | 2.59 | 2.04 | 2.61 | 2.10 | 0.08 | 0.94 | 0.00 | small |
|  | Ego | PT | 6,352 | 555 | 6,907 | 3.73 | 4.12 | 1.78 | 1.67 | 11.12 | 0.00 | 0.25 | small |
|  | Ego | RU | 9,540 | 2,022 | 11,562 | 5.60 | 4.70 | 6.48 | 3.13 | -4.78 | 0.00 | 0.11 | small |
|  | Ego | SV | 3,840 | 320 | 4,160 | 2.25 | 3.51 | 1.03 | 1.13 | 8.34 | 0.00 | 0.19 | small |
| **41** | Keirsey Temperament Sorter | EN | 568,849 | 47,050 | 615,899 | 333.64 | 99.78 | 150.80 | 25.01 | 25.97 | 0.00 | 0.58 | large |
|  | Keirsey Temperament Sorter | DE | 49,308 | 7,227 | 56,535 | 28.92 | 9.79 | 23.16 | 10.12 | 11.46 | 0.00 | 0.26 | small |
|  | Keirsey Temperament Sorter | FR | 57,040 | 6,318 | 63,358 | 33.45 | 17.81 | 20.25 | 8.66 | 15.28 | 0.00 | 0.34 | moderate |
|  | Keirsey Temperament Sorter | IT | 10,658 | 2,709 | 13,367 | 6.25 | 5.27 | 8.68 | 6.93 | -7.21 | 0.00 | 0.16 | small |
|  | Keirsey Temperament Sorter | NL | 3,648 | 324 | 3,972 | 2.14 | 2.11 | 1.04 | 1.26 | 9.47 | 0.00 | 0.22 | small |
| **42** | Classical Adlerian psychotherapy | EN | 39,594 | 2,035 | 41,629 | 23.22 | 10.12 | 6.52 | 2.98 | 26.14 | 0.00 | 0.58 | large |
|  | Classical Adlerian psychotherapy | DE | 137,677 | 37,625 | 175,302 | 80.75 | 45.13 | 120.59 | 22.27 | -21.75 | 0.00 | 0.48 | moderate |
|  | Classical Adlerian psychotherapy | ES | 168,234 | 38,124 | 206,358 | 98.67 | 45.83 | 122.19 | 46.95 | -8.31 | 0.00 | 0.19 | small |
|  | Classical Adlerian psychotherapy | FR | 29,251 | 6,013 | 35,264 | 17.16 | 34.02 | 19.27 | 8.44 | -7.21 | 0.00 | 0.16 | small |
|  | Classical Adlerian psychotherapy | IT | 26,298 | 6,452 | 32,750 | 15.42 | 6.74 | 20.68 | 7.73 | -11.04 | 0.00 | 0.25 | small |
|  | Classical Adlerian psychotherapy | NL | 5,531 | 1,040 | 6,571 | 3.24 | 3.19 | 3.33 | 2.35 | -0.97 | 0.33 | 0.02 | small |
|  | Classical Adlerian psychotherapy | PL | 15,127 | 4,692 | 19,819 | 8.87 | 6.64 | 15.04 | 7.16 | -15.22 | 0.00 | 0.34 | moderate |
|  | Classical Adlerian psychotherapy | PT | 17,970 | 4,146 | 22,116 | 10.54 | 6.31 | 13.29 | 6.99 | -7.14 | 0.00 | 0.16 | small |
|  | Classical Adlerian psychotherapy | RU | 60,850 | 13,093 | 73,943 | 35.69 | 15.67 | 41.96 | 21.27 | -4.99 | 0.00 | 0.11 | small |
| **43** | Mentoring | EN | 50,396 | 3,129 | 53,525 | 29.56 | 20.28 | 10.03 | 5.63 | 25.33 | 0.00 | 0.56 | large |
| **44** | Learning theory (education) | EN | 1,654,214 | 163,129 | 1,817,343 | 970.21 | 251.96 | 522.85 | 112.14 | 25.30 | 0.00 | 0.56 | large |
|  | Learning theory (education) | DE | 225,561 | 30,340 | 255,901 | 132.29 | 44.72 | 97.24 | 32.54 | 13.04 | 0.00 | 0.29 | small |
|  | Learning theory (education) | ES | 1,215,537 | 206,205 | 1,421,742 | 712.92 | 319.83 | 660.91 | 197.87 | 2.29 | 0.02 | 0.05 | small |
|  | Learning theory (education) | FR | 9,274 | 1,779 | 11,053 | 5.44 | 3.60 | 5.70 | 4.12 | -1.35 | 0.18 | 0.03 | small |
|  | Learning theory (education) | PT | 24,829 | 3,848 | 28,677 | 14.56 | 8.26 | 12.33 | 7.20 | 4.40 | 0.00 | 0.10 | small |
|  | Learning theory (education) | SV | 9,597 | 6,334 | 15,931 | 5.63 | 9.22 | 20.30 | 12.82 | -20.12 | 0.00 | 0.48 | moderate |
| **45** | Intuition (knowledge) | EN | 147,272 | 15,700 | 162,972 | 86.38 | 20.37 | 50.32 | 15.42 | 25.20 | 0.00 | 0.56 | large |
| **46** | Binge drinking | EN | 1,063,282 | 133,836 | 1,197,118 | 623.63 | 140.21 | 428.96 | 209.98 | 25.37 | 0.00 | 0.56 | large |
|  | Binge drinking | DE | 97,820 | 12,877 | 110,697 | 57.37 | 16.31 | 41.27 | 11.29 | 19.26 | 0.00 | 0.43 | moderate |
|  | Binge drinking | ES | 8,756 | 2,548 | 11,304 | 5.14 | 6.57 | 8.17 | 3.66 | -11.34 | 0.00 | 0.26 | small |
|  | Binge drinking | FR | 164,079 | 15,651 | 179,730 | 96.23 | 59.98 | 50.16 | 13.45 | 22.76 | 0.00 | 0.51 | large |
|  | Binge drinking | IT | 93,119 | 10,506 | 103,625 | 54.62 | 60.78 | 33.67 | 23.63 | 13.64 | 0.00 | 0.30 | moderate |
|  | Binge drinking | PT | 1,449 | 508 | 1,957 | 0.85 | 1.70 | 1.63 | 1.54 | -11.15 | 0.00 | 0.29 | small |
|  | Binge drinking | RU | 107,873 | 21,031 | 128,904 | 63.27 | 37.66 | 67.41 | 16.86 | -7.60 | 0.00 | 0.17 | small |
| **47** | Dysphoria | EN | 2,022,543 | 258,983 | 2,281,526 | 1186.24 | 213.96 | 830.07 | 100.47 | 25.64 | 0.00 | 0.57 | large |
|  | Dysphoria | DE | 320,133 | 57,432 | 377,565 | 187.76 | 41.79 | 184.08 | 31.66 | 1.09 | 0.28 | 0.02 | small |
|  | Dysphoria | ES | 550,756 | 96,490 | 647,246 | 323.02 | 190.98 | 309.26 | 65.65 | -2.54 | 0.01 | 0.06 | small |
|  | Dysphoria | FR | 499,440 | 146,110 | 645,550 | 292.93 | 170.65 | 468.30 | 175.79 | -18.69 | 0.00 | 0.42 | moderate |
|  | Dysphoria | IT | 412,364 | 73,498 | 485,862 | 241.86 | 88.54 | 235.57 | 72.92 | 1.29 | 0.20 | 0.03 | small |
|  | Dysphoria | NL | 42,111 | 7,450 | 49,561 | 24.70 | 15.18 | 23.88 | 8.46 | 0.08 | 0.93 | 0.00 | small |
|  | Dysphoria | PL | 240,151 | 57,828 | 297,979 | 140.85 | 48.53 | 185.35 | 38.95 | -18.16 | 0.00 | 0.40 | moderate |
|  | Dysphoria | PT | 123,909 | 21,287 | 145,196 | 72.67 | 22.13 | 68.23 | 16.43 | 3.67 | 0.00 | 0.08 | small |
|  | Dysphoria | RU | 580,455 | 109,411 | 689,866 | 340.44 | 128.04 | 350.68 | 53.99 | -6.57 | 0.00 | 0.15 | small |
|  | Dysphoria | SV | 61,378 | 9,669 | 71,047 | 36.00 | 23.97 | 30.99 | 22.26 | 6.76 | 0.00 | 0.15 | small |
| **48** | Deterrence (psychology) | EN | 55,482 | 2,633 | 58,115 | 32.54 | 17.65 | 8.44 | 4.22 | 25.83 | 0.00 | 0.58 | large |
| **49** | Reasoning | EN | 221,795 | 19,802 | 241,597 | 130.09 | 90.74 | 63.47 | 21.50 | 24.62 | 0.00 | 0.55 | large |
| **50** | Artisan temperament | EN | 89,159 | 7,822 | 96,981 | 52.29 | 15.64 | 25.07 | 7.17 | 25.60 | 0.00 | 0.57 | large |
|  | Artisan temperament | DE | 49,308 | 7,227 | 56,535 | 28.92 | 9.79 | 23.16 | 10.12 | 11.46 | 0.00 | 0.26 | small |
|  | Artisan temperament | FR | 57,040 | 6,318 | 63,358 | 33.45 | 17.81 | 20.25 | 8.66 | 15.28 | 0.00 | 0.34 | moderate |
|  | Artisan temperament | IT | 10,658 | 2,709 | 13,367 | 6.25 | 5.27 | 8.68 | 6.93 | -7.21 | 0.00 | 0.16 | small |
|  | Artisan temperament | NL | 3,648 | 324 | 3,972 | 2.14 | 2.11 | 1.04 | 1.26 | 9.47 | 0.00 | 0.22 | small |
| ***** | Claustrophobia | EN | 1,376,225 | 192,901 | 1,569,126 | 807.17 | 159.64 | 618.27 | 187.11 | 19.19 | 0.00 | 0.43 | moderate |
|  | Claustrophobia | DE | 515,175 | 65,861 | 581,036 | 302.16 | 864.28 | 211.09 | 86.74 | 18.40 | 0.00 | 0.41 | moderate |
|  | Claustrophobia | ES | 478,019 | 57,289 | 535,308 | 280.36 | 70.78 | 183.62 | 67.37 | 19.61 | 0.00 | 0.44 | moderate |
|  | Claustrophobia | FR | 233,682 | 30,880 | 264,562 | 137.06 | 36.65 | 98.97 | 26.44 | 17.77 | 0.00 | 0.40 | moderate |
|  | Claustrophobia | IT | 77,647 | 10,767 | 88,414 | 45.54 | 19.50 | 34.51 | 10.42 | 13.38 | 0.00 | 0.30 | small |
|  | Claustrophobia | NL | 44,275 | 8,638 | 52,913 | 25.97 | 10.13 | 27.69 | 9.02 | -3.54 | 0.00 | 0.08 | small |
|  | Claustrophobia | PL | 80,723 | 8,305 | 89,028 | 47.34 | 16.09 | 26.62 | 8.01 | 20.90 | 0.00 | 0.47 | moderate |
|  | Claustrophobia | PT | 133,198 | 11,828 | 145,026 | 78.12 | 32.14 | 37.91 | 22.55 | 23.95 | 0.00 | 0.53 | large |
|  | Claustrophobia | RU | 393,731 | 93,969 | 487,700 | 230.93 | 52.76 | 301.18 | 71.06 | -18.35 | 0.00 | 0.41 | moderate |
|  | Claustrophobia | SV | 36,719 | 3,509 | 40,228 | 21.54 | 7.84 | 11.25 | 5.59 | 22.17 | 0.00 | 0.49 | moderate |

Below we are presenting the graphs which were used for analysis of our results.

**Figure 12**

*2016-2021 Views of “Social Distance” Article in English Wikipedia*


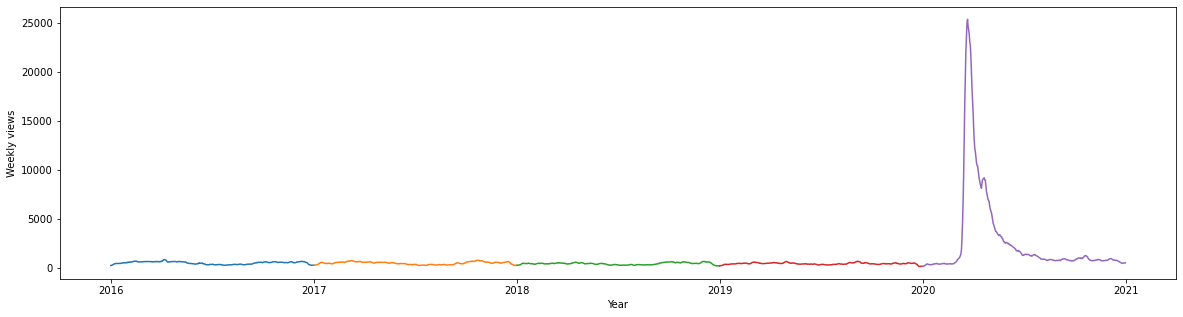


**Figure 13**

*Annual Sums of Views of “Social Distance” Article in seven Wikipedias*


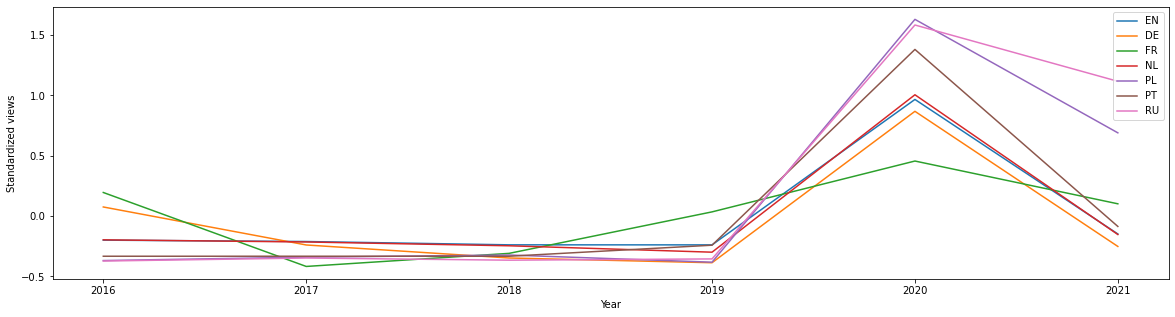


“Comorbidity” on English Wikipedia has the 11th highest increase of interest in the pandemic, scoring -24.95 in the Wilcoxon’s test with 0.56 effect size. It had two major peaks in March 2020 and in September 2020 with a significant drop during the summer holidays. Each peak amounted to about 23,000 weekly searches, whereas in the lowest periods between 2016 and 2020 there were around 3,000 views. It can be seen in the graph that during the previous years the trend was similar (slightly more searches in winter and autumn, visible decrease in summer), but after March 2020 the trend is changed. In most other languages - Spanish, French, Italian, Portuguese and Russian - searches rose during the pandemic as well, with the W scores equal to -27.31, -23.21, -10.23, -25.58, and -6.14, and effect size 0.61, 0.52, 0.23, 0.57,and 0.14 respectively. In German and Dutch they fell quite significantly (W= 22.56 and 7.12, effect size = 0.50 and 0.16) with no change in the pattern of searches between 2016-2020. In Swedish the searches also decreased (W= 9.46, effect size = 0.21), but it is interesting to note that in March 2020 there was a peak reaching up to 1600 weekly searches (in comparison to 200-400 weekly searches in all other periods). In Polish, there was no entry in Wikipedia corresponding to comorbidity.

**Figure 14:**

*2016-2021 Weekly Views of “Comorbidity” Article in English Wikipedia*


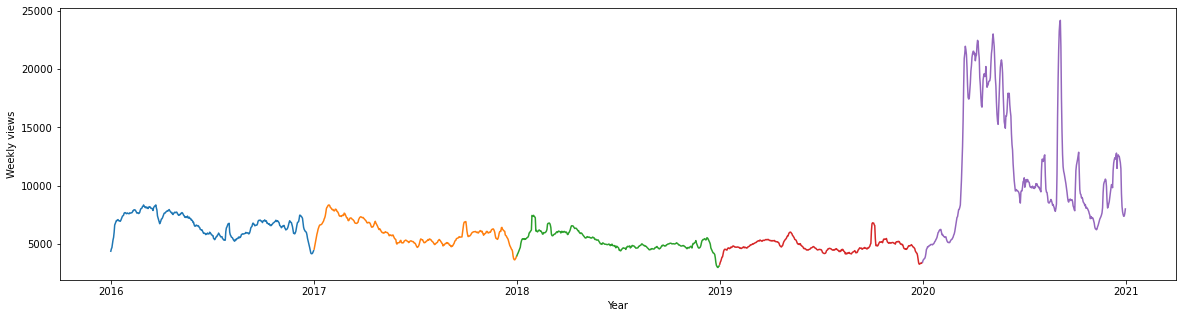


**Figure 15**

*Annual sums of views of “Comorbidity” article in nine Wikipedias*


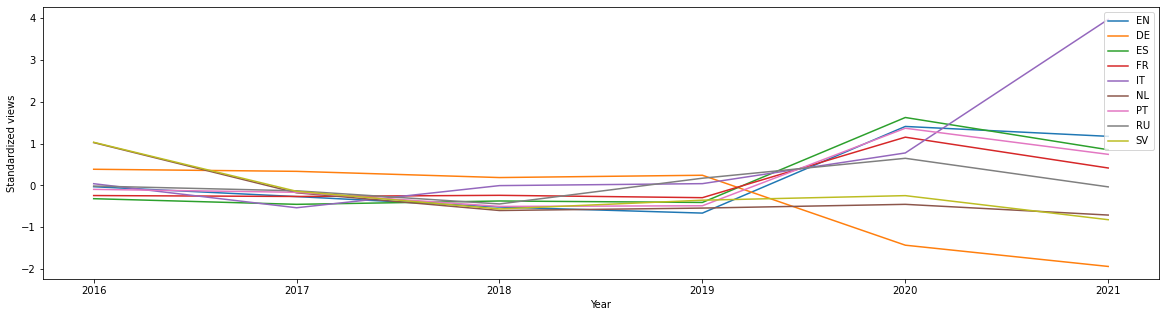


“Neurocognition” is the topic which has the lowest W score on English Wikipedia in our database (-27.87), effect size = 0.66, which means that there were a lot more searches in the pandemic than before. But, besides the English entry, it is only present in German, where the situation is much different. While the web traffic in English was almost nonexistent in the years 2016-2020, in the middle of January 2020 it rose to around 100 searches a week and later to almost 300 in the middle of May, where it stayed, with some plunges to 200 searches, until the end of the period we analyzed. In German, however, the article was visited less often each year with a rather uniform trend of small dips and peaks with a slightly more interest at the beginning and end of each year.

**Figure 16**

*2016-2020 Standardized Monthly Sums of Views of “Neurocognition” Article in two Wikipedias*


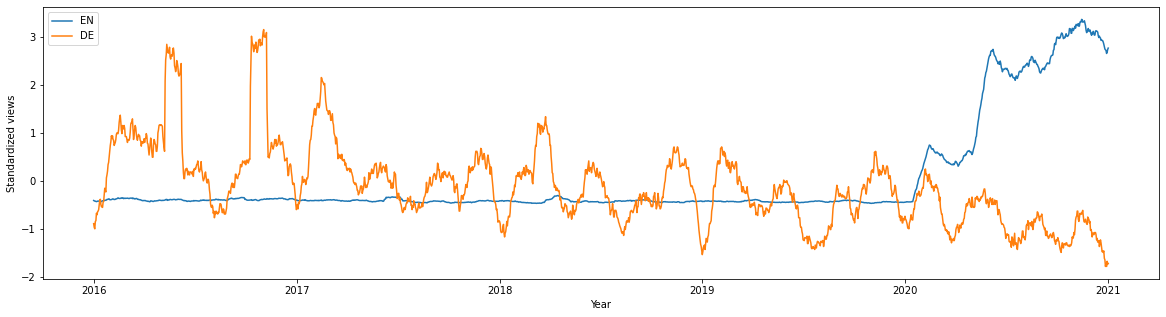


**Figure 17**

*2016-2021 Views of “Neurocognition” Article in English Wikipedia*


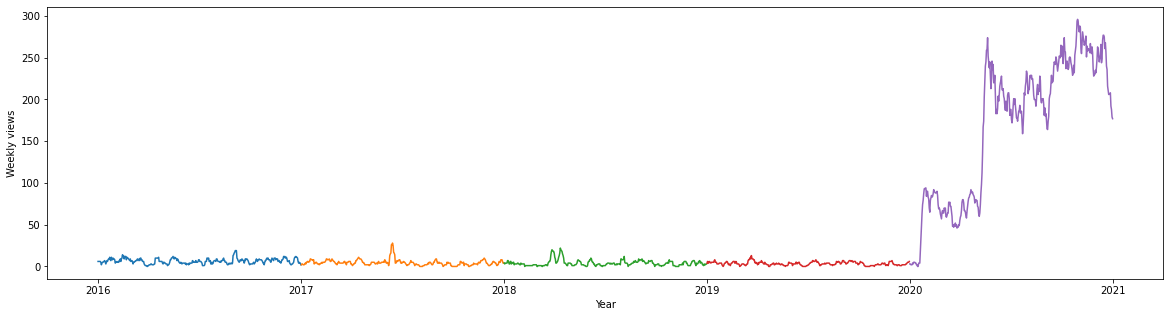


**Figure 18**

*Annual Sums of Views of “Neurocognition” Article in two Wikipedias*


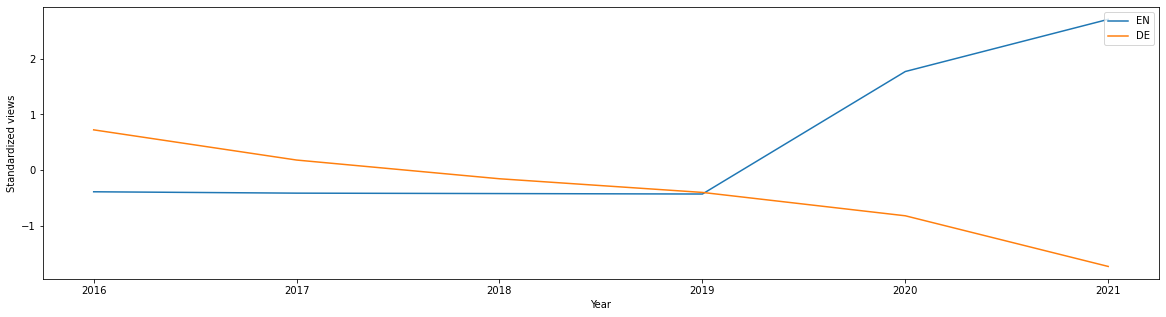


**Figure 19**

*2016-2021 Views of “Psychological pain” Article in English Wikipedia*


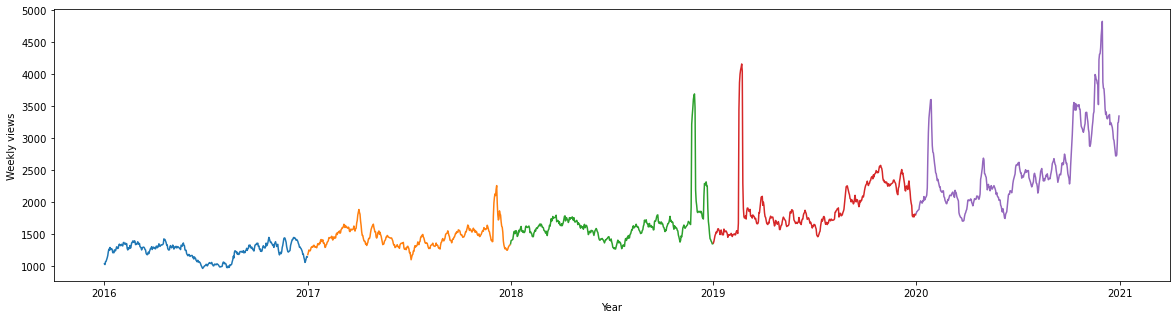


**Figure 20**

Annual Sums of Views of “*Psychological pain”* Article in two Wikipedias


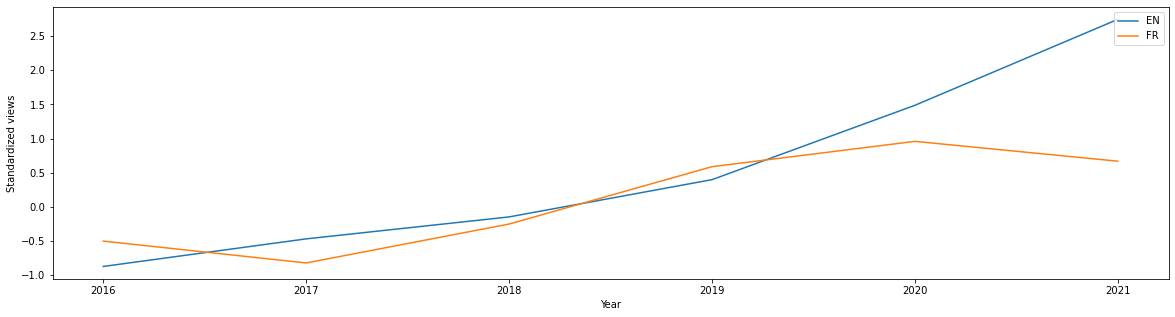


**Figure 21**

*2016-2021 Views of “Defence mechanism” Article in English Wikipedia*


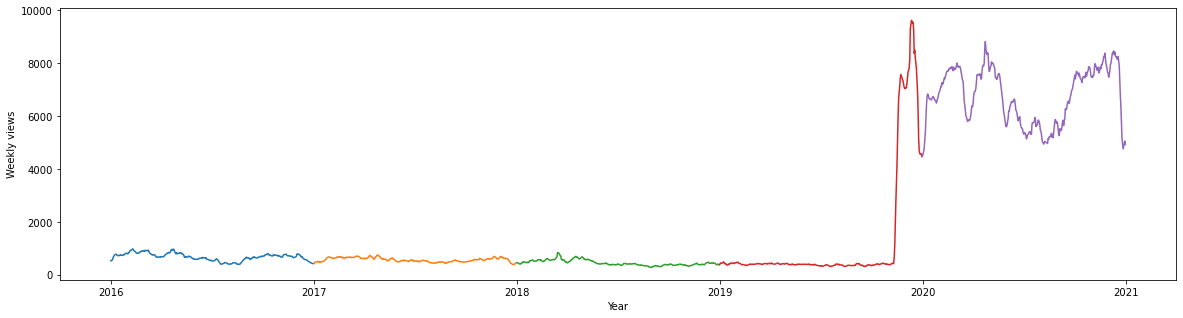


“Introjection” in English Wikipedia became more popular (W = -23.17, effect size = 0.52) in February 2020 and stayed at that level, repeating the shape of the graphs from previous years, but with more views per week, until almost the end of the year. In German, French and Russian the searches also rose (W = -12.64, -3.85, -20.99, and effect size 0.28, 0.09, and 0.47 respectively) without a change in the trend. In Spanish and Dutch it dropped (W = 11.77, 2.77, and effect size 0.26 and 0.06 respectively). In Italian, Polish and Portuguese the result was not statistically significant (p=0.20, p=0.09 and p=0.25 respectively); in Swedish there is no topic corresponding to “Introjection”.

**Figure 22**

*Standardardized Annual Sums of views of “Introjection” Article in nine Wikipedias*


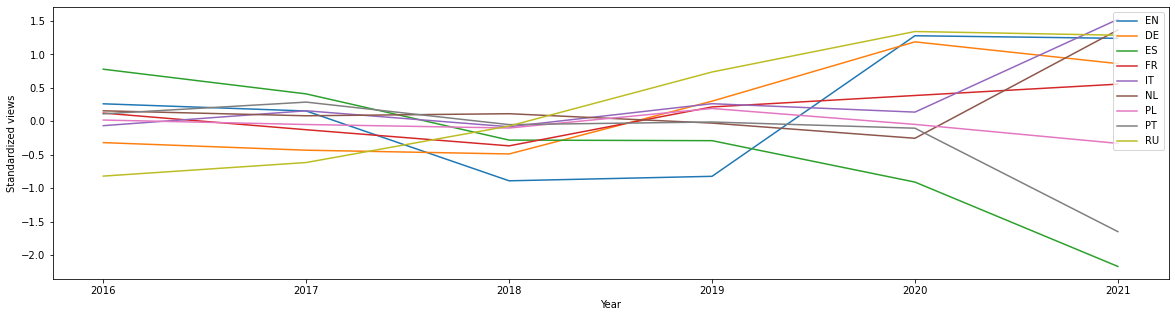


**Figure 23**

*2016-2021 Views of “Introjection” Article on English Wikipedia*


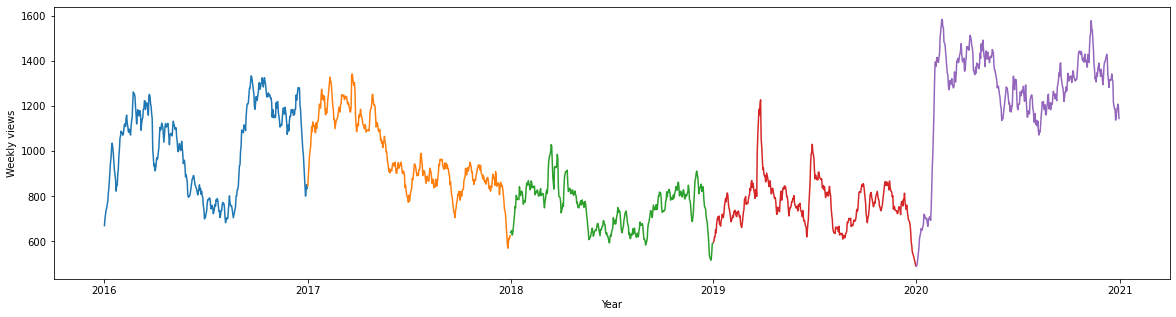


**Figure 24**

*2016-2021 Views of “Introjection” Article in English Wikipedia*


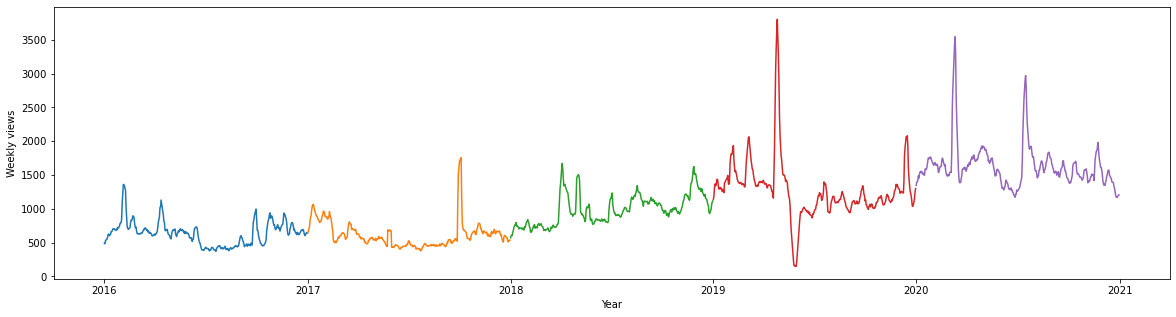


“Breathwork” had the 14th biggest increase in searches in English Wikipedia in our database, with the W score equal to -23.48 and effect size 0.52. In other languages, this article is only present in German with W = -18.80 and effect size 0.42 and a very similar behavior of the graph as the English one throughout all years, especially in 2020. In both languages there was a significant peak around the beginning of the pandemic - in English around the beginning of March to a level of around 3,500 searches a week (whereas the lowest number of searches in 2020 was around 1200 a week), and in German it was 116 a week by the end of March, with the lowest level of interest at 20 a week. The next peak was in July in both languages; the English entry reached the level of 3,000 searches a week, whereas German reached 80.

**Figure 25**

*2016-2021 Standardized Monthly Sums of Views of “Breathwork” Article in two Wikipedias*


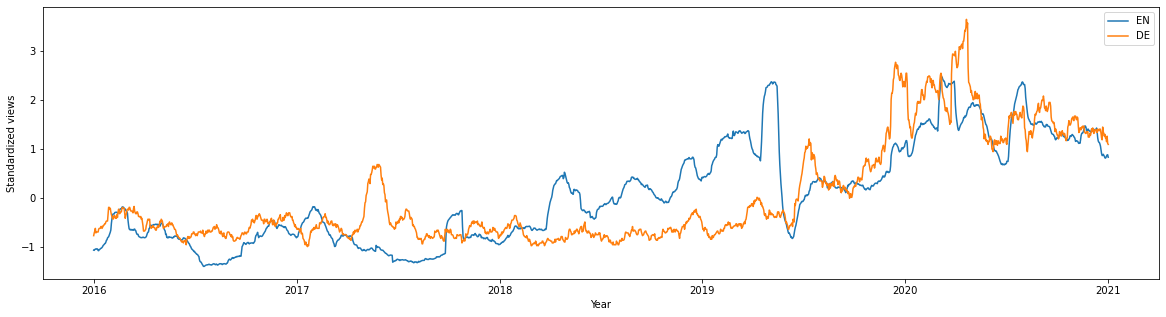


**Figure 26**

*Annual Sums of Views of “Breathwork” Article in two Wikipedias*


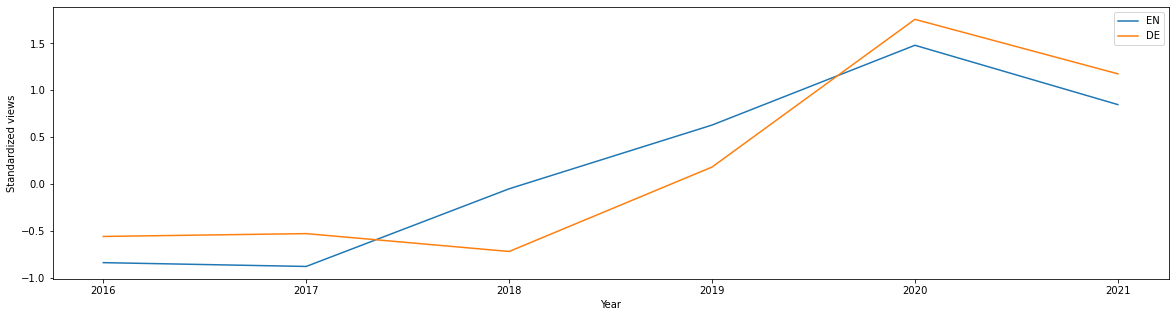


“Logotherapy” has the 24th highest increase of searches in English Wikipedia in the pandemic (W = -21.84 and effect size = 0.49). There is a notable peak at the beginning of April 2020 (7,000 weekly searches), which started to build up in the middle of March. The lowest number of searches in 2020 is around 3,400 a week. What is interesting, not only there is a rise in searches of this topic, but also the peak between March and April 2020 can be seen in almost all other languages we analyzed - German, French, Polish, Portuguese, and Russian (W = -4.48, -9.19, -14.42, -15.70, and -5.11 and effect size = 0.10, 0.20, 0.32, 0.35, and 0.11 respectively). A similar peak at that same period in 2020 can be seen in Italian as well, even though the topic has been less popular during the pandemic (W = 4.62, effect size = 0.10). In Spanish, the change is not statistically significant (p = 0.71). In Dutch and Swedish there is no data. The rest of the graphs in all the languages with the increase in searches behave similarly to the previous years, with a slight decrease during the summer holidays, and then an increase afterwards. In English, however, the trend from previous years did not continue and after the peak in April, there was a small decrease to around 4,900 a week, which stayed at that level until almost the end of the year.

**Figure 27**

*2016-2021 Views of “Logotherapy” Article in English Wikipedia*


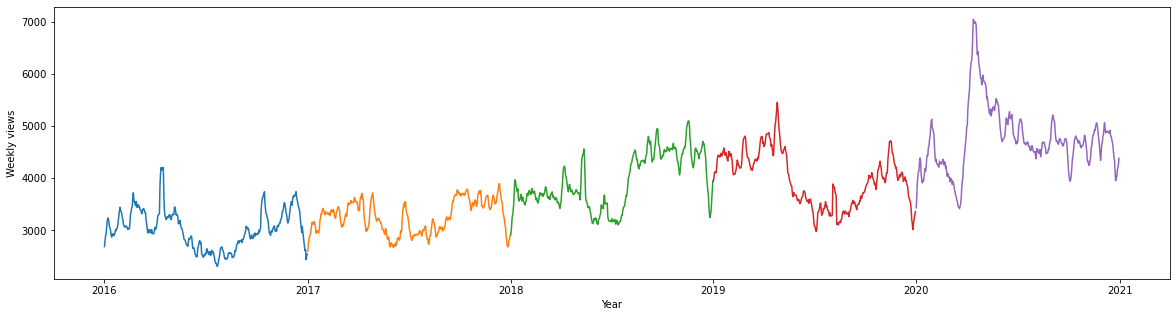


**Figure 28**

*Annual Sums of Views of “Logotherapy” Article in eight Wikipedias*


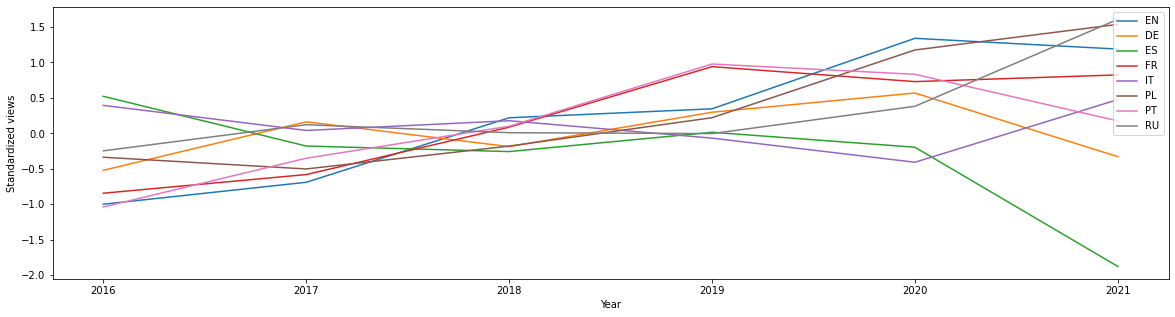


**Figure 29**

*2016-2021 Views of “Substance intoxication” Article in English Wikipedia*

**
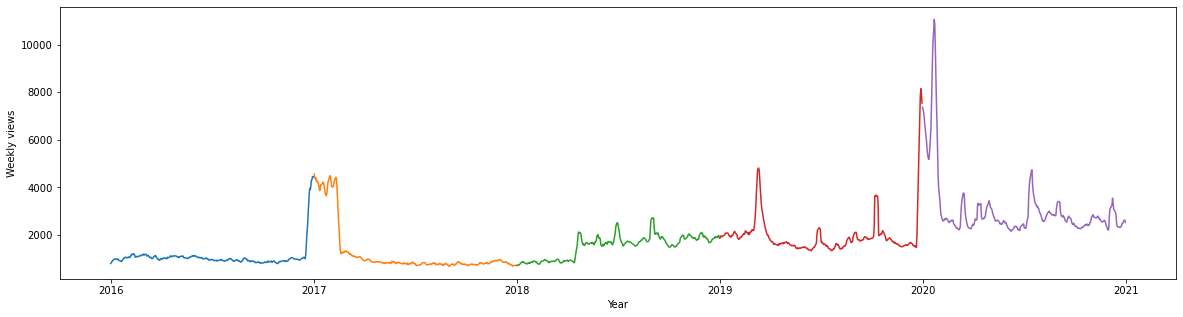
**

**Figure 30**

*2016-2021 Views of “Symbolic violence” Article in English Wikipedia*

**
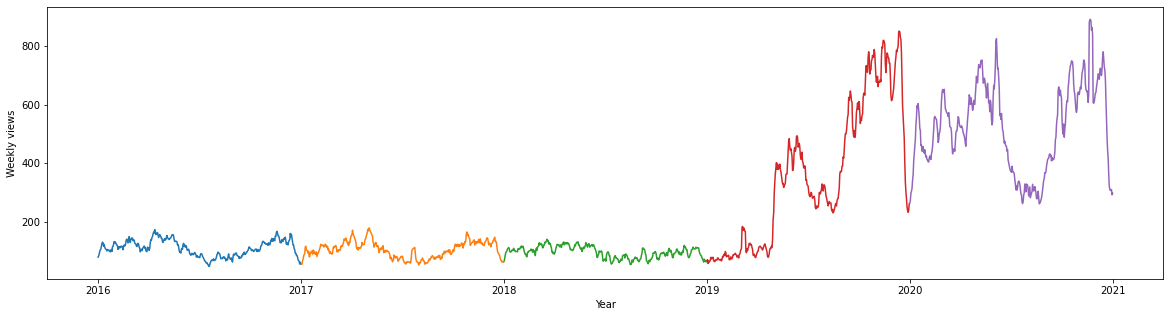
**

**Figure 31**

*Annual Sums of Views of “Symbolic violence” Article in two Wikipedias*


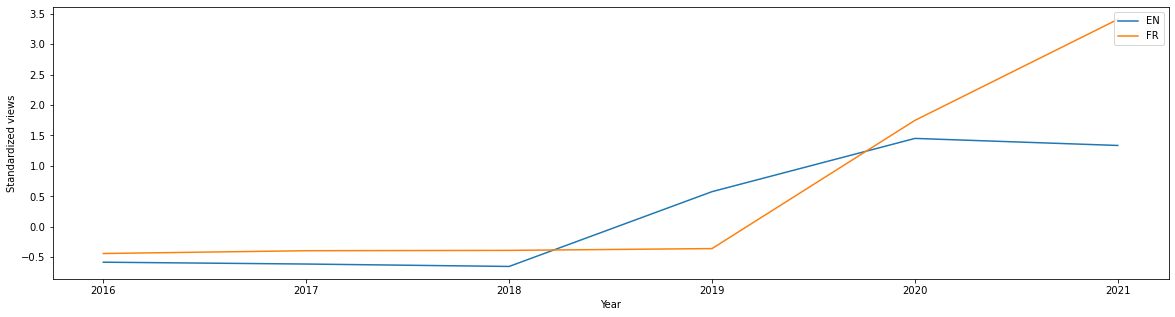


**Figure 32**

*Annual Sums of Views of “Reactance (psychology)” Article in seven Wikipedias*


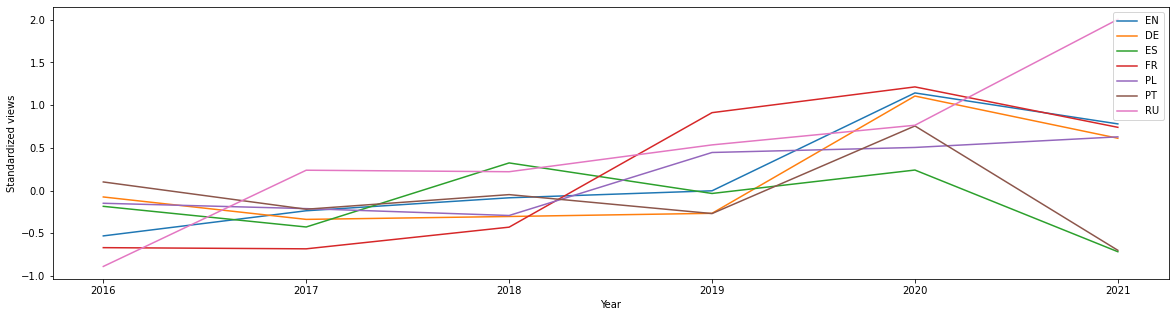


“Righteous indignation” in English Wikipedia is the 38th topic with the highest rise of searches during the pandemic, with the W score equal to -20.17 and the effect size 0.45. It is interesting to note that the first serious peak in 2020 was at the beginning of June (to around 4500 searches a week), which was built up slowly from the middle of March (from 1000 to 1900 searches a week). Another, even higher rise was in the middle of July, to almost 7000 weekly searches. After that, the interest in this topic fell to the level from the beginning of the year, between 1000 and 1500 searches a week. There are no corresponding topics in other languages, but still the trend is different from the period 2016-2019, where we could see a slight decrease of interest from March to September, and then again a slight increase. This seems to be the opposite to what happened in 2020.

**Figure 33**

*2016-2021 Weekly Views of “Righteous indignation” Article in English Wikipedia*


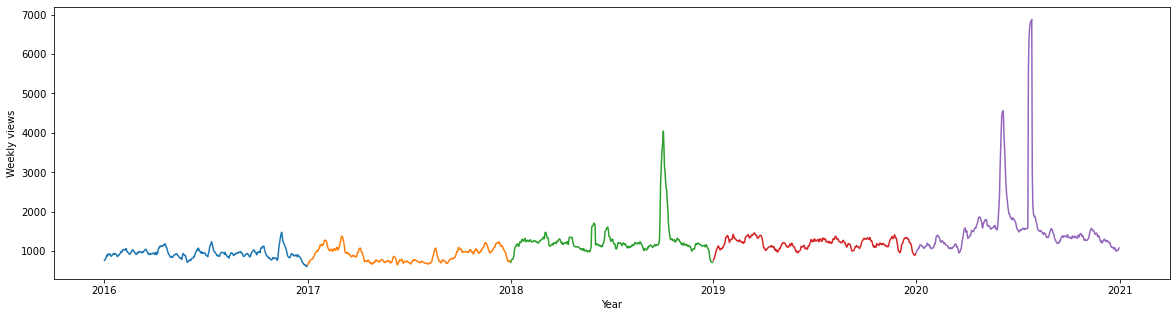


**Figure 34**

*2016-2021 Views of “Levels-of-processing effect” Article in English Wikipedia*


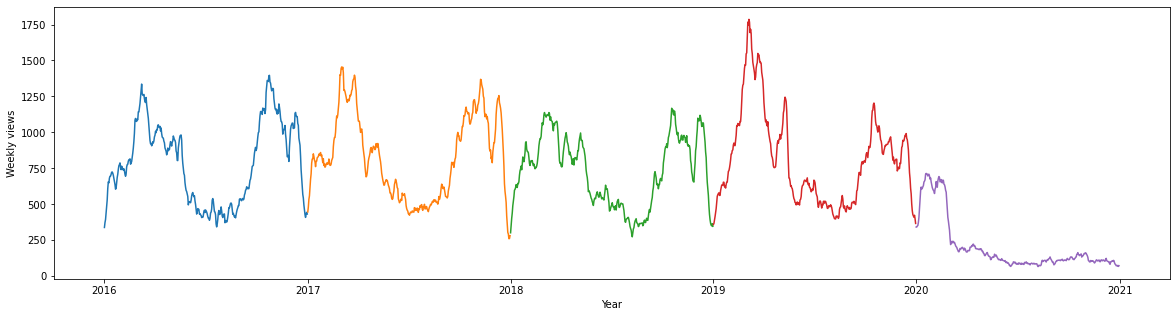


**Figure 35**

*2016-2021 Views of “List of important publications in psychology” Article in English Wikipedia*


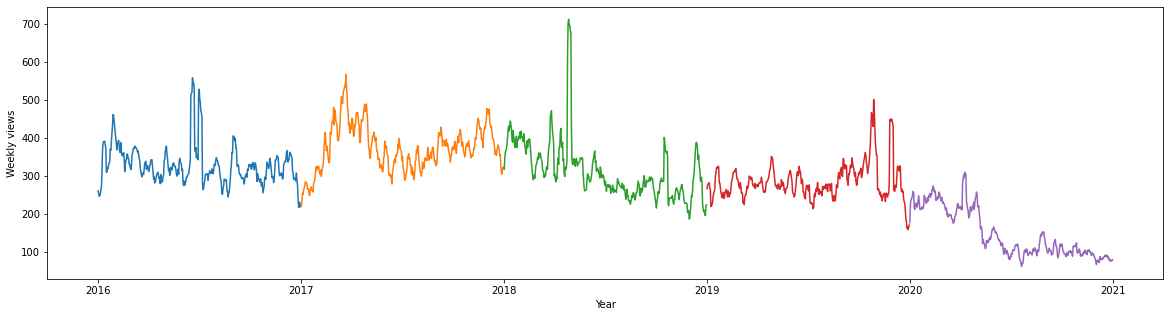


The article “Stir crazy (condition)” had one of the highest W scores (26.72) and effect size 0.60 in English Wikipedia that shows a huge decline in interest during the pandemic. In 2020 there is just a small increase in searches in March. It is also present in Spanish, French, Russian, and Swedish in our database, in which the interest during the pandemic increased (W scores are equal to -8.65, -21.63, -22.48, -13.18 and effect size equal to 0.52, 0.86, 0.53, and 0.29 respectively). In Russian and Swedish, there were peaks of interest around the same place as the minor peak we observed in English, but they were much more prominent. In Russian there was a growth from 200 to 800 searches a week; in Swedish from 250 to 1700 searches a week. After those peaks (around two months), the interest came back to the usual level. It is also important to notice that the topic was not present in these languages from the beginning of the period we analyzed - in Spanish until the end of September 2020; in French until the beginning of May 2020; in Russian until 2018. In English and Swedish, the topic was present for the whole period.

**Figure 36**

*2016-2021 Standardized Monthly Sums of Views of “Stir crazy (condition)” Article in five Wikipedias*


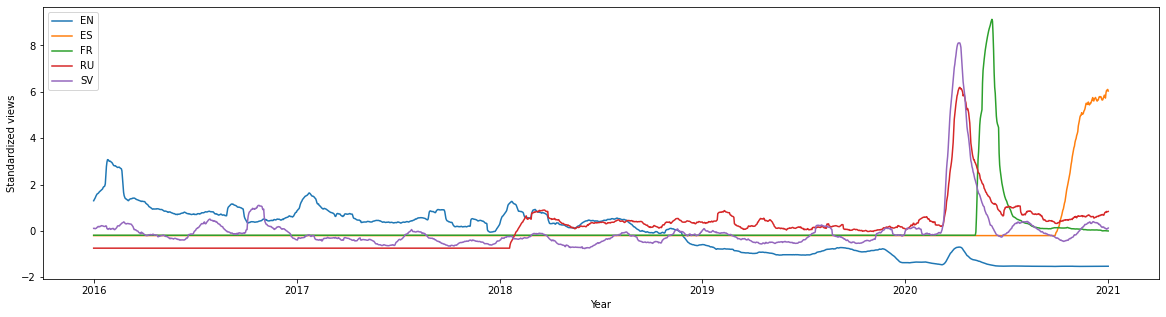


**Figure 37**

*2016-2021 Views of “Stir crazy (condition)” Article in English Wikipedia*


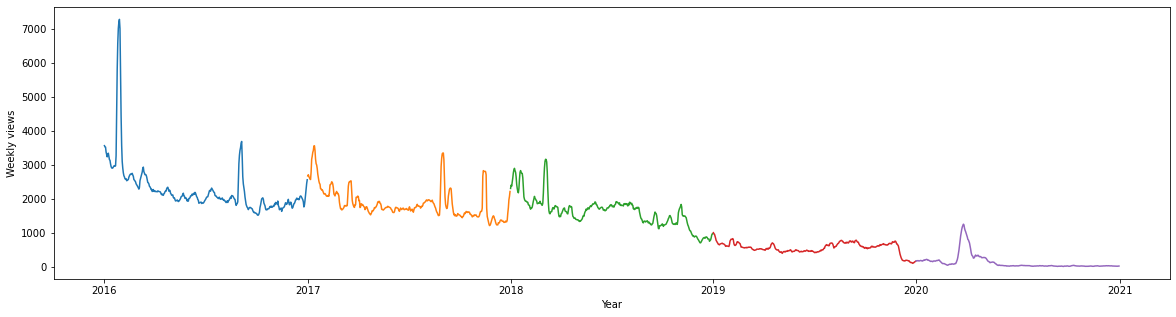


**Figure 38**

Annual Sums of Views of “*Stir crazy (condition)”* Article in five Wikipedias


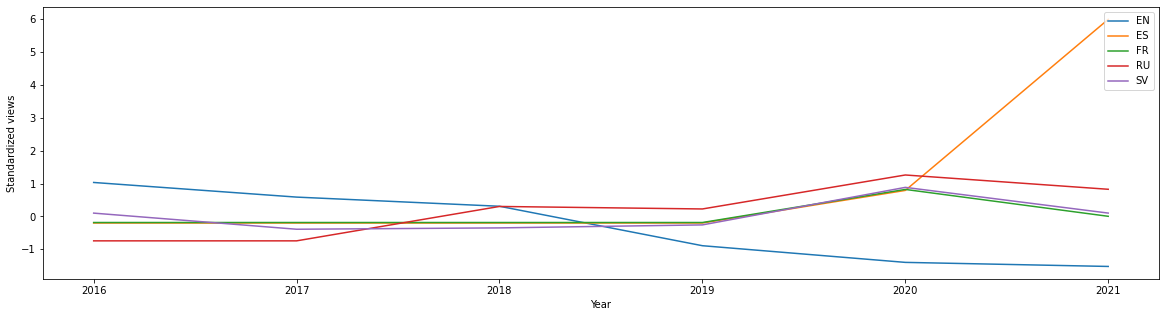


**Figure 39**

*2016-2021 Views of “Claustrophobia” Article in English Wikipedia*


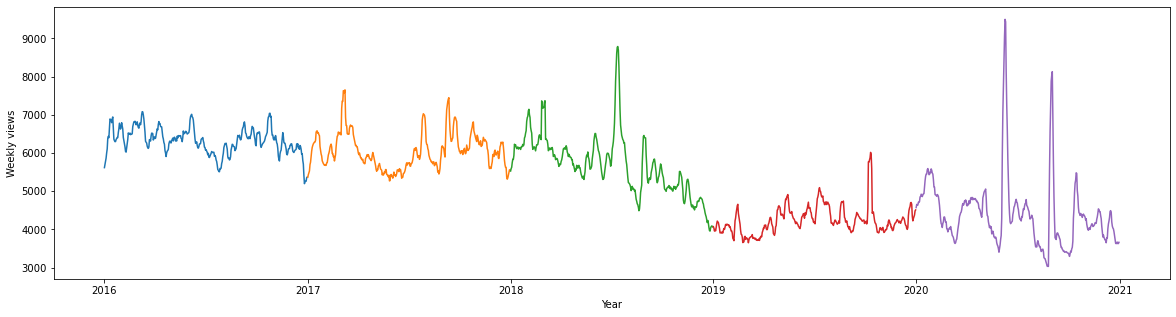


**Figure 40**

*Annual Sums of Views of “Relationship counseling” Article in four Wikipedias*


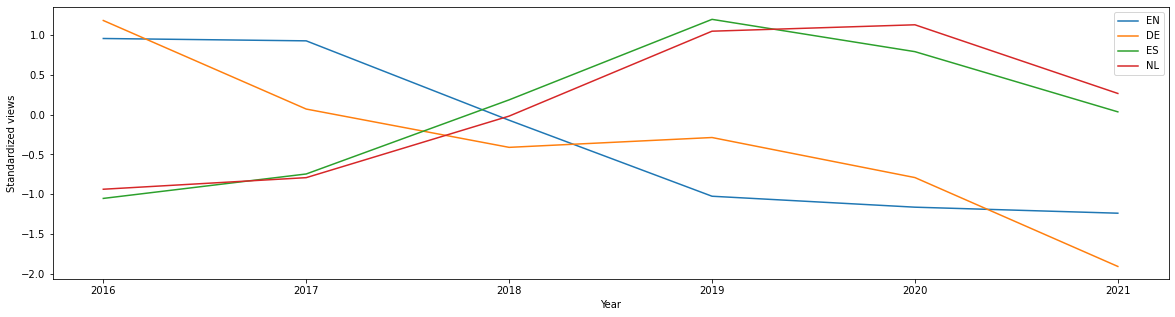


**Figure 41**

*2016-2021 Views of “Relationship counseling” Article in English Wikipedia*


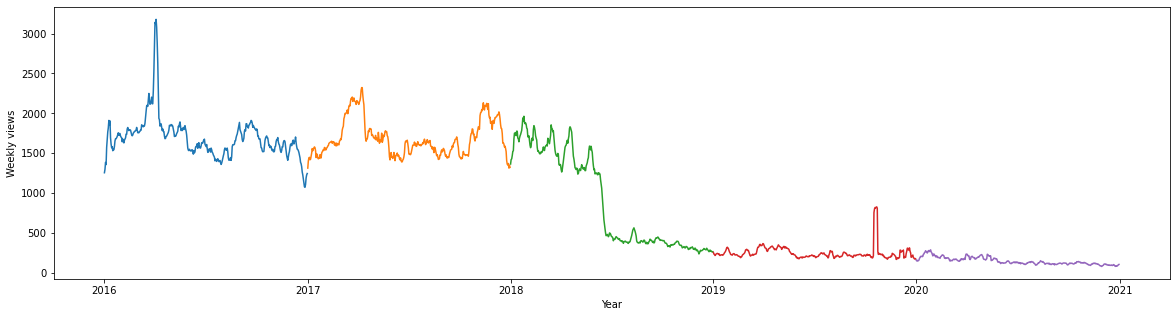


**Figure 42**

*2016-2021 Views of “Stress (medicine)” Article in English Wikipedia*


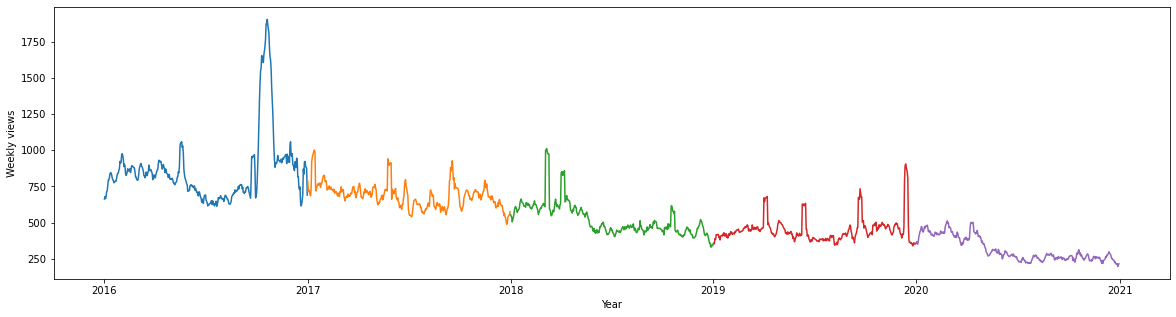


**Figure 43**

*2016-2021 Views of “Burnout (psychology)” Article in English Wikipedia*


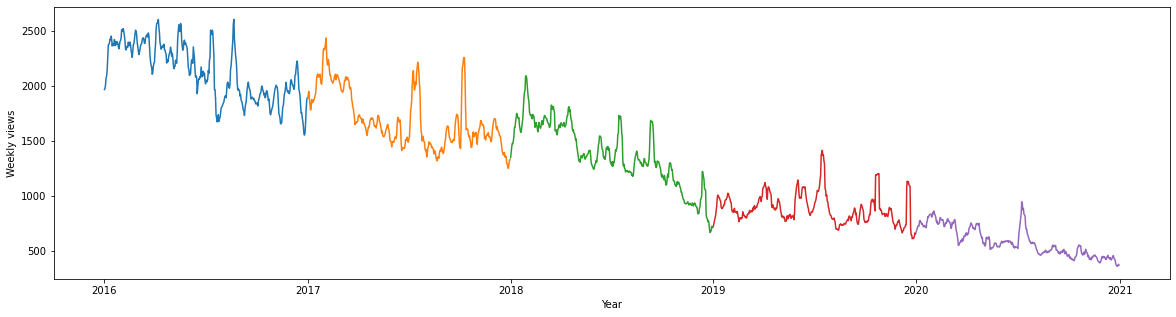


**Appendix 1**

*The Full List of 1763 Articles in Alphabetical Order*

16 Personality Factors

Abnormal psychology

Abreaction

Absexual

Abuse

Acceptance and commitment therapy

Acrophobia

Acting out

Action research

Active intellect

Active learning

Activity theory

Actor-observer bias

Adaptation

Adaptive behavior

Adjustment disorder

Adolescence

Adolescent psychology

Advanced Placement Psychology

Affect (psychology)

Affect display

Affectional bond

Affectional orientation

Affective forecasting

Affective science

Affirming the consequent

Afterburn (psychotherapy)

Age regression

Ageing

Aggression

Agoraphobia

Agraphia

AIDS dementia complex

Akinesia

Alexithymia

Algophobia

Allophilia

Alogia

Alter ego

Altered state of consciousness

Altruism

Alzheimer's disease

Ambivalence

American Psychological Association

Amnesia

Anal retentive

Anal stage

Analytical psychology

Anamnesis (philosophy)

Anchoring

Anger

Anhedonia

Anima and animus

Animal hoarding

Anomic aphasia – Anomie

Anorexia nervosa

Anorgasmia

A-not-B error

Anterograde amnesia

Anticathexis

Anticipation (emotion)

Antidepressant

Antilocution

Antipathy

Antipsychotic

Anti-social behaviour

Anxiety

Anxiety disorder

Anxiogenic

Apathy

Aphanisis

Aphasia

Apoplexy

Apperception

Applied behavior analysis

Applied psychology

Approach-avoidance conflict

Aquaphobia

Archetypal psychology

Archetype

Arousal

Artificial demand

Artisan temperament

Asian psychology

Asperger syndrome

Assertiveness

Association (psychology)

Astraphobia

Attachment disorder

Attachment theory

Attention

Attention seeking

Attention span

Attention-deficit hyperactivity disorder

Attitude (psychology)

Attribution (psychology)

Attribution theory

Attributional bias

Atypical depression

Audience effect

Auditory processing disorder

Aura

Australasian Society for Experimental Psychology

Australian Psychological Society

Autassassinophilia

Authoritarian personality

Authority

Autism

Autism Diagnostic Observation Schedule

Autism spectrum

Autoassociative memory

Autodidacticism

Automatic behavior

Availability heuristic

Aversion therapy

Aversives

Avoidant personality disorder

Avolition

Awareness

Backward inhibition

Baragnosis

Barnes Akathisia Scale

Barnes maze

Beck's cognitive triad

Behavior

Behavior modification

Behavior modification facility

Behavioral communication

Behavioral economics

Behavioral engineering

Behavioral medicine

Behavioral neurology

Behavioral neuroscience

Behavioral theories of depression

Behaviorism

Behaviour therapy

Behavioural despair test

Behavioural genetics

Behavioural sciences

Belief

Belongingness

Beyond the Pleasure Principle

Bibliomania

Bicameralism (psychology)

Bidirectional associative memory

Binge drinking

Binge eating

Biodata

Bioenergetic analysis

Biofeedback

Biological psychology

Bipolar disorder

Bipolar I disorder

Bipolar II disorder

Blame

Block design test

Blood-injection-injury type phobia

Blunted affect

Boanthropy

Body dysmorphic disorder

Body image

Body language

Body psychotherapy

Boldness

Borderline intellectual functioning

Borderline personality disorder

Borromean clinic

Bouma

Bradykinesia

Brainstorming

Breathwork

Brief psychotherapy

Brief reactive psychosis

British Journal of Social Psychology

British Psychological Society

Bulimia nervosa

Bullying

Burn Syndrome

Burnout (psychology)

Bystander effect

California Psychological Inventory

California School of Professional Psychology

Canadian Psychological Association

Cannon-Bard theory

Care perspective

Cassandra (metaphor)

Castration anxiety

Catalepsy

Catastrophization

Catatonia

Catatonic schizophrenia

Category:Clinical psychology tests

Catharsis

Cathexis

Center for Evolutionary Psychology

Centration

Chaining

Character orientation

Character structure

Charisma

Cheating

Chemical imbalance

Child (archetype)

Child abuse

Child and Adolescent Mental Health Services

Child development

Child directed speech

Child sexual abuse

Childhood disintegrative disorder

Chinese Classification of Mental Disorders

Chronophilia

Chunking (psychology)

Cibophobia

Cinderella complex

Cinderella effect

Circadian rhythm

Circadian rhythm sleep disorder

Circumstantiality

Clanging

Classical Adlerian psychology

Classical Adlerian psychotherapy

Classical conditioning

Claustrophobia

Clinical psychology

Clique

Closure (psychology)

Codependency

Cognition

Cognitive appraisal

Cognitive behavioral therapy

Cognitive bias

Cognitive development

Cognitive dimensions of notations

Cognitive disorder

Cognitive dissonance

Cognitive distortion

Cognitive elite

Cognitive evaluation theory

Cognitive interventions

Cognitive load

Cognitive map

Cognitive neuropsychology

Cognitive psychology

Cognitive restructuring

Cognitive revolution

Cognitive science

Cognitive shift

Cognitive slippage

Cognitive space

Cognitive specialization

Cognitive style

Cognitive test

Cognitive therapy

Cognitivism (psychology)

Cohort effect

Collective consciousness

Collective identity

Collective intelligence

Collective unconscious

Color agnosia

Color psychology

Combat stress reaction

Communication disorder

Communication sciences

Community mental health service

Community psychology

Comorbidity

Comparative psychology

Compensation (psychology)

Compersion

Complex (psychology)

Complex post-traumatic stress disorder

Compliance (psychology)

Compulsive behavior

Computational theory of mind

Conceptual blending

Conceptual model

Concrete operational stage

Concurrent validity

Conditioning, operant

Conduct disorder

Confabulation

Confidence

Confirmation bias

Conformity

Congenital disorder

Conscientiousness

Consciousness

Conservation (psychology)

Contact hypothesis

Content validity

Continuous reinforcement

Control freak

Control theory

Convergent thinking

Conversion disorder

Coordination disorder, developmental

Coping (psychology)

Coping skill

Coprophilia

Cotard delusion

Coulrophobia

Counseling psychology

Countersignaling

Creative problem solving

Creativity

Criminal psychology

Critical period

Critical psychology

Critical thinking

Criticism

Cross-sectional study

Crowd manipulation

Crowd psychology

Cryptomnesia

Cue-dependent forgetting

Cultivation theory

Cultural dimensions

Cultural identity

Cultural psychology

Culture shock

Curiosity

Cyberpsychology

Cyclopean image

Cyclothymia

Da Costa's syndrome

Daydream

Dead inside (concept)

Death drive

Decay theory

Deception

Decision analysis

Decision theory

Declarative learning

Declarative memory

Defence mechanism

Deindividuation

Deinstitutionalisation

Déjà vu

Delay reduction hypothesis

Delayed sleep phase syndrome

Delirium

Delusion

Delusion and Dream in Jensen's Gradiva

Delusional disorder

Demand (psychoanalysis)

Demand characteristics

Dementia

Dementia praecox

Denial

Dental fear

Dependency need

Dependent personality disorder

Depersonalization

Depersonalization disorder

Depressive Disorder Not Otherwise Specified

Depressive personality disorder

Depressive position

Depressive realism

Depth psychology

Derealization

Dereistic thinking

Dermatillomania

Desensitization (psychology)

Design thinking

Designated patient

Desire (psychoanalysis)

Destrudo

Detection theory

Deterrence (psychology)

Devaluation

Developmental coordination disorder

Developmental disorder

Developmental lines

Developmental profile

Developmental psychology

Developmental stage theories

Diagnostic and Statistical Manual of Mental Disorders

Dialectical behavior therapy

Dialogical self

Diathesis–stress model

Dichotic listening

Differential psychology

Diogenes syndrome

Dipsomania

Disappointment

Discrimination

Discursive psychology

Disinhibited attachment disorder

Disinhibition

Disintegrative disorder

Disorder of written expression

Disorganized schizophrenia

Displacement (psychology)

Dispositionist

Dissocial personality disorder

Dissociation

Dissociative amnesia

Dissociative disorder

Dissociative fugue

Dissociative identity disorder

Distancing language

Distressed personality type

Distributed cognition

Distrust

Divergent thinking

Doctor of Psychology

Dominance and submission

Door-in-the-face technique

Double bind

Double depression

Dream

Dream dictionary

Dream interpretation

Dream journal

Dream transference

Drive Theory

DSM-5

Dual diagnosis

Dual-coding theory

Dump job

Dynamicism

Dysarthria

Dyscalculia

Dysfunctional family

Dysgraphia

Dyskinesia

Dyslexia

Dyspareunia

Dysphonia

Dysphoria

Dyssomnia

Dysthymia

Early childhood education

Early intervention in psychosis

Eating disorder

Echolalia

Echopraxia

Ecological psychology

Ecopsychology

Educational organization

Educational psychology

Edwards Personal Preference Schedule

Ego

Ego ideal

Ego psychology

Ego reduction

Egocentric predicament

Egocentrism

Egodystonic

Ego-dystonic sexual orientation

Egomania

Egosyntonic

Elective mutism

Electra complex

Electroencephalography

EMDR Institute

Emetophobia

Emotion

Emotion and memory

Emotional age

Emotional blackmail

Emotional conflict

Emotional contagion

Emotional dysregulation

Emotional expression

Emotional insecurity

Emotional intelligence

Emotional isolation

Emotional labor

Emotional reasoning

Empathic distress

Empathy

Empathy gap

Emptiness

Empty-chair technique

Encoding (memory)

Encopresis

Encounter group

Endogenous depression

Endogeny

Energy psychology

Engram

Entitlement

Entomophobia

Enuresis

Environmental psychology

Envy

Epiphany (feeling)

Equity theory

Ergophobia

Erikson's stages of psychosocial development

Erotomania

Erotophobia

Erythrophobia

ESFJ

Ethics and evolutionary psychology

Euphoria

European Association for Psychotherapy

Evaluation

Evolutionary developmental psychology

Evolutionary educational psychology

Evolutionary Principle

Evolutionary psychology

Exaggeration

Exclusivism

Executive functions

Exhibitionism

Existential therapy

Exogeny

Exorcism

Experimental group

Experimental method

Experimental neurosis

Experimental psychology

Experimental Psychology Society

Experimenter's bias

Explanation

Explicit memory

Exposure and response prevention

Expressed emotion

Expressive language disorder

External validity

Extraversion and introversion

Eysenck Personality Questionnaire

Face perception

Face validity

Face-ism

Facial expression

Factitious disorder

Factorial ANOVA

Faculty psychology

False awakening

Family therapy

Fantasy (psychology)

Fast mapping

Fear

Fear of flying

Feature integration theory

Feeble-minded

Feedback loop

Feelings

Female sexual arousal disorder

Femininity

Feral child

Fetal alcohol syndrome

Fight-or-flight response

Fixation (psychology)

Flashback (psychology)

Flashbulb memory

Flattery

Flooding (psychology)

Flow (psychology)

Flowerpot technique

Fluid and crystallized intelligence

Folie à deux

Folk psychology

Fooled by Randomness

Foolishness

Foot-in-the-door technique

Forensic psychology

Forgiveness

Formal operational stage

Formication

Formicophilia

Foundations of Cyclopean Perception

Four discourses

Four stages of competence

Fragile X syndrome

Free association (psychology)

Free-floating anxiety

Free-running sleep

Frigidity

Frustration

F-scale

Fugue

Functional autonomy

Functional disorder

Functional psychology

Functional symptom

Fundamental attribution error

Fundamental Interpersonal Relations Orientation

Ganser syndrome

Gaze

Gender identity

Gender identity disorder

Gender narcissism

Gender role

General adaptation syndrome

General intelligence factor

Generalized anxiety disorder

Genetic predisposition

Genie (feral child)

Genital stage

Genophobia

Geon (psychology)

Germaphobia

Geschwind–Galaburda hypothesis

Gestalt psychology

Gestalt theoretical psychotherapy

Gestalt therapy

Global aphasia

Glossophobia

Gnosology

God helmet

Grand mal epilepsy

Grandiose delusions

Grandiosity

Gratification

Gratitude

Graz School

Greed

Gregariousness

Grief

Grounding (punishment)

Group attribution error

Group dynamics

Group Dynamics (Myers-Briggs)

Group polarization

Group psychotherapy

Group synergy

Group-serving bias

Groupthink

Guilt

Guilty but mentally ill

Gustation

Gymnophobia

Gyrus

Habit (psychology)

Habituation

Hair pulling

Hakomi

Hallucination

Hallucinosis

Halo effect

Hamilton Depression Rating Scale

Hawthorne effect

Healing temple

Health and Human Services

Health psychology

Hedonism

Heffter Research Institute

Heliophobia

Hemophobia

Herpetophobia

Hidden observer

Hierarchy of needs

High IQ society

Highway hypnosis

History of psychology

Histrionic personality disorder

Holland Codes

Homophobia

Homosexual panic

Homosexuality

Hopfield net

Host (psychology)

Hostility

How the Mind Works

Hubris

Human behavior

Human bonding

Human computer interaction

Human Givens

Human multitasking

human sexuality

Humanistic psychology

Humiliation

Hwa-Byung

Hydrophobia

Hydrotherapy

Hyperactivity

Hyperactivity disorder

Hypergraphia

Hypergyny

Hyperkinetic disorder

Hypermasculinity

Hyperprosexia

Hyperreflexia

Hypersomnia

Hypertension

Hypertensive crisis

Hyperthymesia

Hyperventilation

Hypesthesia

Hypnopompic

Hypnotherapy

Hypnotic

Hypoactive sexual desire disorder

Hypochondriasis

Hypomania

Hypomanic episode

Hysteria

Hysterical neurosis

Iatrogenic illness

ICD-10

ICD-9

Id, ego, and super-ego

Idea

Idealization and devaluation

Ideas bank

Ideas of reference

Ideasthesia

Ideation

Identification (information)

Identity crisis (psychology)

Ideomotor effect

Idiot savant

Idiothetic

Image schema

Imagination

Imitation

Immediate memory

Implementation intention

Impotence

Impregnation fetish

Imprinting (psychology)

Impulse (psychology)

Impulse control disorder

Incentive salience

Incest

Incest taboo

Incompetent to stand trial

Indirect realism

Individual differences psychology

Individual psychology

Individuation

Inductive reasoning

Industrial and organizational psychology

Infantophilia

Inference

Inferiority complex

Informed consent

INFP

Infradian rhythm

Inheritance of intelligence

Inhibited male orgasm

Inhibited orgasm

Innate

Innate ideas

Inner child

Innocence

Inquiry

Insanity

Insanity defense

Insight

Insomnia

Instinct

Institute of Transpersonal Psychology

Institutionalization

Instrumental conditioning

Insult

Integral psychology

Integral theory

Integrative complexity

Intellectual disability

Intellectualization

Intelligence (trait)

Intelligence amplification

Intelligence quotient

Intelligence test

Interaction effects

Interference theory

Intergender

Intermittent explosive disorder

Internal capsule

Internal consistency

Internal locus of control

Internalized oppression

International Association of Analytical Psychologists

International Classification of Diseases

International Society for Comparative Psychology

Interpersonal and social rhythm therapy

Interpersonal psychoanalysis

Interpersonal psychotherapy

Interpersonal skills

Inter-rater reliability

Interstimulus interval

Intertwingularity

Intervention (counseling)

Interview

Intimacy

Intimate relationship

Intravenous

Intrinsic motivation

Introjection

Intromission

Introspection

Introversion

Intuition (knowledge)

Involuntary commitment

Involutional melancholia

IQ

Ironic process theory

Irrational anger

Irresistible impulse

Isolation

Jealousy

Jenkins activity survey

Johari window

Joint Commission on Accreditation of Healthcare Organizations

Journal of Applied Developmental Psychology

Journal of Health Psychology

Journal of Psychohistory

Judgement

Jungian psychology

Just-world phenomenon

Keirsey Temperament Sorter

Kharkov School of Psychology

Kinesics

Kinesthesis

Klein-Levin syndrome

Kleptomania

Klinefelter syndrome

Klismaphilia

Klüver-Bucy syndrome

Knowledge management

Kohlberg's stages of moral development

Kübler-Ross model

Labeling theory

Labile

Lability

Laboratory for Automation Psychology

Laceration

Lacrimation

Lacunar amnesia

Landolt ring

Language acquisition device

Language disorder

Lapsus

Lapsus linguae

Large Group Awareness Training

Large-group communication

Latah

Latency period

Latency stage

Latent learning

Lateral thinking

Law of effect

L-dopa

Leadership

Learned helplessness

Learning

Learning curve

Learning disabilities

Learning disability

Learning disorders

Learning organization

Learning theory (education)

Legal psychology

Lethologica

Levels-of-processing effect

Liberation psychology

Libido

Lie

Lifespring

Lifetime prevalence

Light therapy

Ligyrophobia

List of cognitive biases

List of credentials in psychology

List of emotions

List of important publications in psychology

List of psychological research methods

List of psychologists

List of psychology disciplines

List of psychology journals

List of psychology organizations

List of scientific journals in psychology

Locus of control

Loevinger's stages of ego development

Logorrhea (psychology)

Logotherapy

Logovisual technology

Loner

Longitudinal study

Looking glass self

Loose associations

Loss aversion

Lovaas technique

Love styles

Lovemap

Low frustration tolerance

Lucid dream

Lüscher color test

MacDonald triad

Machiavellianism scale

Madonna-whore complex

Magical thinking

Main effect

Major depression

Major depressive disorder

Making excuses

Maladaptive daydreaming

Malignant narcissism

Malingering

Man and His Symbols

Mania

Manic episode

Manic-depressive illness

Manipulation

Marital therapy

Marriage guidance

Martyr complex

Masculinity

Masking (personality)

Maslow's hierarchy of needs

Masochistic personality disorder

Mass hysteria

Maternal deprivation

Mathematical psychology

Mathematics disorder

Maturation and environmentalism

McLean Hospital

Mean World Syndrome

Measure of central tendency

Medical model

Medical psychology

Megalomania

Melancholia

Memory and aging

Memory augmentation

Memory consolidation

Memory effect

Memory inhibition

Memory suppression

Memory-prediction framework

Mental age

Mental block

Mental calculation

Mental confusion

Mental disorder

Mental function

Mental health

Mental health consumer

Mental health disorders

Mental illness

Mental management

Mental model

Mental status

Mental status examination

Mentalism (psychology)

Mentally ill

Mentoring

Mesmerism

Mesomorphic

Messiah complex

Metabolism

Metabolite

Metapsychology

Microexpression

Middle age

Mid-life crisis

Milgram experiment

Milieu therapy

Mind control

Mind Dynamics

Mind map

Mind-body dualism

Mind-body problem

Mindfulness (psychology)

Mind's eye

Mindset

Minimisation (psychology)

Minnesota Multiphasic Personality Inventory

Mirror stage

Mixed anxiety-depressive disorder

Mixed state (psychiatry)

MMPI-2

Mnemonic link system

Mnemonics

Mnemonist

Mob psychology

Model (abstract)

Model of hierarchical complexity

Modelling (psychology)

Monomania

Mood (psychology)

Mood disorder

Mood swing

Moral psychology

Moral reasoning

Moral treatment

Morbid jealousy

Moron (psychology)

Mortido

Motion illusion

Motivation

Motor coordination

Motor skills disorder

Movement context in handwriting

Mozart Effect

Multidisciplinary Association for Psychedelic Studies

Multi-infarct dementia

Multilevel model

Multimodal therapy

Multimodal Therapy

Multiple personality disorder

Multiple-complex Developmental Disorder

Multnomah Community Ability Scale

Münchausen syndrome

Münchausen syndrome by proxy

Music psychology

Mutism

Myers-Briggs Type Indicator

Mysophilia

Mysophobia

Mythomania

N-Affil

Napoleon complex

Narcissism

Narcissism of small differences

Narcissistic parent

Narcissistic personality disorder

Narcissistic rage

Narcosynthesis

Narrative therapy

Naturalistic observation

Necrophobia

Need

Negative reinforcement

Negative symptoms

Negativistic personality disorder

Neglect

Neo-Freudian

Neophobia

Neo-Piagetian theories of cognitive development

Neurocognition

Neurofeedback

Neuro-linguistic programming

Neurological disorder

Neuropsychological test

Neuropsychology

Neuroscience

Neurosis

Neurosyphilis

Neuroticism Extraversion Openness Personality Inventory

Night owl (person)

Nightmare

Nightmare disorder

Nonparametric test

Norm (sociology)

N-Pow

NT (temperament)

Nurturant parent model

Nurture

Nyctophobia

Nymphophilia

Object permanence

Objective test

Observational learning

Obsessive Relational Intrusion (ORI)

Obsessive-compulsive disorder

Obsessive-compulsive personality disorder

Occupational health psychology

Occupational psychology

Occupational psychosis

Occupational therapy

Octave illusion

Oedipus complex

One-upmanship

Open relationship

Operant behavior

Operant conditioning

Operations research

Opponent-process theory

Opportunism

Oppositional defiant disorder

Optimal distinctiveness theory

Oral stage

Ordinal numerical competence

Organizational citizenship behavior

Organizational communication

Organizational psychology

Orgone

Orientation (mental)

Ornithophobia

Osmophobia

Overjustification effect

Overlearning

Pain and pleasure

Pain disorder

Pair by association

Pairwise comparison

Palilalia

Panic attack

Panic disorder

Papert's principle

Paradoxical intention

Parallel play

Paranoia

Paranoid disorder

Paranoid personality disorder

Paranoid schizophrenia

Paranoid-schizoid position

Paraphilia

Paraphrenia

Parapsychology

Paraskevidekatriaphobia

Parasuicide

Parenting (Myers-Briggs)

Paresthesia

Parosmia

Passion (emotion)

Passive–aggressive behavior

Password psychology

Pastoral counseling

Pathogenic theory of schizophrenia

Pathognomy

Pathological gambling

Pathological lying

Patience

PDD not otherwise specified

Pedophile

Pedophilia

Penis envy

People skills

Perception

Perceptual psychology

Perfectionism (psychology)

Performance anxiety

Performance psychology

Persecution complex

Persecutory delusions

Person centered planning

Personal boundaries

Personal commitment

Personal construct psychology

Personal construct theory

Personality alteration

Personality Assessment Inventory

Personality disorder

Personality psychology

Personality tests

Personality trait

Person-centered therapy

Perspective (cognitive)

Persuasion

Pervasive developmental disorders

Phagophobia

Phallic stage

Phantom rings

Phenomenology (psychology)

Phenotype

Phenotypic trait

Philippine psychology

Philomath

Philosophy of mind

Philosophy of psychology

Phobia

Phonological disorder

Phonology

Photopic vision

Phrenology

Physiological psychology

Piaget's theory of cognitive development

Pick's disease

Picture thinking

Pillow talk

Piquerism

Pituitary gland

Placebo effect

Planning

Plateau phase

Platykurtic

Play therapy

Pleasure principle (psychology)

Poker psychology

Polarization (psychology)

Political psychology

Popular psychology

Positive Mental Attitude

Positive psychology

Positive reinforcement

Postcognitivism

Post-cognitivist psychology

Post-purchase rationalization

Post-traumatic embitterment disorder

Post-traumatic stress disorder

Postvention

Potential development level

Poverty of speech

Power (sociology)

Power Law of Practice

Practical equine psychology

Prairie madness

Praise

Pre- and perinatal psychology

Preconscious

Prediction

Predictive validity

Prejudice

Premature ejaculation

Premenstrual dysphoric disorder

Preoperational stage

Prescriptions regarding gender roles

Presenile dementia

Primacy effect

Primal therapy

Privileged communication

Proactive inhibition

Probability of error

Probands

Problem finding

Problem shaping

Problem solving

Problem-based learning

Process Oriented Psychology

Process Psychology

Procrastination

Prodrome

Professional practice of behavior analysis

Program evaluation

Programmed learning

Projective identification

Projective test

Pronoia (psychology)

Pronoun reversal

Properception

Propinquity

Proposition

Propositional attitude

Prosopagnosia

Prospect theory

Prospection

Protoself

Proxemics

Prudence

Pseudocertainty effect

Pseudologia

Psyche (psychology)

Psychedelic

Psychiatric hospital

Psychiatrist

Psychic driving

Psychoacoustics

Psychoactive

Psychoactive drug

Psychoanalysis

Psychoanalyst

Psychoanalytic feminism

Psychoanalytic film theory

Psychoanalytic theory

Psychobiography

Psychodrama

Psychodynamic theory

Psychodynamic therapy

Psychodynamics

Psychogenesis

Psychogenic amnesia

Psychogenic pain

Psychogenic polydipsia

Psychogram

Psychohistory

Psycholinguist

Psycholinguistics

Psychological abuse

Psychological adaptation

Psychological assessment

Psychological dependency

Psychological identity

Psychological manipulation

Psychological pain

Psychological projection

Psychological repression

Psychological research methods

Psychological resilience

Psychological Review

Psychological statistics

Psychological testing

Psychological tests

Psychological trauma

Psychological types

Psychology of combat

Psychology of learning

Psychology of Monogamy

Psychology of previous investment

Psychology of programming

Psychology of reasoning

Psychology of religion

Psychology Today

Psychology, Philosophy and Physiology

Psychometrics

Psychomotor agitation

Psychomotor retardation

Psychoneuroimmunology

Psychonomics

Psychoorganic syndrome

Psychopathology

Psychopathy

Psychopharmacology

Psychophysics

Psychophysiology

Psychosexual stages

Psychosis

Psychosomatic disorders

Psychotherapy

Psychotic depression

Psychotropic medication

PsycINFO

Puer Aeternus

Punishment

Punitive psychiatry in the Soviet Union

Quantitative psychological research

Quantitative psychology

Quantitative trait locus

Quantum Psychology

Radical behaviorism

Radical Psychology Network

Radiophobia

Rage (emotion)

Random assignment

Rape trauma syndrome

Rapport

Rashomon effect

Rational choice theory

Rational emotive therapy

Rational-emotive therapy

Rationality

Rationalization (psychology)

Raynaud's disease

Reachback

Reactance (psychology)

Reaction formation

Reactive attachment disorder

Reactivity (psychology)

Reading (activity)

Reality distortion field

Reality principle

Reasoning

Reciprocal liking

Reciprocity (social psychology)

Recklessness (psychology)

Recluse

Recollection

Recovery International

Recurring dream

Re-evaluation Counseling

Referent power

Reframing

Regression

Regression analysis

Regulatory Focus Theory

Rehabilitation (neuropsychology)

Rehabilitation counseling

Reinforcement

Reinforcer

Rejection (emotion)

Relational aggression

Relational disorder

Relational frame theory

Relationship counseling

Relationships (Myers-Briggs)

Religious instinct

Reminiscence

Remorse

Renfield's syndrome

Repetition compulsion

Representations

Representativeness heuristic

Repression

Rescorla-Wagner model

Research methods

Resentment

Residual schizophrenia

Resistance (psychology)

Respondent conditioning

Retroactive inhibition

Retroactive interference

Retrograde amnesia

Retrospective memory

Reuptake

Reverse learning

Reverse psychology

Rhetoric

Righteous indignation

Risky shift

Ritualization

Role reversal

Role theory

Role-playing

Rorschach inkblot test

Rosenthal effect

Rotter Incomplete Sentence Blank

Rousseau Institute

Sadistic personality disorder

Sadomasochism

Safety in numbers

Sander illusion

Sapience

Scale (social sciences)

Schadenfreude

Schedules of reinforcement

Schema (psychology)

Schizoaffective disorder

Schizoid personality disorder

Schizophrenia

Schizophrenics Anonymous

Schizophreniform disorder

Schizotypal personality disorder

Scholastic Aptitude Test

School phobia

School refusal

Science and Consciousness Review

Scientific control

Seasonal affective disorder

Secondary gain

Security blanket

Selective abstraction

Selective distortion

Self (Jung)

Self (psychology)

Self actualization

Self control

Self efficacy

Self handicapping

Self propaganda

Self psychology

Self serving bias

Self-actualization

Self-awareness

Self-concept

Self-consciousness

Self-criticism

Self-deception

Self-defeating personality disorder

Self-determination theory

Self-disclosure

Self-efficacy

Self-esteem

Self-esteem functions

Self-help

Self-injury

Selfishness

Self-knowledge

Self-loathing

Self-monitoring

Self-parenting

Self-perception theory

Self-pity

Self-punishment

Self-realization

Self-regulated learning

Semantic dementia

Semantic dyslexia

Semantic memory

Semantics

Senile dementia

Senile plaques

Sensation (psychology)

Sense of time

Sensitivity (human)

Sensory adaptation

Sensory gating

Sensory memory

Sensory neuroscience

Sensory preconditioning

Sensory threshold

Sentience

Separation anxiety disorder

Serial position effect

Serial sevens

Sex-reassignment surgery

Sexual arousal

Sexual arousal disorders

Sexual aversion disorder

Sexual desire

Sexual deviation

Sexual disorders

Sexual dysfunction

Sexual fetishism

Sexual masochism

Sexual orientation

Sexual response cycle

Sexual sadism

Shadow (psychology)

Shame

Shaping (psychology)

Shell shock

Shock value

Short term memory

Shyness

Sibling

Siege mentality

Sigmund Freud Archives

Silva Method

Similarity (psychology)

Simon effect

Simplicity theory

Simulated consciousness

Simulated pregnancy

Simulation heuristic

Sitophobia

Situational awareness

Six Thinking Hats

Size-weight illusion

Skinner box

Sleep-learning

Sluggish cognitive tempo

Sluggishly progressing schizophrenia

Smart mob

Social anxiety

Social anxiety disorder

Social cognition

Social desirability

Social disruption

Social distance

Social distance scale

Social facilitation

Social group

Social influence

Social inhibition

Social interaction

Social learning theory

Social loafing

Social neuroscience

Social norm

Social proof

Social psychology (psychology)

Social psychology (sociology)

Social rejection

Social rhythm therapy

Social role

Social skills

Social statistics

Social status

Social stigma

Social support

Socialization

Society of Mind theory

Socioeconomic status

Sociometry

Socionics

Sociosexual orientation

Sodomy

Solitary confinement

Soma

Somatization disorder

Somatoform disorder

Somatotherapy

Somatotype and constitutional psychology

Spatial empathy

Spatial memory

Spatial-temporal reasoning

Speaker recognition

Specific phobia

Specific social phobia

Speech act

Speech perception

Speed reading

Spiral dynamics

Spontaneous recovery

Sport psychology

Stage fright

Stage theory

Stages of faith development

Stanford-Binet

State-dependent learning

State-dependent memory

Steppingstone theory

Stereotypes

Stereotypic movement disorder

Stigmatic/eligibilic paraphilia

Stimming

Stimulus generalization

Stir crazy (condition)

Stockholm syndrome

Storage (memory)

Strategic planning

Stream of consciousness (psychology)

Stress (medicine)

Stress management

Stressor

Structural communication

Structural ritualization theory

Structuralism

Structure-agency debate

Structured interview

Study Skills

Subjective reality

Sublimation (psychology)

Subliminal advertising

Subliminal perception

Submission

Substance abuse

Substance Abuse and Mental Health Services Administration

Substance dependence

Substance intoxication

Substance-related disorder

Subvocalization

Suffering

Suicidal ideation

Suicide

Suicide treatment

Suicide watch

Suicidology

Superego

Superficial charm

Superiority complex

Superman complex

Superordinate goals

Surprise (emotion)

Swept-plane display

Sycophancy

Syllogism

Sylvia Plath effect

Symbolic violence

Sympathetic nervous system

Sympathy

Synectics

Synesthesia

Systematic desensitization

Systems psychology

Systems thinking

T test

Taboo

Tactile

Taijin kyofusho

Talking cure

Taphophobia

Target fixation

Taunting

Technophobia

Telepathy

Temperament

Terdekaphobia

Ternus illusion

Test (assessment)

Test-retest reliability

Texas State Board of Examiners of Psychologists

T-groups

Thalamus

The American Psychologist

The Blank Slate

The Fifth Discipline

The g Factor: General Intelligence and Its Implications

The g Factor: The Science of Mental Ability

The Imaginary

The Principles of Psychology

The Real

The Retreat

The Social Animal

The Symbolic

The Third Wave

The Wisdom of Crowds

Thematic Apperception Test

Theophylline

Theoretical psychology

Theory of Cognitive development

Theory of Constraints

Theory of Deadly Initials

Theory of mind

Theory of multiple intelligences

Therapeutic community

Thinking

Thinking Processes (Theory of Constraints)

Thought

Thought broadcasting

Thought disorder

Thought experiment

Thought Field Therapy

Thought insertion

Thought withdrawal

Thought-terminating cliché

Thousand-yard stare

Three Essays on the Theory of Sexuality

Timeline of psychology

Toilet training

Token economy

Tonic–clonic seizure

Touch illusion

Tourette syndrome

Traffic psychology

Trait theory

Transactional analysis

Transderivational search

Transduction (psychology)

Transfer (propaganda)

Transfer of learning

Transfer of training

Transference

Transference neurosis

Transpersonal

Transpersonal psychology

Transsexual

Transsexualism

Transvestic fetishism

Tree of Knowledge System

Triarchic theory of intelligence

Triskaidekaphobia

Trisomy

Trollope ploy

True experiment

Trust (sociology)

Trust metric

Trypanophobia

Tumescence

Twin study

Type A and Type B personality theory

Ultradian

Unconditional positive regard

Unconditioned response

Unconditioned stimulus

Unconscious mind

Understanding

Undifferentiated schizophrenia

Unipolar depression

United Kingdom Council for Psychotherapy

Universal law of generalization

Universalization

Valence (psychology)

Value (personal and cultural)

Value theory

Vascular dementia

Vegetotherapy

Vertical thinking

Victim blaming

Victim playing

Victimisation

Victimology

Vienna Psychoanalytic Society

Vigilance (psychology)

Visual learning

Visual thinking

Volition (psychology)

Voodoo death

Voyeurism

Vulnerability

WAIS-III

Wakefulness

Waking states

Waxy flexibility

Weapon focus

Web Experimental Psychology Lab

Wechsler adult intelligence scale

Wechsler Intelligence Scale for Children

Wechsler Preschool and Primary Scale of Intelligence

William Alanson White Institute

Wisdom of crowds

Wise old man

Withdrawal symptoms

Witzelsucht

Womb envy

Word salad

Working memory

Working through

World Federation for Mental Health

Xenophobia

Yerkes-Dodson law

Zeitgeist

Zener cards

Zero-defects mentality
